# Combined berberine and probiotic treatment as an effective regimen for improving postprandial hyperlipidemia in type 2 diabetes patients: a double blinded placebo controlled randomized study

**DOI:** 10.1080/19490976.2021.2003176

**Published:** 2021-12-20

**Authors:** Shujie Wang, Huahui Ren, Huanzi Zhong, Xinjie Zhao, Changkun Li, Jing Ma, Xuejiang Gu, Yaoming Xue, Shan Huang, Jialin Yang, Li Chen, Gang Chen, Shen Qu, Jun Liang, Li Qin, Qin Huang, Yongde Peng, Qi Li, Xiaolin Wang, Yuanqiang Zou, Zhun Shi, Xuelin Li, Tingting Li, Huanming Yang, Shenghan Lai, Guowang Xu, Junhua Li, Yifei Zhang, Yanyun Gu, Weiqing Wang

**Affiliations:** a Department of Endocrine and Metabolic Diseases, Shanghai Institute of Endocrine and Metabolic Diseases, Ruijin Hospital, Shanghai Jiao Tong University School of Medicine, Shanghai, China; b Shanghai National Clinical Research Center for Metabolic Diseases, Key Laboratory for Endocrine and Metabolic Diseases of the National Health Commission of the Pr China, Shanghai National Center for Translational Medicine, Ruijin Hospital, Shanghai Jiao Tong University School of Medicine, Shanghai, China; c BGI-Shenzhen, Shenzhen, China; d Laboratory of Genomics and Molecular Biomedicine, Department of Biology, University of Copenhagen, Copenhagen, Denmark; e Dalian Institute of Chemical Physics, Chinese Academy of Science, Dalian, China; f Ren Ji Hospital, Shanghai Jiao Tong University School of Medicine, Shanghai, China; g Department of Endocrinology, The First Affiliated Hospital of Wenzhou Medical University, Zhejiang Province, China; h Nanfang Hospital, Southern Medical University, Guangdong Province, China; i Tong Ren Hospital, Shanghai Jiao Tong University School of Medicine, Shanghai, China; j Department of Endocrinology, Central Hospital of Minhang District, Shanghai, China; k Department of Endocrinology, Qilu Hospital of Shandong University, Shandong Province, China; l Department of Endocrinology, Fujian Provincial Hospital, Fujian Province, China; m Department of Endocrinology, Shanghai Tenth People’s Hospital of Tong Ji University, Shanghai, China; n Department of Endocrinology, Xuzhou Central Hospital, Jiangsu Province, China; o Xin Hua Hospital, Shanghai Jiao Tong University School of Medicine, Shanghai, China; p Chang Hai Hospital, Second Military Medical University, Shanghai, China; q Shanghai First People’s Hospital, Shanghai Jiao Tong University School of Medicine, Shanghai, China; r James D. Watson Institute of Genome Sciences, Hangzhou, China; s Johns Hopkins University School of Medicine, Baltimore, Maryland, USA

**Keywords:** Type 2 diabetes, probiotics, berberine, dyslipidemia, postprandial lipidemia, gut microbiome

## Abstract

Non-fasting lipidemia (nFL), mainly contributed by postprandial lipidemia (PL), has recently been recognized as an important cardiovascular disease (CVD) risk as fasting lipidemia (FL). PL serves as a common feature of dyslipidemia in Type 2 Diabetes (T2D), albeit effective therapies targeting on PL were limited. In this study, we aimed to evaluate whether the therapy combining probiotics (Prob) and berberine (BBR), a proven antidiabetic and hypolipidemic regimen via altering gut microbiome, could effectively reduce PL in T2D and to explore the underlying mechanism. Blood PL (120 min after taking 100 g standard carbohydrate meal) was examined in 365 participants with T2D from the Probiotics and BBR on the Efficacy and Change of Gut Microbiota in Patients with Newly Diagnosed Type 2 Diabetes (PREMOTE study), a random, placebo-controlled, and multicenter clinical trial. Prob+BBR was superior to BBR or Prob alone in improving postprandial total cholesterol (pTC) and low-density lipoprotein cholesterol (pLDLc) levels with decrement of multiple species of postprandial lipidomic metabolites after 3 months follow-up. This effect was linked to the changes of fecal *Bifidobacterium breve* level responding to BBR alone or Prob+BBR treatment. Four *fadD* genes encoding long-chain acyl-CoA synthetase were identified in the genome of this *B. breve* strain, and transcriptionally activated by BBR. *In vitro* BBR treatment further decreased the concentration of FFA in the culture medium of *B. breve* compared to vehicle. Thus, the activation of *fadD* by BBR could enhance FFA import and mobilization in *B. breve* and diliminish the intraluminal lipids for absorption to mediate the effect of Prob+BBR on PL. Our study confirmed that BBR and Prob (*B. breve*) could exert a synergistic hypolipidemic effect on PL, acting as a gut lipid sink to achieve better lipidemia and CVD risk control in T2D.

## Introduction

Hyperlipidemia is a major risk factor for atherosclerotic cardiovascular diseases (ASCVDs),^1^ particularly when combined with hyperglycemia and Type 2 Diabetes (T2D).^2–4^ Current diagnose criteria and treatment target are based on evaluating fasting lipidemia (FL). However, increasing evidence has supported that a high level of non-fasting lipidemia (nFL), mainly constituted by postprandial lipidemia (PL), is also an important CVD risk factor^5–8^ and multiple countries are currently changing their guidelines toward a consensus on measuring a lipid profile for cardiovascular risk prediction in the non-fasting state.^7–11^ Individuals are mainly in a non-fasting state during a regular 24-hour cycle, and non-fasting samples would simplify blood sampling and minimize the risk of hypoglycemia particularly for individuals with diabetes.^12^ Furthermore, postprandial hyperlipidemia is a common feature of insulin-resistant diabetes patients^13–15^ and has been recommended for evaluating T2D-related ASCVDs.^16,17^ Therefore, managing both FL and PL in T2D should be required to achieve better control for overall lipidemia and CVD risks, whereas, except of Niemann-Pick C1-like 1 (NPC1L1) inhibitor (Ezetimibe),^18^ there is few regimens developed for targeting PL or both.

Unlike FL that are mainly derived from liver-derived lipoproteins, PL alterations are constituted by intestinal lipid absorption, lipoprotein secretion and chylomicron production,^15^ and recently have been found to be tightly related with gut microbiota alterations,^19^ indicating that a different avenueto develop druggable targets for PL from that for FL. The intestinal microbiota is involved in host intestinal lipid absorption and lipid metabolism regulations in other metabolic organs contributing to host lipidomic profile alterations.^20–28^ However, in contrast to the piled-up evidence of associations, the intricate crosstalk between the microbiota and host circulation lipidomic alterations is far from fully elucidated.

Berberine (BBR), a plant alkaloid extracted from the Chinese herbal medicine *Coptis chinensis* (Huanglian), is known to elevate liver LDL uptake and adipose browning.^29–33^ Recently the impact of BBR on gut microbiota has been recognized and linked to its effect on metabolic disorders. Probiotics, such as strains from *Bifidobacterium*, improve dyslipidemia, via cholesterol binding, host intestinal absorption blocking, or altering host bile acid signaling.^34–36^ Both BBR and probiotics are defined as nutraceutical hypolipidemic agents, considering their effects on lowering FL.^37–43^ It is unknown if BBR or probiotics also lowering the nFL, whereastheir effects on gut microbiome suggested that they might be suitable candidate measures for treating PL. In “Probiotics and BBR on the Efficacy and Change of Gut Microbiota in Patients with Newly Diagnosed Type 2 Diabetes (PREMOTE) trial,”^33^ we confirmed that the antidiabetic effect of BBR could be mediated by its effect on gut microbial bile acid metabolism, and supplementation with probiotics can improve the hypoglycemic effect of BBR in participants older than 50. However, probiotics cannot improve the effect of BBR on lowering fasting lipidemia either in the whole cohort or in aged subgroup. This prompted us to ask how the combination treatment of BBR and probiotics, or either one could exert benefit on lowering PL, and whether their impact on gut microbiota could contribute to this effect .

To achieve this aim, in this study we compared lipidemia in postprandial blood samples collected from T2D patients assigned to the four treatment groups in the PREMOTE trial: placebo (Plac), probiotics alone (Prob), berberine alone (BBR), and probiotics combined with berberine (Prob+BBR). We further explored the potential underlying mechanism via multi-omics and comparative genomic analysis with *in vitro* culture experimental verification.

## Methods

### Clinical study

The PREMOTE study, a randomized, double-blind, placebo-controlled clinical trial in 20 medical centers in China (ClinicalTrials.gov number, NCT02861261), enrolled newly diagnosed T2D patients from August 18, 2016, to July 18, 2017. The PREMOTE trial evaluated glycemic control as the primary outcome and lipidaemia control as the secondary outcome.^33^ The primary outcome and its related microbiota mechanism have been previously published, showing that the combined treatment of BBR and probiotics showed superior hypoglycemic effect to BBR or probiotics alone in subjects aged ≥50 years. BBR alone and Prob+BBR showed similar effects in reducing fasting lipidemia.

This lipidomic study included 365 of the 409 enrolled participants from the PREMOTE trial based on the availability of postprandial lipid measurements before and after the 3-month intervention. Metagenomic sequencing data for the 1,192 fecal samples can be accessed from the National Center for Biotechnology Information BioProject Database with the dataset accession number PRJNA643353.

The detailed inclusion and exclusion criteria are listed in the online protocol and the previous study.^33^ In brief, drug-naïve participants were newly diagnosed with T2D according to the 1999 World Health Organization criteria, and included patients of both sexes, aged between 20 and 70 years, with a body mass index (BMI) between 19.0 and 35.0 kg/m^2^. All enrolled participants had HbA1c ≥6.5% and ≤10.0% and fasting plasma glucose ≥7.0 mmol L^−^1 (126.1 mg/dl) and ≤13.3 mmol L^−1^(239.6 mg/dl). Patients were excluded from the study if they had severe liver dysfunction, impaired renal function, severe organic heart diseases or heart failure (New York Heart Association class (NYHA) grade of heart function ≥ III), psychiatric disease, severe infection, severe anemia, neutropenia, or history of acute diabetic complications and are allergic to gentamicin, other amino glycoside antibiotics, berberine, or other probiotics.

All participants provided written informed consent. The study was approved by each institution’s human participant ethics committee at each participating center. The participants were randomly assigned into one of the following four groups in a 1:1:1:1 ratio as follows: BBR (0.6 g per 6 pills, twice daily before a meal) plus probiotics (4 g per 2 strips of powder, once daily at bedtime) (Prob+BBR), probiotics plus placebo (Prob), BBR plus placebo (BBR), or placebo plus placebo (Plac). Treatments were administered for 12 weeks, and patients visited the center every 4 weeks until the end of the study. The stratified randomization was achieved by utilizing a validated Interactive Web-based Response System (IWRS) as reported previously. The study personnel and participants were blinded to the assignment of treatment groups. BBR was manufactured by Northeast Pharmaceutical Group Co., Ltd., Shenyang, Liaoning, China. The multi-strain probiotic products containing nine proprietary strains of probiotics were produced by Shanghai Jiaoda Onlly Co., Ltd., Shanghai, China (*Bifidobacterium longum* CGMCC No. 2107; *Bifidobacterium breve* CGMCC No. 6402; *Lactococcus gasseri* CGMCC No. 10758; *Lactobacillus rhamnosus* CNCM I-4474; *Lactobacillus salivarius*CGMCC No. 6403; *Lactobacillus crispatus* CGMCC No. 6406; *Lactobacillus plantarum*; CGMCC No. 1258; *Lactobacillus fermentum* CGMCC No. 6407; and *Lactobacillus casei*CNCM I-4458), and each sachet contains ≥50 billion CFU of live, freeze-dried bacteria. The placebos were provided along with the medications, details have been published in previous study.^33^

Dyslipidemia was defined according to the US National Cholesterol Education Program/Adult Treatment Panel III (NCEP/ATP III) criteria as total fasting levels of cholesterol ≥240 mg/dl, triglycerides ≥200 mg/dl, HDL-c ≤ 40 mg/dl, LDL-c ≥ 160 mg/dl and/or taking lipid-lowering medications,^44^and the others were defined as eulipidemia.

### Postprandial lipid measures

In this study, we measured lipidemia of postprandial blood samples, which were drawn 120 min after taking 100 g standard carbohydrate meal provided by China Food Limited, COFCO (Beijing, China).^45^ The PL measurements were performed in the central laboratory of Ruijin Hospital. Postprandial plasma total cholesterol (pTC), triglycerides (pTGs), high-density lipoprotein cholesterol (pHDLc), and low-density lipoprotein cholesterol (pLDLc) were measured by the cholesterol oxidase method, glycerophosphate oxidase-peroxidase method, polyanion polymer/detergent method and solubilization method with an autoanalyzer (AU5800; Beckman Coulter, CA, USA). Other biochemical measures were taken as in our previous study.^33^

### Metabolomic measures

#### 1. Materials and Reagents

The organic solvents used in this experiment, including acetonitrile and methanol, were purchased from Merck (Darmstadt, Germany) at high-performance liquid chromatography (HPLC) grade. Formic acid (HPLC grade) was obtained from Sigma-Aldrich (St. Louis, MO, USA), and ammonium bicarbonate was obtained from Fluka (CH, Buchs, Switzerland) by liquid chromatography–mass spectrometry (LC-MS). Ultrapure water was obtained from a Milli-Q water system (Millipore, Billerica, USA).

#### 2. Sample preparation

A 100 μL serum sample was mixed with 400 μL extraction solvent in 2 mL centrifuge tubes. This extraction solvent was made with methanol containing 0.1 µg/mL carnitine C2:0-d3, 0.1 µg/mL carnitine C10:0-d3, 0.15 µg/mL carnitine C16:0-d3, 0.75 µg/mL LPC 19:0, 2.5 µg/mL Fatty acid (FFA) C16:0-d3, 2.5 µg/mL FFA C18:0-d3, 4.25 µg/mL tryptophan-d5, 3.6 µg/mL phenylalanine-d5, 0.75 µg/mL SM (d18:1/12:0), 2 µg/mL choline-d4, and 0.1 µg/mL. After vertexing and centrifugation, two parts of 180 μL supernatant were freeze-dried and then stored at −80°C. Before analysis, 50 μL acetonitrile/water (1:4) solvent was added to each sample for reconstitution.

To assess the stability of the analysis process, quality control (QC) samples from the mixture of equal volumes of all serum samples were prepared using the same method as the serum samples and analyzed once after every ten serum samples.

#### 3. Non-targeted LC-MS Methods

As described previously,^46–48^ a Vanquish UPLC-Q Exactive instrument (Thermo Fisher Scientific, Rockford, IL, USA) was used for LC-MS analysis.

In positive mode, a Waters BEH C8 column (50 mm × 2.1 mm, 1.7 μm, Waters, Milford, MA) was used for LC separation. The oven temperature was 60°C, and the flow rate was 0.4 mL/min. The autosampler temperature was set at 10°C, and the injection volume was 5 μL. Phase A was ultrapure water with 0.1% formic acid, and phase B was acetonitrile with 0.1% formic acid. The gradient programme was started from 5% B and maintained for 0.5 min, then increased to 40% B for 1.5 min, continued to 100% B for 6 min, maintained at 100% B for 2 min, returned to 5% B for 0.1 min and equilibrated for 2.5 min.

In negative mode, LC separation was achieved by an ACQUITY UPLC HSS T3 column (100 mm × 2.1 mm, 1.8 μm, Waters, Milford, MA). The oven temperature was 50°C, and the flow rate was 0.35 mL/min. Phase A was ultrapure water with 6.5 mM ammonium bicarbonate, and phase B was 95% methanol/water solvent with 6.5 mM ammonium bicarbonate. The gradient program was started from 0% B and maintained for 1 min, increased to 40% B for 2 min, further increased to 100% B for 13 min, maintained at 100% B for 5 min, returned to 0% B for 0.1 min and equilibrated for 2.9 min.

A 7.0E4 resolution MS full scan mode with a scan range of m/z 70–1050 was applied for analysis. The spray voltage was 3.5 kV for positive mode and 3.00 kV for negative mode. The capillary temperature was 300°C, and the aux gas heater temperature was 350°C. The sheath gas and aux gas were 45 and 10 (in arbitrary units).

#### 4. Data processing

Nontargeted LC-MS data from multiple runs were extracted and aligned by TraceFinder 3.2 (Thermo Fisher Scientific, USA). The intensity of each retained peak was normalized using one of the internal standards. An intra-laboratory database including approximately 2000 metabolites was used to identify the metabolites in nontargeted metabolic profiling by retention time and MS1 and MS2 information.^49^ A total of 157 lipid-related metabolites were identified (details in Data Set 1 and Date Set 2).

QC samples were used to evaluate the reproducibility of the metabolomics analysis with the use of internal standard calibration (Figure S1). Furthermore, the reproducibility of the metabolite ions was also evaluated with relative standard deviation (RSD%) in the 125 QC samples: among the 157 identified metabolite ions, 98.7% of ions had an RSD% less than 30% (Figure S2).

### Metagenomics study

#### 1. Metagenomics sequencing

High-quality non-human metagenomic data (100 bp paired-end reads, BGISEQ-500 platform) and microbial profiling at the species and Kyoto Encyclopedia of Genes and Genomes (KEGG) Orthology (KO) level of all samples in this project were obtained from our previous study of the PREMOTE trial.^33^ Differentially enriched KEGG pathways between groups were identified according to the reporter Z scores of all detected KOs in the given pathway.^50^

#### 2. Genome sequencing and de novo assembly of the genomes of the ingested probiotic strains

Bacterial genomic DNA was sheared randomly to construct three read libraries with lengths of 250 bp by a Bioruptor ultrasonicator (Diagenode, Denville, NJ, USA) and physicochemical methods. The paired-end fragment libraries with an insert size of 270 bp were sequenced according to the Illumina HiSeq 4000 system protocol at the Beijing Genomics Institute (Shenzhen, China). Low quality raw reads (>40% of the bases with Q value ≤20 or containing >10% ambiguous bases) were discarded. The remaining high-quality reads were assembled to form scaffolds using SOAP denovo v1.05 software. A summary of the genome assembly statistics for each genome is provided in Table S1.

#### 3. Annotation of genes coding enzymes involved in lipid metabolism

The 9 assembled genomes of ingested probiotic strains contained in the probiotic formula and 1,520 high-quality genomes from cultivated human gut bacteria^51^ were functionally annotated to identify genes encoding lipid metabolism-related enzymes. All coding genes were translated into amino acid sequences to run BLASTX against the KEGG (version 76) pathway database (http://www.genome.jp/kegg) at an E-value threshold of 1.0E-5 and an identity threshold of 60%. All annotations were based on the best BLASTX hits. Among the 9 probiotic genomes, a total of 73 genes were annotated as functional genes encoding lipid metabolism-related enzymes, and most of the assigned sequences (94.5%, 69/73) had at least 90% sequence identity to reference sequences (Data Set 3).

### *In vitro* growth experiment of *B. breve* and *E. coli*

The *B. breve* 6402 was derived from the China General Microbiological Culture Collection Center and cultured in a strain‐specific medium (seen in Table S2). The *E. coli* MG1655 was cultured in lysogeny broth medium (Sangon Biotech, Shanghai, China). Both strains were incubated in an anaerobic chamber (Whitley A35 anaerobic workstation, Don Whitley Scientific, UK) with 5% hydrogen, 10% carbon dioxide, and 85% nitrogen at 37°C. For the growth curve experiment, we seeded *B. breve 6402* and *E. coli* MG1655 at 10% and 5%, respectively, in a volume of 5 ml media with different concentrations of BBR (0, 1.56, 3.125, 6.25, 12.5, and 25 μg ml^−1^) and cultured for 14 hours. The OD600 of the bacterial culture was measured every 2 hours with a plate reader (VarioskanFlash, Thermo Scientific, MA, USA).

For *in vitro* assaying bacterial *fadD* expression and non-esterified fatty acids (NEFAs) consumption, *B. breve 6402* were seeded at 10% in media and cultured for 10 h, then treated with BBR (6.25 μg ml^−1^) for 4 h in either blank or LA (linolenic acid, at final concentration of 1 mg ml^−1^) containing conditioned media. The NEFAs content of the media was measured with colorimetric assays (LabAssay NEFA, Wako, Japan). Stock solutions of LA (#60-33-3, MedChemExpress NJ, USA) were prepared at 10 mg ml^−1^with 2% (w/v) Tween 80 after homogenization by vertexing 2100 rpm during 150 s (separated with three intervals of 30 s).

### Real-time quantitative RT-PCR

RT-PCR was used to determine the relative levels of gene transcription in *B. breve*. Total *B. breve* RNA was extracted using an Eastep Super Total RNA Extraction Kit (Promega, Shanghai, China) and reverse transcribed to cDNA with a Reverse Transcription System Kit (Promega, Madison, USA) according to the manufacturer’s protocols. Real-time PCR amplification and detection were performed using SYBR Green II Master Mix (TaKaRa, Kusatsu, Japan) on a LightCycler 480 (Roche Applied Science, Indianapolis, USA). The sequences of primers used for four *fadD* genes are listed in Table S3. The gene expression levels were calculated and normalized to the levels of 16S rRNA.

### Statistical analysis

Statistical analyses of clinical data were performed using SAS version 9.4 (SAS Institute, Cary, NC, USA). At baseline, analysis of variance (ANOVA) for continuous variables and a Chi-square test for categorical variables were performed for comparisons of the demographic and clinical characteristics of the treatment group. For lipidaemia analyses, the overall differences among treatment groups were compared with the use of a global test of unordered groups. If the difference was significant at a *P* value of <0.05, then pairwise comparisons were made with adjustment for multiple comparisons, statistical significance was defined as adjusted *P* < .05 after adjustment for multiple comparisons with Tukey correction, and ANCOVA was also used with stratified randomization factors as a covariate. The 95% confidence interval (CI) was constructed with the use of least-squares (LS) means. We also performed a sensitivity analysis for participants without hypolipidemic medication (Table S6).

Statistical analyses of metagenomic and metabolomic data were performed using R software (version 3.6.3; R Foundation for Statistical Computing). Kruskal–Wallis (KW) tests were applied to detect differences in the lipid metabolites among the four groups at baseline. Canonical analysis of principal coordinates (CAP) based on Bray–Curtis distance was conducted using alterations of lipid metabolites after treatment to determine the differences among the four treatment groups (capscale function, vegan package). Wilcoxon signed-rank tests were applied to detect differences in lipid metabolite levels between baseline and post-treatment measurements for each treatment group.

Partial least squares-discriminant analysis (PLS-DA) was performed to construct the variable importance of projection (VIP) scores. VIP scores ≥1 were considered significant for lipid metabolites altered by treatment (plsda and vip function, mixOmics package).^52^ Multivariate generalized estimating equation (GEE) model analysis^53,54^ was performed to assess the longitudinal associations between the two time-point measurements of lipid metabolites and clinical parameters or species abundances after adjustment for age, sex and BMI (geem function, geeM package), and the *P* value of each regression coefficient (β) was calculated. The Wilcoxon matched-pairs signed-rank tests were applied to detect differences in RA of gut microbial species between baseline and posttreatment measurements. The correlations between the RAs of microbial species and clinical parameters in the Prob+BBR treatment groups were assessed by partial Spearman’s correlation analysis after adjustment for age, sex and BMI (pcor function, ppcor package). The differences in RA of microbial species between participants with dyslipidemia and lipidaemia at baseline were assessed by using KW tests. The adjusted *P*-value (q) was calculated with Benjamini–Hochberg (BH) method to correct the multiple comparisons and correlations of the lipid metabolite levels and gut microbial species (p.adjust function, stats package). Two-way analysis of variance (ANOVA) and unpaired Student’s t-test were used to compare the growth curves of the *in vitro* culture experiment.

## Results

### Prob+BBR combined treatment significantly improves PL

Our previous study has shown that BBR and Prob+BBR exert similar effects in reducing fasting lipidemia,^33^ we thus sought to investigate how the two treatments affect PL. The baseline characteristics of postprandial plasma samples from 365 T2D participants in the PREMOTE study (NCT02861261), showed no significant difference in PL among the four treatment groups (Table 1). At the end of the follow-up period, participants in the Prob+BBR group had a greater reduction in pTC and pLDLc from baseline to week 13 than those in the Plac group (LS mean 95% [CI], −24.29 [−29.95, −18.64] vs −8.66 [−14.52, −2.79] mg/dl, *P* = .001 in pTC, and −16.54 [−21.30, −11.79] vs −7.35 [−12.29, −2.42] mg/dl, *P* = .043 in pLDLc, respectively, ANOVA, Table 2, Table S4). However, compared to the Plac group, neither the BBR alone group (pTC, *P* = .14 vs Plac; pLDLc, *P* = .91 vs Plac) nor the Prob alone group (pTC, *P* = .33 vs Plac; pLDLc, *P* = .34 vs Plac) showed significant changes in postprandial cholesterols. Furthermore, controlling for stratified randomization factors similar results were shown (Model 2, Table 2). No additional benefit in improving pTG was found in Prob+BBR group compared to BBR (Table 2, Table S5). Of note, the effects of Prob+BBR treatment in improving PL were still significant when those taking hypolipidemic medications were excluded (n = 360, see Methods, Table S6). Thus, our study demonstrated that BBR alone was effective in reducing fasting levels but not in postprandial levels of cholesterols, the latter of which might require a synergistic effect with probiotics.

**Table 1.** Baseline characteristics of participants (n = 365)

	Plac (n = 91)		Prob (n = 92)	BBR (n = 84)	Prob+BBR (n = 98)		P value
Age, y	52.56 ± 9.44		52.11 ± 8.74	52.07 ± 10.81	52.9 ± 9.1		0.92
Male sex (%)	53 (54.08)		58 (63.04)	51 (60.71)	52 (57.14)		0.61
Body weight, kg	72.04 ± 12.18		71.72 ± 11.45	71.62 ± 13.1	70.63 ± 11.15		0.86
Waist circumference, cm	91.9 ± 8.98		91.34 ± 8.35	90.65 ± 9.4	90.56 ± 8.23		0.7
Hip circumference, cm	98.63 ± 6.6		97.91 ± 6.28	97.58 ± 7.09	98.19 ± 5.95		0.74
Systolic blood pressure, mmHg	129.58 ± 14.72		127.9 ± 13.76	128.48 ± 14.6	125.71 ± 12.86		0.28
Diastolic blood pressure, mmHg	80.38 ± 9.16		79.65 ± 7.8	79.51 ± 9.1	78.69 ± 8.88		0.62
Body mass index, kg/m^2^	26.26 ± 3.42		25.47 ± 2.91	25.78 ± 3.36	25.46 ± 2.85		0.27
Fasting triglyceride (IQI), mg/dl	109.77 (82.33, 143.61)	113.53 (76.32, 181.01)		116.92 (84.77, 159.21)		124.44 (89.66, 177.82)	0.32
Fasting total cholesterol, mg/dl	199.37 ± 37.99		203.08 ± 40.94	192.32 ± 40.61	203.34 ± 37.27		0.21
Fasting LDL cholesterol, mg/dl	128.51 ± 32.91		132.24 ± 33.74	124.3 ± 34.21	131.36 ± 31.71		0.38
Fasting HDL cholesterol, mg/dl	47.64 ± 10.38		46.94 ± 10.41	47.05 ± 10.77	46.2 ± 8.89		0.81
Postprandial triglyceride (IQI), mg/dl	112.03 (77.82, 150.38)		113.53 (88.53, 169.55)	112.03 (90.23, 145.68)	119.55 (88.72, 158.08)		0.3
Postprandial total cholesterol, mg/dl	186.96 ± 36.1		187.17 ± 37.07	181.6 ± 39.02	189.35 ± 36.65		0.56
Postprandial LDL cholesterol, mg/dl	108.11 ± 30.79		103.93 ± 34.39	101.24 ± 32.49	108.66 ± 28.47		0.34
Postprandial HDL cholesterol, mg/dl	39.92 ± 8.68		38.03 ± 8.34	38.73 ± 7.99	38 ± 6.71		0.32

Data are presented as the mean ± SD unless otherwise indicated. IQI, interquartile intervals.

Body mass index (BMI) is the weight in kilograms divided by the square of the height in meters.

The postprandial samples were drawn 120 min after taking 100 g standard noodle (a polysaccharide) provided by China Food Limited, COFCO (Beijing, China).^45^

**Table 2.** Changes in postprandial lipidaemia after treatment

		Model 1			Model 2		
		LS mean (95% CI)			LS mean (95% CI)		
		Change from Baseline	Treatment Difference	Adjusted *P* value	Change from Baseline	Treatment Difference	Adjusted *P* value
pTC (mg/dl)	Plac (91)	−8.66 (−14.52, −2.79)	Reference		−8.65 (−14.52, −2.78)	Reference	
	Prob (92)	−1.55 (−7.39, 4.28)	7.10 (−3.76, 17.96)	0.33	−1.52 (−7.37, 4.32)	7.13 (−3.75, 18)	0.33
	BBR (84)	−17.89 (−24.00, −11.79)	−9.24 (−20.35, 1.87)	0.14	−17.87 (−23.99, −11.76)	−9.22 (−20.35, 1.91)	0.14
	Prob+BBR (98)	−24.29 (−29.95, −18.64)	−15.64 (−26.33, −4.94)	0.001	−24.34 (−30.01, −18.67)	−15.69 (−26.41, −4.97)	0.001
pLDLc (mg/dl)	Plac (91)	−7.35 (−12.29, −2.42)	Reference		−7.36 (−12.3, −2.42)	Reference	
	Prob (92)	−1.43 (−6.34, 3.48)	5.93 (−3.21, −15.06)	0.34	−1.46 (−6.38, 3.45)	5.9 (−3.25, 15.04)	0.34
	BBR (84)	−9.81 (−14.95, −4.67)	−2.46 (−11.81, 6.90)	0.91	−9.84 (−14.98, −4.69)	−2.48 (−11.84, 6.89)	0.90
	Prob+BBR (98)	−16.54 (−21.30, −11.79)	−9.19 (−18.19, −0.19)	0.043	−16.48 (−21.25, −11.7)	−9.12 (−18.14, −0.1)	0.046
pTG (mg/dl, log)	Plac (91)	−0.03 (−0.10, 0.04)	Reference		−0.03 (−0.1, 0.04)	Reference	
	Prob (92)	−0.05 (−0.12, 0.02)	−0.02 (−0.16, 0.12)	0.98	−0.05 (−0.12, 0.03)	−0.02 (−0.16, 0.12)	0.98
	BBR (84)	−0.18 (−0.26, −0.10)	−0.15 (−0.29, −0.01)	0.037	−0.18 (−0.25, −0.1)	−0.15 (−0.29, −0.01)	0.038
	Prob+BBR (98)	−0.17 (−0.24, −0.10)	−0.14 (−0.28, −0.01)	0.035	−0.18 (−0.25, −0.1)	−0.14 (−0.28, −0.01)	0.031
pHDLc (mg/dl)	Plac (91)	0.51 (−0.53, 1.56)	Reference		0.51 (−0.54, 1.55)	Reference	
	Prob (92)	0.55 (−0.49, 1.59)	0.04 (−1.90, 1.97)	1.00	0.53 (−0.51, 1.57)	0.02 (−1.92, 1.95)	1
	BBR (84)	1.42 (0.34, 2.51)	0.91 (−1.07, 2.89)	0.64	1.41 (0.32, 2.5)	0.9 (−1.08, 2.88)	0.65
	Prob+BBR (98)	1.16 (0.15, 2.17)	0.65 (−1.26, 2.56)	0.82	1.2 (0.19, 2.21)	0.69 (−1.21, 2.6)	0.78

Model 1: Analysis of variance (ANOVA) was performed to compare the change in postprandial lipidaemia between groups using Tukey’s method for multiple pairwise comparisons.

Model 2: ANCOVA was performed to compare the postprandial change in lipidaemia between groups adjusted for prespecified age group using Tukey’s method for multiple pairwise comparisons. LS: least-squares means. All *P* values reported were two-sided, and statistical significance was defined as adjusted *P* < 0.05 after adjustment for multiple comparisons of Tukey correction. pTC: postprandial TC; pLDLc: postprandial LDL cholesterol; pTG (log): postprandial triglyceride.

### Prob+BBR treatment induces substantial changes in the postprandial lipidomic profile

**Figure 1.**
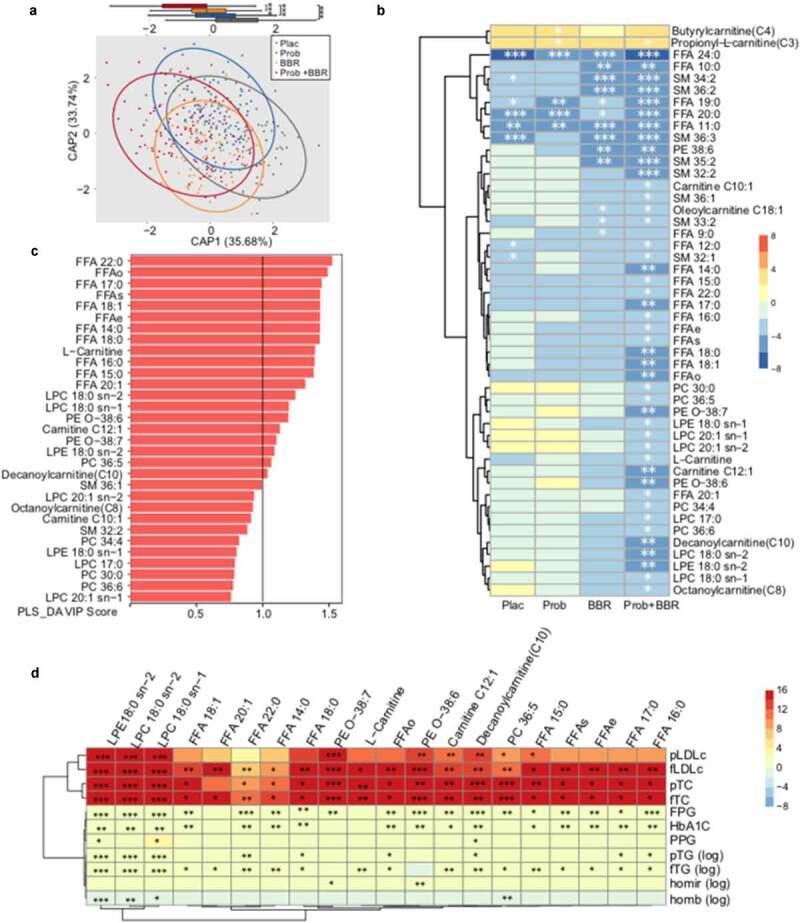
Lipidomic study on postprandial blood samples

To explore how lipid metabolism regulation might be affected by Prob+BBR, we further performed LC/MS-based lipidomic analysis on postprandial blood samples. The pre-treatment postprandial lipidomic composition between groups was similar (Data Set 1). CAP ordination analysis demonstrated that the treatment induced changes in lipidomic composition in the Prob+BBR group significantly differed from those in the Plac, Prob, and BBR groups (Figure 1a, *p* < .001 at CAP1). Thirty-one lipidomic metabolites were uniquely and significantly altered by the Prob+BBR treatment (Figure 1b, Wilcoxon signed-rank test, Data Set 2, q < 0.01). However, only eight in the BBR group and two in the Prob group of postprandial lipid metabolites were changed more than what were changed in the Plac group after follow-up. PLS-DA analysis showed that the 20 of the 31 postprandial lipid metabolites altered in the Prob+BBR group significantly contributed to the separation of baseline and post-treatment samples (VIP score > 1, Figure 1c, Figure S3), which we then designated as the key combined treatment responding lipid metabolites, including long to medium chain fatty acids (FFAs), acyl-carnitines and multiple glycerophospholipids: lysoglycerophosphatidylcholine (LPC), lysoglycerophatidylethanolamine (LPE), glycerophosphatidylcholine (PC), glycerophatidylethanolamine (PE) with alkyl and alkenyl substituents. The alterations in these key lipid metabolites were strongly associated with the improvement of fasting and/or the postprandial levels of LDLc and TC and, to a lesser extent, with those of TG and glycemia indices in the Prob+BBR group (Figure 1d, GEE, q < 0.05, Data Set 5). Thus, the decreases in multiple postprandial FFAs and phospholipids after Prob+BBR treatment might contribute to the overall reduced PL levels.

(a) Canonical analysis of principal coordinates (CAP) ordination plot (Bray-Curtis) of the changes in pre-post-treatment postprandial blood lipidomic metabolites in four treatment groups; with the two primary axes (CAP 1 and CAP 2) accounting for 69.42% of the total variance. The Tukey-style box plot shows the distribution of the first canonical axis (CAP 1) of lipid profiles. ***, *P* < .001. Samples beyond the end of the whiskers are called “outlying” points. Plac, placebo, n = 91; Prob, probiotic treatment, n = 92; BBR, berberine treatment, n = 84; Prob+BBR: berberine plus probiotic treatment, n = 98. (b) Heatmap of significant changes in postprandial lipidomic metabolites by four different treatments. The Z-score was calculated with the Wilcoxon signed-rank test. A Z-score >0 (red) indicated an increase after treatment, and a Z-score <0 (blue) indicated a decrease after treatment. *, q < 0.01, **, q < 0.001, ***, q < 0.0001. FFA: fatty acid; FFAo: the sum of odd-carbon-chain fatty acids; FFAe: the sum of even-carbon-chain fatty acids; FFAs: the sum of all fatty acids; LPC: lysoglycerophosphatidylethanolamine; LPE: lysoglycerophartylethanolamine; PC: glycerophosphosphatidylcholine; PE: glycerophatidyethanolamine; SM: sphingomyelin; PE O-:PE with alkyl and alkenyl substituents. (c) Bar plots showing the variable importance in projection (VIP) scores of postprandial lipidomic metabolites from the partial least squares-discriminant analysis (PLS-DA) in the Prob+BBR group. A total of 31 metabolites that were uniquely and significantly altered in the Prob+BBR group (see panel B) are presented. (d) Heatmap of longitudinal associations between the changes in twenty important lipidomic metabolites (VIP score >1, see panel B) and clinical characteristics response to Prob+BBR treatment. Multivariate Generalized Estimating Equation (GEE) analysis, controlling for age, sex and BMI. The color key represents the β value from GEE models. *, q < 0.05, **, q < 0.01, ***, q < 0.001. fTC: the fasting level of total cholesterol; fLDLc: low-density lipoprotein cholesterol; fTG (log): log of the fasting level of triglyceride; pTC: postprandial TC; pLDLc: postprandial LDL cholesterol; pTG (log): log of postprandial triglyceride; HbA1c: glycated hemoglobin; homair (log): log of (fasting serum insulin * fasting plasma glucose)/22.5, homeostasis model assessment index for assessing insulin resistance; homab (log): log of (20 * fasting serum insulin)/(fasting plasma glucose – 3.5), homeostasis model assessment index for assessing ß cell function; Prob+BBR: berberine plus probiotics treatment. n = 98.

### Recovering fecal enrichment of *B. breve* could be responsible for Prob+BBR induced PL improvement

**Figure 2.**
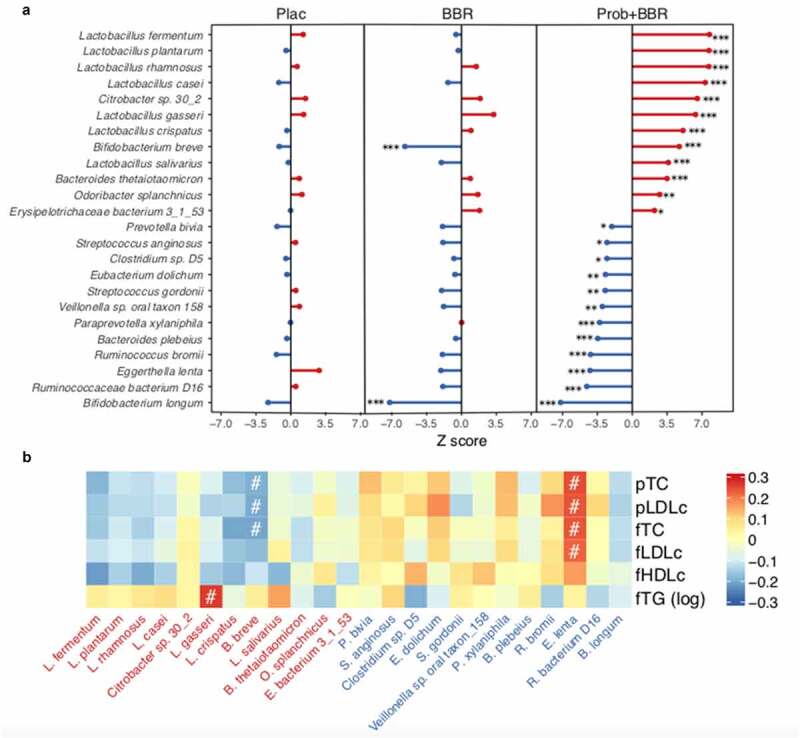
Gut microbial species correlate with blood lipidemia profiles

**Figure 3.**
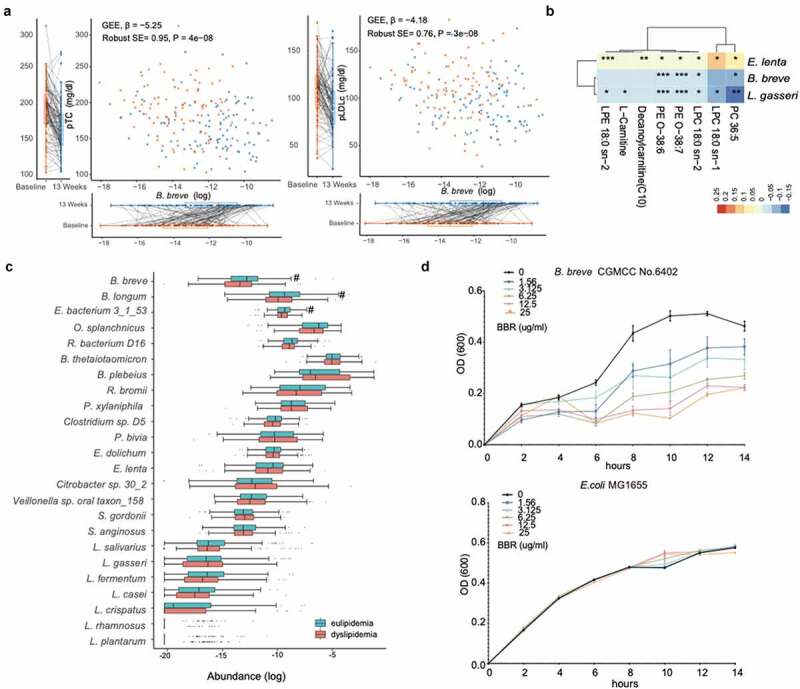
*B. breve* correlates with lipid metabolites changes and is depleted by BBR

As shown in our previous study,^33^ we found significant changes in gut microbiota in the Prob+BBR group. We then sought to ask whether the gut microbial alterations could underlie the benefit of Prob+BBR on PL compared to BBR alone. Twenty-four species in the gut microbiome were found to only respond to Prob+BBR treatment, including nine ingested probiotic strains^33^ (Figure 2a, Wilcoxon signed-rank test, q < 0.05). *B. breve* was the only taxon that was significantly reduced by BBR and increased in the Prob+BBR group. RAs of *B. breve* and *Eggerthella lenta* were correlated with the level of PL after Prob+BBR treatment (Figure 2b, Spearman, *P* < .05, Data Set 6). Higher fecal levels of *B. breve* and lower levels of *E. lenta* after treatment were associated with better control of PL in Prob+BBR group. Multivariate GEE analysis further suggested that increment of *B. breve* after treatment were significantly associated with the reductions of pTC (Figure 3a, β [SE] – 5.25 [0.95], *P* = 4.00 E-08) and pLDLc (Figure 3a, β [SE] – 4.18 [0.76], *P* = 3.00 E-08). In addition, GEE analysis of alterations in lipid metabolites and microbial taxa relating with PL improvements (listed in Figure 2b) showed that the changes in RAs of probiotic species including *B. breve* were negatively correlated with the changes in carnitines and phospholipids such as LPC 18:0 sn-2, PE-O 38:7 and PE O-38:6 (Figure 3b, q < 0.05, Data Set 7). These metabolites were also positively correlated with PL changes (Figure 1d). Furthermore, we found that baseline fecal RAs of *B. breve, B. longum* and the genus *Bifidobacterium* were significantly lower in participants with dyslipidemia than those with eulipidemia (Figure 3c, KW, *P* < .05, Data Set 8). Hence, considering the different treatment induced changes of *B. breve* between BBR alone and Prob+BBR groups, its correlations with PL and lipidomic metabolites, and its enrichment in participants with eulipidemia, *B. breve* might be an effective component in the probiotic formula to improve PL with BBR. In *in vitro* culture, *B. breve* growth was significantly inhibited by BBR in a dose-dependent manner, whereas the growth of *E. coli* was not affected by BBR even at the highest concentration applied for *B. breve* (Figure 3d). These results further consolidated our hypothesis that the superior effects of the combined treatment in improving PL and postprandial lipidomic profile might be related to recovering the reduced biomass of *B. breve* with BBR treatment.

(a) The changes in RAs of 24 selected microbial species uniquely altered in the Prob+BBR (right) compared to the Plac group (left), including 15 species altered uniquely and significantly by Prob+BBR and the 9 species from the probiotic mixture. Z-scores were calculated with the two-sided Wilcoxon signed rank tests. A Z-score >0 (red) indicated an increase after treatment and a Z-score <0 (blue) indicated a decrease after treatment. *, q < 0.05, **, q < 0.01, ***, q < 0.001. Plac, Placebo, n = 91; BBR: berberine alone treatment, n = 84; Prob+BBR: berberine plus probiotic treatment, n = 98. (b) Correlations between post-treatment abundances of species in (A) and serum levels of cholesterol and triglycerides in the Prob+BBR group. Spearman correlation, ^#^, q < 0.1. The color key represents the rho value. Red and blue represent the enriched and depleted species (ranked as same as panel A) after Prob+BBR treatment, respectively.

(a) Scatter plot of the levels of postprandial total cholesterol (pTC, left panel) and LDL cholesterol (pLDLc, right panel) levels against the relative abundances of *B. breve* in each subject at baseline and after 13 weeks treatment in the Prob+BBR group. β value and *P*-value for their longitudinal associations are calculated by GEE model. Tukey-style box plots showing the levels of pTC (left), pLDLc (right), and relative abundances of *B. breve* (at the bottom of the scatter plot) in the corresponding subjects, respectively (orange: baseline, n = 98; blue: 13 weeks, n = 98). (b) Heatmap of longitudinal associations between the changes in important lipidomic metabolites (VIP score >1, Figure 1b) and microbial species response to Prob+BBR treatment. Multivariate GEE analysis, controlling for age, sex, and BMI. The color key represents the β value from GEE models. *, q < 0.05, **, q < 0.01, ***, q < 0.001. Only three species significantly correlated with blood lipid profiles (Figure 2b) are shown in the panel. (c) Tukey-style bar plot showing the differences in RAs of 24 microbial species between participants with dyslipidaemia (n = 171) and eulipidaemia (n = 194) at baseline. Only the nine probiotic species and those uniquely responses to the Prob+BBR treatment (Figure 2a) are shown. Kruskal–Wallis tests, #, q < 0.1. (d) The growth curve of *B. breve* CGMCC No. 6402 (left) and *E. coli* MG1655 (right) with different concentrations of BBR (0–25 ug/mL) in the *in vitro* culture experiment. n = 4, *P* < .001, determined by two-way repeated measures ANOVA. Data are shown as the mean ± SE.

### *BBR induces the expression of genes regulating FFA simulation in* B. breve

**Figure 4.**
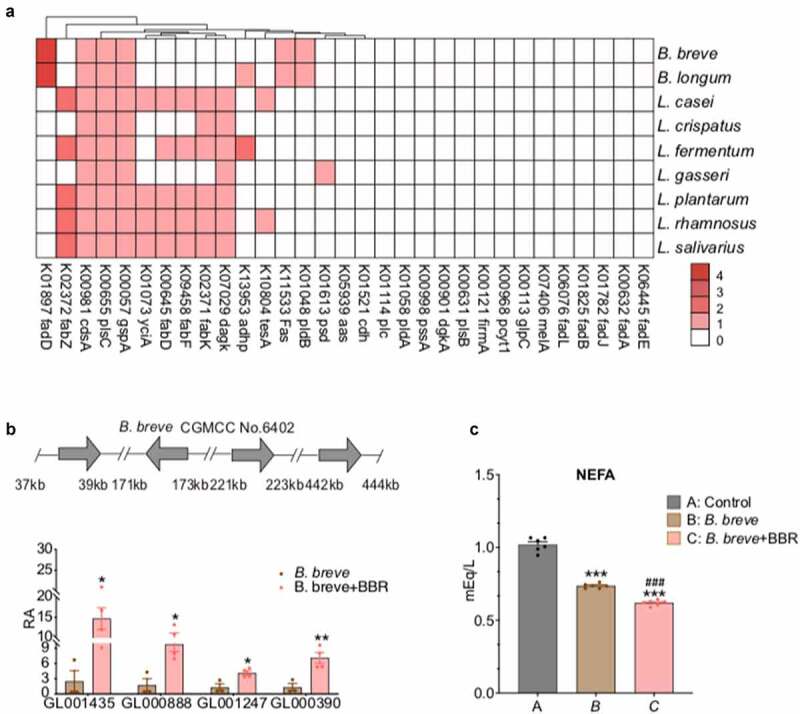
BBR activates lipid metabolism in *B. breve.*

We then asked whether there were factors other than recovering the decreased intraluminal biomass of *B. breve* after BBR treatment could improve PL lowering effect in Prob+ BBR. KEGG functional analysis of the fecal sample showed that Prob+BBR exhibited higher fatty acid metabolism and lower fatty acid synthesis potential than BBR alone treatment group (Figure S4). Using the draft genomes of the 9 probiotic strains in our formula^33^ and 1,520 high-quality genomes from cultivated human gut bacteria,^51^ we illustrated the distribution of genes regulating bacterial lipid metabolism in all 24 species responding to Prob+BBR treatment (Data Set 4, Figure 4a). Notably, the 9 probiotic strains all contained phospholipid biosynthesis genes including *gspA* (glycerol-3-phosphate dehydrogenase (NAD(P)+), K00057), *plsC* (1-acyl-sn-glycerol-3-phosphate acyltransferase, K00655) and *cdsA* (phosphatide cytidylyltransferase, K00981). However, only two *Bifidobacterium* strains exclusively expressed *fadD* (long-chain acyl-CoA synthetase, K01897, EC 6.2.1.3 (Figure 4a), a key bacterial enzyme that involved in the import and mobilization of exogenous FFA.^55^ Four *fadD* genes were annotated in the *B. breve* and *B. longum* genomes, indicating that both strains might possess more active FFA import and mobilization capacity compared to those have none (Data Set 4). Interestingly, when *B. breve* was cultured *in vitro* with BBR, the RNA expression of all its four *fadD* genes was significantly elevated compared to control medium with vehicle (Figure 4b). Consistently, the non-esterified FFA levels in culture media with adding Linolenic acid at final concentration of 1 mg ml^−1^, were significantly reduced by *B. breve* and could be enhanced by BBR treatment (Figure 4c). Thus, in addition to maintaining intraluminal abundancy of *B. breve* via supplementing probiotics formula with BBR treatment, activating the capacities of *B. breve* lipid simulation by BBR also might contribute to the synergetic hypolipidemic benefit of the combined treatment.

(a) The distribution of genes encoding enzymes regulating bacterial glycerophospholipid metabolism, fatty acid biosynthesis and degradation pathways predicted in genomes of the nine bacterial strains in probiotics. Color key indicates gene numbers under KO in an individual strain.(b) The expression of *fadD* increased in *B. breve* after treatment with BBR (6.25 μg ml^−1^). The mRNA levels of the four *fadD* genes in *B. breve* were determined by quantitative PCR. n = 4, *, *P* < .05, **, *P* < .01 compared to vehicle, determined by unpaired two-tailed student’s t test.(c) *In vitro* FFA consumption of *B. breve*, non-esterified fatty acids (NEFAs) were measured in the medium supplemented with LA. n = 6, ***, *P* < .001 relative to control, ^###^, *P* < .001 relative to *B. breve* after treatment with BBR, unpaired two-tailed student’s t test. BBR, berberine; *fadD*, long-chain acyl-CoA synthetase, LA, linolenic acid.

## Discussion

This study based on a randomized clinical trial confirmed that Prob+BBR combined therapy exerted a similar effect on reducing fasting lipidaemia with BBR alone but a superior effect on the levels of pTC and pLDLc compared to either BBR or probiotics alone. A pseudo-target lipidomic study revealed a substantial decrease in various lipid species after Prob+BBR treatment, implying decreased intestinal lipid uptake. Further we found that the hypolipidemic effect of combined treatment could be gained from the recovery of BBR-induced *B. breve* depletion by ingested probiotics and the induction of microbial lipid import and mobilization by BBR. Thus, our study provided both clinical and experimental evidence to support the synergistic effect of supplemental empirical probiotics containing *Bifidobacteria* such as *B. breve* with BBR in lowering PL, which could serve as an effective remedy for managing T2D PL and general dyslipidemia with its effect in lowering FL.

Hyperglycemia and hyperlipidemia commonly coexist in patients with T2D, and both are main risk factors for ASCVDs events.^56^ Statins, the current main hypolipidemia medication to reduce major adverse cardiovascular events (MACEs) in T2D patients,^3^ exhibits unfavorable effects, such as increasing intestinal cholesterol absorption, blood glucose level and diabetes incidence, as well as the hepatoxicity and myotoxicity. These side effects (SE) have brought concerns to the clinical practice ,^57,58^ particularly in East Asia population, including the Chinese, who is more susceptible to the SEs of statins.^59,60^ A cross-sectional survey in a nationally representative sample of 15,540 Chinese adults reports only 3.5% and 3.4% of men and women with a total cholesterol ≥200 mg/dL has been treated with any antilipidemic medicine.^61^ Thus, our regimen of combined therapy with Prob+BBR, targeting the PL with antidiabetic effect, could provide an alternative treatment for managing hyperlipidemia in patients with T2D, particularly those who are intolerant to statins.

In most studies on the role of BBR in lipid metabolism regulation,^29–33,62,63^ only fasting lipid levels have been evaluated. Here, we first reported that BBR was less potent in lowering the PL in participants with T2D compared to its effect in lowering fasting lipidaemia. We attributed this diverse effect partly to the suppression of commensal gut *Bifidobacteria* by BBR, either in our data (Table S7) or in previous study.^64^ Supplementation with a probiotic strain of *B. breve* recovered the loss of its indigenous counterpart and significantly improved PL in BBR-treated participants with T2D. Apparently, the BBR induced gut microbiota alterations might not all be beneficial to host metabolism. The negative effect of BBR on *Bifidobacterium* taxa might compromise its effect in reducing PL and raised caution that the effect of the gut microbiome should not be neglected when developing new treatment strategies for metabolic diseases.

The effect of *Bifidobacterium* on lowering blood lipids, particularly cholesterol levels, has been well recognized and made it recognized as a nature hypolipidemic agent like BBR,^37–43^ albeit the underlying mechanism has not yet been clarified. In our study, we found that *B. breve* and *B. longum* as well as the genus *Bifidobacterium* were enriched in T2D participants with better lipidemia. Further we found the enrichment of *fadD* genes might mediate the distinguish lipid lowering effect of *B. breve. FadD* is a fatty acyl-CoA synthetase that facilitates bacterial exogenous FFA uptake,^55,65^ mobilizes medium-chain and long-chain fatty acids (for FFA elongation, degradation, phospholipid biosynthesis,^66,67^ and represses expression of genes for FFA biosynthesis.^55,68^ The significantly enhanced transcription of all 4 *fadD* genes and FFA consumption in *B. breve* by BBR could underlie the effect of Prob+BBR in reducing host intestinal lipid uptake and blood cholesterol levels. The activated gut microbial lipid metabolism brought by the combined treatment could hijack host intraluminal lipids so as to significantly improve postprandial lipidomic profile. This might explain why Prob alone was neutral in lowering lipidemia and became effective only when it was combined with BBR for treating T2D patients. Thus, we thought it could be necessary to supplement with probiotics containing *Bifidobacterium spp*. strains, when BBR is clinically used as a hypolipidemic agent.

However, though both *B. breve* and *B. longum* were supplemented in the probiotic formula and both contained multiple *fadD* genes, metagenomics analysis showed that only *B.breve* was significantly recovered in Prob+BBR group^33^ and only its RA alterations was correlated with those in pTC and pLDLc. It might be explained that the amount of *B.longum* applied in this study was less than what was required. In addition, we also found that some commensal gut species other than those were supplemented by probiotics, such as *E. lenta*, altered uniquely after Prob+BBR treatment, and positively correlated either with PLs or postprandial lipid metabolites (Figure 1d, Figure 3b), indicating its relevance to the effect of Prob+BBR on lowering PL. *E. lenta* has been reported to decrease in fecal samples from T2D subjects treated with Acarbose, in which multiple *Bifidobacterium* and *Lactobacillus* are elevated.^69^ Future work is warranted to evaluate if *E. lenta* could elevate the PL autonomously to serve as a potential new target for treating postprandial dyslipidemia, or just be a marker of lactic bacteria flourish in gut.

Our study has several limitations. The predesigned 13-week multicenter randomized, double-blind, placebo-controlled study can avoid bias and obtain powerful evidence but does not allow for the assessment of long-term efficacy on MACEs in the combined treatment of Prob+BBR. The PL were tested after a 100 g carbon meal with post-load blood glucose and might be constituted by hepatobiliary secretion or transintestinal cholesterol excretions.^70,71^ How the PL responses to mix or fat meal could be affected by the combined treatment requires further study. Considering that the gut microbiota could directly stimulate atherosclerosis via bacterial-host co-metabolism of the choline/betaine diet to produce TMA,^72–74^ the potential of our remedy to target this metabolic pathway will be of interest for future investigations. In addition, the findings derived from this investigation may not be generalized to those on statin therapy populations without caution.

In conclusion, this clinical trial-based study proved the therapeutic effect of a combined treatment of oral administration of probiotics with berberine on improving PL in patients newly diagnosed with T2D and proposed a new gut microbiome related remedy for managing dyslipidemia, covering both PL and FL, in patients with T2D.

## Supplementary Material

Supplemental Material

## Data statement

The data that support the findings of this study are available in https://www.ncbi.nlm.nih.gov/bioproject/PRJNA643353, reference number PRJNA643353, and within the article and its supplementary materials.

## Abbreviations

Nonstandard Abbreviations and Acronyms
ASCVD: Atherosclerotic cardiovascular disease
ANOVA: Analysis of variance
BBR: Berberine
*B. breve*: *Bifidobacterium breve*
BH: Benjamini–Hochberg
CAP: Canonical analysis of principal coordinates analysis
FFA: Fatty acid
FL: Fasting lipidemia
fLDLc: Fasting levels of low-density lipoprotein cholesterol
fTC: Fasting levels of total cholesterol
fTG: Fasting levels of triglycerides
GEE: Generalized estimating equation
HPLC: High-performance liquid chromatography
KEGG: Kyoto Encyclopedia of Genes and Genomes
KO: KEGG Orthology
KW: Kruskal–Wallis
LC-MS: Liquid chromatography–mass spectrometry
LPC: Lyso-glycerophosphatidylcholine
LPE: Lyso-glycerophatidylethanolamine
LS: Least-squares
MACE: Major adverse cardiovascular events
NCEP/ATP III National Cholesterol Education Program/Adult Treatment Panel III
NPC1L1: Niemann-Pick C1-like 1
nFL: Non-fasting lipidaemia
PC: Glycerophosphatidylcholine
PE: Glycerophatidylethanolamine
pHDLc: Postprandial high-density lipoprotein cholesterol
pLDLc: Postprandial low-density lipoprotein cholesterol
PLS-DA: Partial least squares-discriminant analysis
Plac: Placebo
Prob: Probiotics
Prob+BBR: Probiotics combined with Berberine
PREMOTE: Probiotics and BBR on the Efficacy and Change of Gut Microbiota in Patients with Newly Diagnosed Type 2 Diabetes trial.
pTC: Postprandial plasma total cholesterol
pTG: Postprandial plasma triglycerides
QC: Quality control
RA: Relative abundancy
RSD: Relative standard deviation
SM: Sphingomyelin
VIP: Variable importance in projection
