# Supplementary material for: Combined berberine and probiotic treatment as an effective regimen for improving postprandial hyperlipidemia in type 2 diabetes patients: a double blinded placebo controlled randomized study
Source: Gut Microbes. 2021 Dec 20;14(1):2003176. doi: 10.1080/19490976.2021.2003176 (PMC8726654; doi:10.1080/19490976.2021.2003176)
Supplement: Supplemental Material [file KGMI_A_2003176_SM5262.zip › Supplementary information/1005 manu sup.docx]

**SUPPLEMENTAL MATERIAL**

Combined berberine and probiotic treatment as an effective regimen for improving postprandial hyperlipidemia in type 2 diabetes patients: a double blinded placebo controlled randomized study

**Authors:** Shujie Wang, MD, PhD;^1^† Huahui Ren, PhD;^2^† Huanzi Zhong, PhD;^2^† Xinjie Zhao, PhD;^3^ Changkun Li, PhD;^1^ Jing Ma, MD, PhD;^4^, Xuejiang Gu, MD, PhD;^5^ Yaoming Xue, MD, PhD;^6^ Shan Huang, MD, PhD;^7^ Jialin Yang, MD, PhD;^8^ Li Chen, MD, PhD;^9^ Gang Chen, MD, PhD;^10^ Shen Qu, MD, PhD;^11^ Jun Liang, MD, PhD;^12^ Li Qin, MD, PhD;^13^ Qin Huang, MD, PhD;^14^ Yongde Peng, MD, PhD;^15^ Qi Li PHD;^3^ Xiaolin Wang, PhD;^3^ Yuanqiang Zou, PhD;^2^ Zhun Shi, PhD;^2^ Xuelin Li, MD;^1^ Tingting Li, MD, PhD;^1^ Huanming Yang, PhD;^2, 16^ Shenghan Lai, PhD;^17^ Guowang Xu, PhD;^3^ Junhua Li, PhD;^2,18^* Yifei Zhang, MD, PhD;^1^* Yanyun Gu, MD, PhD; ^1^* Weiqing Wang, MD, PhD^1^*

^1^ Department of Endocrine and Metabolic Diseases, Shanghai Institute of Endocrine and Metabolic Diseases, Ruijin Hospital, Shanghai Jiao Tong University School of Medicine, Shanghai, China; Shanghai National Clinical Research Center for metabolic Diseases, Key Laboratory for Endocrine and Metabolic Diseases of the National Health Commission of the PR China, Shanghai National Center for Translational Medicine, Ruijin Hospital, Shanghai Jiao Tong University School of Medicine, Shanghai, China; ^2^BGI-Shenzhen, Shenzhen, China; China National GeneBank, BGI-Shenzhen, Shenzhen, China; ^3^Dalian Institute of Chemical Physics, Chinese Academy of Science, Dalian, China; ^4^Ren Ji Hospital, Shanghai Jiao Tong University School of Medicine, Shanghai, China; ^5^The First Affiliated hospital of Wenzhou Medical University, Zhejiang Province, China; ^6^Nanfang Hospital, Southern Medical University, Guangdong Province, China; ^7^Tong Ren Hospital, Shanghai Jiao Tong University School of Medicine, Shanghai, China; ^8^Central Hospital of Minhang district, Shanghai, China; ^9^Qilu Hospital of Shandong University, Shandong Province, China; ^10^Fujian Provincial Hospital, Fujian Province, China; ^11^Shanghai Tenth People’s Hospital of Tong Ji University, Shanghai, China; ^12^Xuzhou Central Hospital, Jiangsu Province, China; ^13^Xin Hua Hospital, Shanghai Jiao Tong University School of Medicine, Shanghai, China; ^14^Chang Hai Hospital, Second Military Medical University, Shanghai, China; ^15^Shanghai First People’s Hospital, Shanghai Jiao Tong University School of Medicine, Shanghai, China; ^16^James D. Watson Institute of Genome Sciences, Hangzhou, China; ^17^Johns Hopkins University School of Medicine, Baltimore, Maryland. ^18^School of Biology and Biological Engineering, South China University of Technology, Guangzhou, China.

**Table S1**. A summary of the genome assembly statistics for each genome of the 9 ingested probiotics.

| **Sample Name** | **Seq Type (#)** | **Total Number (#)** | **Total Length (bp)** | **N50 Length (bp)** | **N90 Length (bp)** | **Max Length (bp)** | **Min Length (bp)** | **Gap Number (bp)** | **GC Content (%)** |
| --- | --- | --- | --- | --- | --- | --- | --- | --- | --- |
| *B. longum* | Scaffold | 23 | 2,151,613 | 198,056 | 87,009 | 352,265 | 726 | 0 | 60 |
|  | Contig | 23 | 2,151,613 | 198,056 | 87,009 | 352,265 | 726 | - | 60 |
| *B. breve* | Scaffold | 18 | 2,262,953 | 525,289 | 83,737 | 824,695 | 518 | 0 | 58.87 |
|  | Contig | 18 | 2,262,953 | 525,289 | 83,737 | 824,695 | 518 | - | 58.87 |
| *L. casei* | Scaffold | 170 | 2,813,692 | 40,829 | 8,539 | 126,804 | 501 | 319 | 47.71 |
|  | Contig | 171 | 2,813,373 | 40,829 | 8,539 | 126,804 | 501 | - | 47.71 |
| *L. crispatus* | Scaffold | 196 | 2,210,709 | 25,423 | 5,687 | 94,836 | 505 | 85 | 36.66 |
|  | Contig | 197 | 2,210,624 | 25,423 | 5,687 | 94,836 | 505 | - | 36.66 |
| *L. fermentum* | Scaffold | 133 | 2,034,449 | 39,034 | 8,607 | 165,854 | 500 | 554 | 51.74 |
|  | Contig | 134 | 2,033,895 | 39,034 | 9,275 | 165,854 | 500 | - | 51.74 |
| *L. gasseri* | Scaffold | 7 | 1,928,893 | 583,872 | 213,543 | 881,479 | 1,169 | 64 | 34.65 |
|  | Contig | 11 | 1,928,829 | 447,573 | 213,543 | 539,059 | 1,169 | - | 34.65 |
| *L. plantarum* | Scaffold | 26 | 3,238,409 | 334,366 | 74,946 | 625,233 | 566 | 241 | 44.46 |
|  | Contig | 27 | 3,238,168 | 334,366 | 74,946 | 625,233 | 566 | - | 44.46 |
| *L. rhamnosus* | Scaffold | 68 | 2,992,017 | 120,142 | 30,961 | 286,426 | 608 | 234 | 46.57 |
|  | Contig | 69 | 2,991,783 | 120,142 | 30,961 | 286,426 | 608 | - | 46.57 |
| *L. salivarius* | Scaffold | 27 | 1,999,796 | 124,742 | 41,295 | 419,305 | 599 | 235 | 32.53 |
|  | Contig | 28 | 1,999,561 | 124,742 | 41,295 | 419,305 | 599 | - | 32.53 |

**Table S2**. Culture media formula for *B. breve.*

| *B. breve* **Medium formula** | | |
| --- | --- | --- |
| **Component** | **Amount/L** | |
| Trypticase peptone | 5.00 g | |
| Peptone | 5.00 g | |
| Yeast extract | 10.00 g | |
| Glucose | 2.00 g | |
| Maltose | 10.00 g | |
| Cysteine-HCl x H_2_O | 0.50 g | |
| Na_2_CO_3_ | 2.5 g | |
| Salt solution (see below) | 40.00 ml | |
| Vitamin K1 solution (see below) | 0.20 ml | |
| Agar | 1 g | |
|  |  | |
| **Salt solution (DSMZ Salt solution):** | |  |
| CaCl_2_ x 2 H_2_O | 0.25 g | |
| MgSO_4_ x 7 H_2_O | 0.50 g | |
| K_2_HPO_4_ | 1.00 g | |
| KH_2_PO_4_ | 1.00 g | |
| NaHCO_3_ | 10.00 g | |
| NaCl | 2.00 g | |
| Distilled water | 1000.00 ml | |

**Table S3**. Primer list for real-time PCR.

| **Gene symbol** | **Forward primer** | **Reverse primer** |
| --- | --- | --- |
| GL000390 (fadD) | CGTATTTCCGATGAGGGCGA | GTCGCGTTCGCTTTATCCAC |
| GL000888 (fadD) | GTTTTGGCGGCACCATTTCT | GTGCCGATGCGATTGTCTTC |
| GL001247 (fadD) | ACTGCTTACCACGTCCACTG | CGAGTCGGTGTCGTAGATGG |
| GL001435 (fadD) | GTCAAGTGGAGCAAGGACGA | CATTGGCATTGGCCTGATCG |
| Bifidobacterium 16S | TCG CGT CYG GTG TGA AAG | CCA CAT CCA GCR TCC AC |

**Table** S4**. Non-fasting lipidaemia of Participants.**

|  |  | **Plac** | **Prob** | **BBR** | **Prob+BBR** |
| --- | --- | --- | --- | --- | --- |
| **Postprandial triglyceride (IQI), mg/dl** | **Pre** | 112.03 (77.82, 150.38) | 113.53 (88.53, 169.55) | 112.03 (90.23, 145.68) | 119.55 (88.72, 158.08) |
|  | **Post** | 109.02 (75.94, 142.86) | 121.43 (73.68, 167.29) | 97.74 (75.38, 133.46) | 97.36 (75.94, 129.32) |
| **Postprandial total cholesterol, mg/dl** | **Pre** | 186.96±36.1 | 187.17±37.07 | 181.6±39.02 | 189.35±36.65 |
|  | **post** | 178.30±35.01 | 185.62±34.78 | 163.70±36.65 | 165.05±33.05 |
| **Postprandial LDL cholesterol, mg/dl** | **Pre** | 108.11±30.79 | 103.93±34.39 | 101.24±32.49 | 108.66±28.47 |
|  | **Post** | 100.76±29.75 | 102.51±28.84 | 91.43±30.13 | 92.11±27.20 |
| **Postprandial HDL cholesterol, mg/dl** | **Pre** | 39.92±8.68 | 38.03±8.34 | 38.73±7.99 | 38±6.71 |
|  | **post** | 40.43±9.61 | 38.58±8.04 | 40.16±8.09 | 39.16 ±7.10 |

Data are presented as the mean±SD unless otherwise indicated. IQI, interquartile intervals.

Table S5. Changes in fasting lipidaemia after Prob+BBR treatment (n =365).

|  | fTC | | fLDLc | | fTG (log) | | | fHDLc | |
| --- | --- | --- | --- | --- | --- | --- | --- | --- | --- |
|  | Treatment Difference^&^ | P value^†^ | Treatment Difference^&^ | P value^†^ | | Treatment Difference^&^ | P value^†^ | Treatment Difference^&^ | P value^†^ |
| Plac (91) | Reference |  | Reference |  | | Reference |  | Reference |  |
| Prob (92) | 4.74 (-7.71, 17.18) | 0.76 | -3.47 (-13.55, 6.6) | 0.81 | | 0.09 (-0.07, 0.25) | 0.43 | -1.41 (-3.85, 1.04) | 0.45 |
| BBR (84) | -13.58 (-26.32, -0.85) | 0.03 | -12.13 (-22.45, -1.82) | 0.01 | | -0.17 (-0.33, -0.01) | 0.04 | -1.83 (-4.34, 0.67) | 0.23 |
| Prob+BBR (98) | -20.08 (-32.33, -7.82) | <0.001 | -15.39 (-25.32, -5.47) | <0.001 | | -0.19 (-0.35, -0.03) | 0.01 | -1.65 (-4.06, 0.76) | 0.29 |
| BBR | Reference |  | Reference |  | | Reference |  | Reference |  |
| Prob+BBR | -6.49 (-19.01, 6.02) | 0.54 | -3.26 (-13.4, 6.87) | 0.84 | | -0.02 (-0.18, 0.14) | 0.99 | 0.18 (-2.28, 2.64) | 1.00 |

&: Placebo subtracted change (95% CI), least-squares means. †P values refer to a comparison of change in fasting lipidaemia between groups using analysis of variance (ANOVA) with Tukey’s method for multiple pairwise comparisons. All P values reported are two-sided, and statistical significance was defined as adjusted P<0.05 after adjustment for multiple comparisons of Tukey correction. fTC: fasting total cholesterol; fLDL: fasting LDL cholesterol; fTG: fasting TG; fHDL: fasting HDL cholesterol.

**Table S6**. Changes in postprandial lipidaemia after treatment of 360 participants without recording antidyslipidaemia medication.

|  | | | | | **Model 1** | | | | **Model 2** | | | |
| --- | --- | --- | --- | --- | --- | --- | --- | --- | --- | --- | --- | --- |
|  | |  | | | | **LS mean (95% CI)** | | **Adjusted P value** | | **LS mean (95% CI)** | | **Adjusted P value** |
|  | | | **Change from baseline** | | | | **Treatment Difference** | | | **Change from baseline** | **Treatment Difference** | |
| **pTC (mg/dl)** | Plac (89) | | | -7.71 (-13.59, -1.83) | | | Reference |  | | -7.71 (-13.6, -1.82) | Reference |  |
|  | Prob (92) | | | -1.55 (-7.33, 4.23) | | | 6.16 (-4.67, 16.98) | 0.45 | | -1.56 (-7.36, 4.23) | 6.15 (-4.69, 16.99) | 0.45 |
|  | BBR (83) | | | -17.87 (-23.96, -11.79) | | | -10.16 (-21.27, 0.94) | 0.09 | | -17.88 (-23.97, -11.78) | -10.17 (-21.29, 0.95) | 0.09 |
|  | Prob+BBR (96) | | | -23.97 (-29.63, -18.31) | | | -16.27 (-26.98, -5.56) | 0.0006 | | -23.96 (-29.64, -18.28) | -16.25 (-26.99, -5.52) | 0.0006 |
| **pLDLc (mg/dl)** | Plac (89) | | | -6.78 (-11.74, -1.81) | | | Reference |  | | -6.77 (-11.74, -1.81) | Reference |  |
|  | Prob (92) | | | -1.43 (-6.31, 3.45) | | | 5.35 (-3.79, 14.49) | 0.43 | | -1.5 (-6.39, 3.39) | 5.28 (-3.87, 14.43) | 0.45 |
|  | BBR (83) | | | -9.59 (-14.73, -4.45) | | | -2.82 (-12.19, 6.56) | 0.87 | | -9.63 (-14.78, -4.49) | -2.86 (-12.25, 6.53) | 0.86 |
|  | Prob+BBR (96) | | | -16.63 (-21.4, -11.85) | | | -9.85 (-18.89, -0.81) | 0.027 | | -16.53 (-21.32, -11.74) | -9.75 (-18.81, -0.7) | 0.029 |

Model 1: Analysis of variance (ANOVA) was performed to compare the change in postprandial lipidaemia between groups using Tukey’s method for multiple pairwise comparisons.

Model 2: Multivariate ANOVA was performed to compare the change in postprandial lipidaemia between groups adjusted for prespecified age group using Tukey’s method for multiple pairwise comparisons.

LS means, least-squares means. All P values reported were two-sided, and statistical significance was defined as adjusted P<0.05 after adjustment for multiple comparisons of Tukey correction. pTC: postprandial total cholesterol; pLDLc: postprandial LDL cholesterol.

**Table S7:** Comparison of baseline and after treatment RAs of genus Bifidobacterium and Bifidobacterium spp. between different groups

|  | **Placebo** | | | | | | **Probiotic** | | | | | |
| --- | --- | --- | --- | --- | --- | --- | --- | --- | --- | --- | --- | --- |
|  | **P-value** | **Occurrence** | | **Median RA** | | **Z-score** | **P-value** | **Occurrence** | | **Median RA** | | **Z-score** |
|  |  | **Pre** | **Post** | **Pre** | **Post** |  |  | **Pre** | **Post** | **Pre** | **Post** |  |
| **Species** |  |  |  |  |  |  |  |  |  |  |  |  |
| *Bifidobacterium_adolescentis* | 3.47E-02 | 94.12% | 83.53% | 5.12E-06 | 2.96E-07 | -2.11 | 6.30E-02 | 94.38% | 95.51% | 8.789E-06 | 1.4832E-06 | -1.86 |
| *Bifidobacterium_angulatum* | 3.15E-02 | 88.24% | 68.24% | 4.58E-07 | 1.68E-07 | -2.15 | 1.33E-02 | 89.89% | 87.64% | 8.196E-07 | 4.26E-07 | -2.48 |
| *Bifidobacterium_animalis* | 9.95E-01 | 98.82% | 96.47% | 1.24E-06 | 1.2E-06 | -0.01 | 6.57E-01 | 95.51% | 95.51% | 6.464E-07 | 6.8051E-07 | -0.44 |
| *Bifidobacterium_bifidum* | 5.44E-01 | 91.76% | 87.06% | 8.46E-07 | 2.04E-07 | -0.61 | 8.35E-02 | 95.51% | 92.13% | 8.805E-07 | 6.4685E-07 | -1.73 |
| *Bifidobacterium_breve* | 2.16E-01 | 98.82% | 100.00% | 1.83E-06 | 1.23E-06 | -1.24 | 3.63E-07 | 100.00% | 100.00% | 2.089E-06 | 9.532E-06 | 5.09 |
| *Bifidobacterium_catenulatum-Bpc* | 1.59E-02 | 100.00% | 100.00% | 3.52E-05 | 1.28E-05 | -2.41 | 2.79E-02 | 100.00% | 100.00% | 4.685E-05 | 3.3636E-05 | -2.20 |
| *Bifidobacterium_dentium* | 2.53E-01 | 85.88% | 83.53% | 1.5E-06 | 8.69E-07 | -1.14 | 8.03E-01 | 93.26% | 85.39% | 1.772E-06 | 1.717E-06 | -0.25 |
| *Bifidobacterium_longum* | 2.03E-02 | 100.00% | 100.00% | 4.91E-05 | 2.45E-05 | -2.32 | 1.32E-01 | 100.00% | 100.00% | 6.301E-05 | 5.135E-05 | -1.51 |
| **Genus** |  |  |  |  |  |  |  |  |  |  |  |  |
| *Bifidobacterium* | 1.90E-01 | 100.00% | 100.00% | 0.000193 | 0.000102 | 1.31 | 1.43E-01 | 100.00% | 100.00% | 0.0003184 | 0.00022197 | 1.47 |
|  | **Berberine** | | | | | | **Berberine+probiotic** | | | | | |
|  | **P-value** | **Occurrence** | | **Median RA** | | **Z-score** | **P-value** | **Occurrence** | | **Median RA** | | **Z-score** |
|  |  | **Pre** | **Post** | **Pre** | **Post** |  |  | **Pre** | **Post** | **Pre** | **Post** |  |
| **Species** |  |  |  |  |  |  |  |  |  |  |  |  |
| *Bifidobacterium_adolescentis* | 1.56E-07 | 98.70% | 68.83% | 6.04E-06 | 6.78E-08 | -5.25 | 7.58E-13 | 97.87% | 84.04% | 6.633E-06 | 8.3745E-08 | -7.17 |
| *Bifidobacterium_angulatum* | 6.29E-12 | 94.81% | 27.27% | 6.65E-07 | 0 | -6.87 | 3.64E-12 | 88.30% | 34.04% | 5.655E-07 | 0 | -6.95 |
| *Bifidobacterium_animalis* | 2.32E-02 | 97.40% | 92.21% | 1.07E-06 | 5.89E-07 | -2.27 | 2.77E-01 | 100.00% | 88.30% | 8.291E-07 | 6.5771E-07 | -1.09 |
| *Bifidobacterium_bifidum* | 1.03E-07 | 96.10% | 61.04% | 1.17E-06 | 3.64E-08 | -5.32 | 2.75E-07 | 96.81% | 75.53% | 1.054E-06 | 1.6455E-07 | -5.14 |
| *Bifidobacterium_breve* | 1.05E-07 | 100.00% | 98.70% | 2.57E-06 | 5.28E-07 | -5.32 | 4.10E-06 | 100.00% | 100.00% | 2.253E-06 | 1.1695E-05 | 4.61 |
| *Bifidobacterium_catenulatum-Bpc* | 1.28E-09 | 100.00% | 98.70% | 3.76E-05 | 4.18E-06 | -6.07 | 9.06E-13 | 100.00% | 100.00% | 5.07E-05 | 3.5062E-06 | -7.14 |
| *Bifidobacterium_dentium* | 2.47E-05 | 90.91% | 72.73% | 2.03E-06 | 9.96E-08 | -4.22 | 7.55E-11 | 89.36% | 72.34% | 2.123E-06 | 7.751E-08 | -6.51 |
| *Bifidobacterium_longum* | 1.11E-11 | 100.00% | 100.00% | 9.46E-05 | 7.04E-06 | -6.79 | 7.01E-12 | 100.00% | 100.00% | 7.345E-05 | 1.1236E-05 | -6.86 |
| **Genus** |  |  |  |  |  |  |  |  |  |  |  |  |
| *Bifidobacterium* | 2.12E-11 | 100.00% | 100.00% | 0.000476 | 2.77E-05 | -6.70 | 1.11E-11 | 100.00% | 100.00% | 0.0002516 | 4.4065E-05 | -6.79 |

The p.value and Z-score were calculated with the Wilcoxon signed rank test between dyslipidemia and eulipidemia groups. A Z-score >0 indicated an increase of RA of the species in participants with dyslipidemia, and a Z-score <0 indicated a decrease in participants with eulipidemia. B. catenulatum-Bpc: Bifidobacterium_catenulatum-Bifidobacterium_pseudocatenulatum_complex.


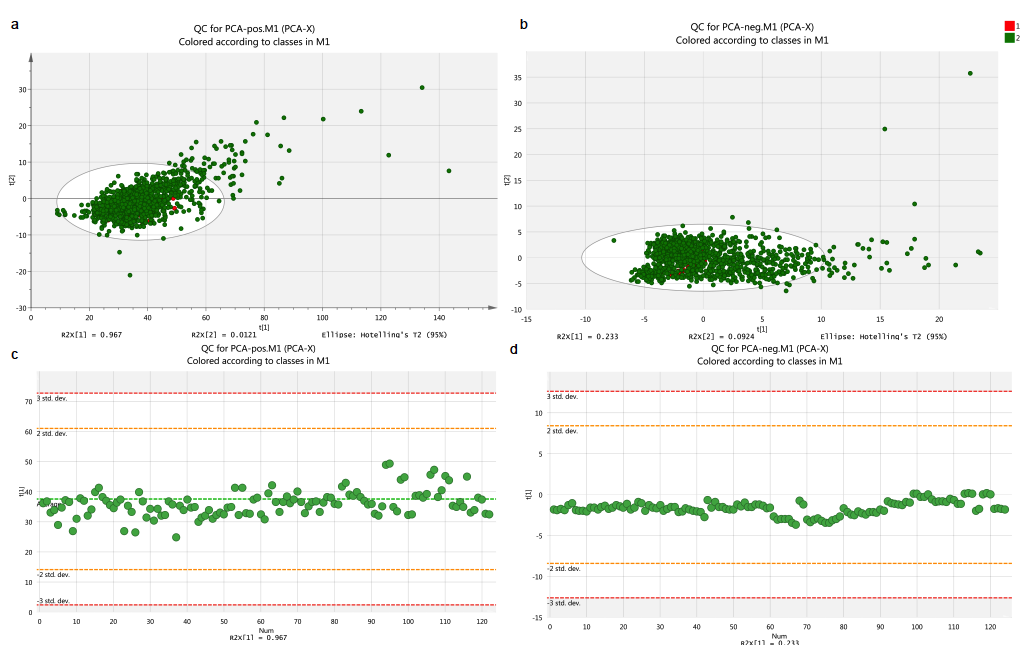


**Figure S1.** Quality control (QC) samples on the PCA score plot based on the first two principal components (a) in positive ion mode and (b) in negative ion mode; QC samples in the control chart along the first principal component (c) in positive ion mode and (d) in negative ion mode.

**Figure S2.** The distribution of relative standard deviation (RSD)% for all metabolites in QCs.

Columns represent the percentage of the peak number within the specified RSD% range. Lines show the cumulative percentages of the peak area within the specified RSD% range.

**
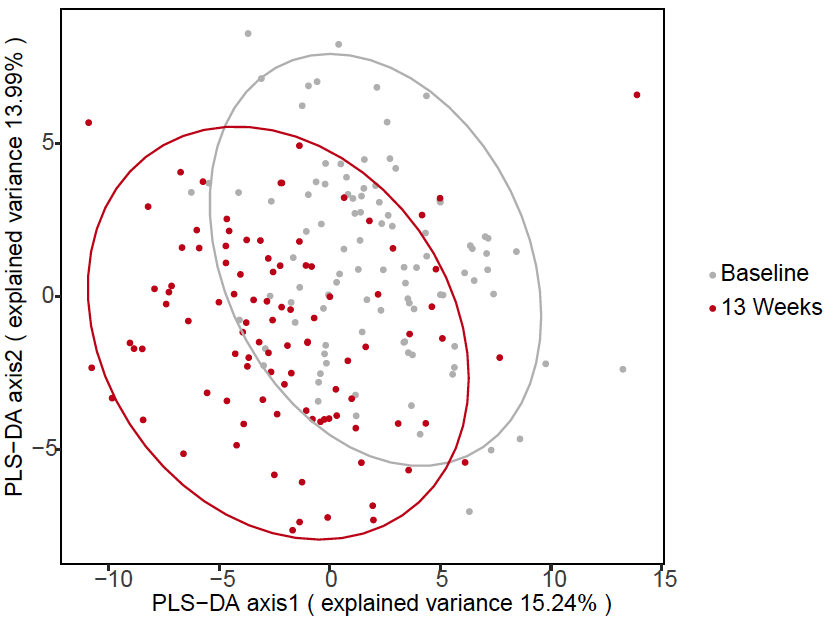
**

**Figure S3.** Partial least squares-discriminant analysis before and after treatment of the Prob+BBR group. Prob+BBR: berberine plus probiotic treatment (baseline, n = 98; 13 weeks, n = 98).

\


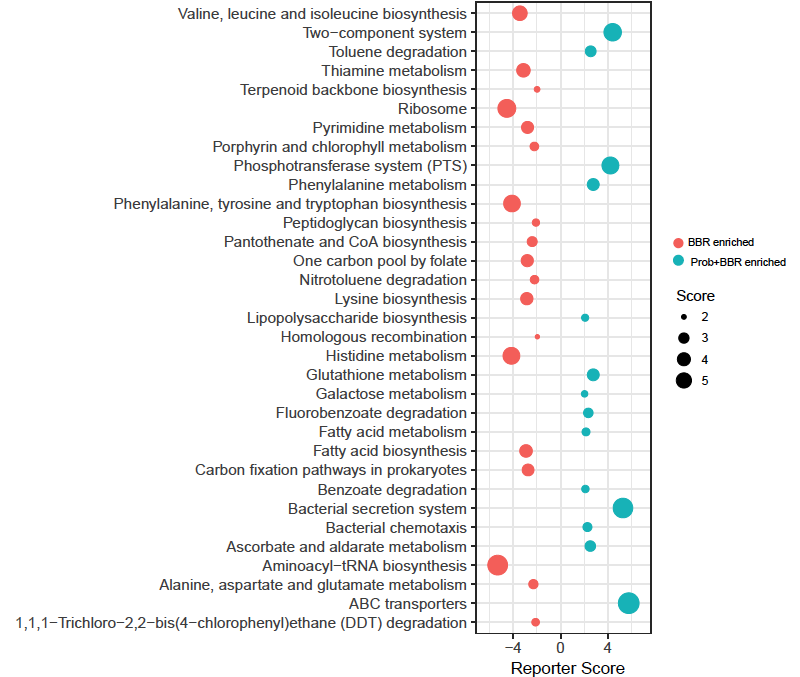


**Figure S4.** Differential enrichment of KEGG pathways in posttreatment individuals between the BBR and BBR+Prob groups. Pathways were ranked according to the reporter scores. An absolute value of reporter score ≥ 1.96 was shown and used as the detection threshold for significance. The size of the dot represents the absolute value of the Z score. The green colour represents the pathways enriched in the BBR group, and the red colour represents those enriched in the Prob+BBR. BBR treatment groups, n=84; Prob+BBR: berberine plus probiotic treatment, n=98.
