# Supplementary material for: Combined berberine and probiotic treatment as an effective regimen for improving postprandial hyperlipidemia in type 2 diabetes patients: a double blinded placebo controlled randomized study
Source: Gut Microbes. 2021 Dec 20;14(1):2003176. doi: 10.1080/19490976.2021.2003176 (PMC8726654; doi:10.1080/19490976.2021.2003176)
Supplement: Supplemental Material [file KGMI_A_2003176_SM5262.zip › Supplementary information/Data Set 1.docx]

**Data Set 1. Basal levels of plasma postprandial lipid species profile between groups**

|  | **Plac** | | | Prob | | | BBR | | | Prob+BBR | | | kw.p | P.adjust |
| --- | --- | --- | --- | --- | --- | --- | --- | --- | --- | --- | --- | --- | --- | --- |
|  | median_v1 | v1_0.25 | v1_0.75 | median_v1 | v1_0.25 | v1_0.75 | median_v1 | v1_0.25 | v1_0.75 | median_v1 | v1_0.25 | v1_0.75 |  |  |
| 2-Octenoylcarnitine(C8:1) | 0.18 | 0.13 | 0.26 | 0.17 | 0.10 | 0.23 | 0.17 | 0.11 | 0.25 | 0.16 | 0.11 | 0.26 | 0.55 | 0.89 |
| Butyrylcarnitine(C4) | 0.07 | 0.06 | 0.08 | 0.06 | 0.05 | 0.08 | 0.07 | 0.05 | 0.09 | 0.07 | 0.05 | 0.08 | 0.21 | 0.78 |
| Carnitine C10:1 | 0.15 | 0.11 | 0.20 | 0.14 | 0.11 | 0.20 | 0.13 | 0.10 | 0.19 | 0.12 | 0.09 | 0.18 | 0.20 | 0.78 |
| Carnitine C12:0 | 0.02 | 0.02 | 0.03 | 0.02 | 0.01 | 0.03 | 0.02 | 0.01 | 0.03 | 0.02 | 0.01 | 0.03 | 0.26 | 0.78 |
| Carnitine C12:1 | 0.03 | 0.02 | 0.04 | 0.02 | 0.02 | 0.04 | 0.03 | 0.02 | 0.04 | 0.03 | 0.02 | 0.04 | 0.42 | 0.89 |
| carnitine C12:2 | 0.01 | 0.01 | 0.01 | 0.01 | 0.01 | 0.01 | 0.01 | 0.01 | 0.01 | 0.01 | 0.01 | 0.01 | 0.37 | 0.88 |
| carnitine C14:3 | 0.00 | 0.00 | 0.00 | 0.00 | 0.00 | 0.00 | 0.00 | 0.00 | 0.00 | 0.00 | 0.00 | 0.00 | 0.73 | 0.97 |
| Decadienoylcarnitine C10:3 | 0.12 | 0.08 | 0.18 | 0.11 | 0.07 | 0.16 | 0.10 | 0.07 | 0.16 | 0.11 | 0.07 | 0.16 | 0.78 | 0.99 |
| Decanoylcarnitine(C10) | 0.10 | 0.07 | 0.13 | 0.09 | 0.07 | 0.14 | 0.09 | 0.06 | 0.12 | 0.09 | 0.06 | 0.13 | 0.54 | 0.89 |
| DecenoylcarnitineC10:2 | 0.01 | 0.01 | 0.02 | 0.01 | 0.01 | 0.02 | 0.01 | 0.01 | 0.02 | 0.01 | 0.01 | 0.02 | 0.24 | 0.78 |
| fatty amide C20:1 | 0.15 | 0.10 | 0.22 | 0.15 | 0.10 | 0.23 | 0.15 | 0.09 | 0.21 | 0.15 | 0.09 | 0.21 | 0.97 | 1.00 |
| fatty amide C22:0 | 0.02 | 0.01 | 0.02 | 0.01 | 0.01 | 0.02 | 0.02 | 0.01 | 0.02 | 0.01 | 0.01 | 0.02 | 0.90 | 1.00 |
| FFA 10:0 | 0.13 | 0.11 | 0.15 | 0.13 | 0.11 | 0.15 | 0.13 | 0.11 | 0.15 | 0.12 | 0.11 | 0.15 | 0.89 | 1.00 |
| FFA 11:0 | 0.01 | 0.01 | 0.01 | 0.01 | 0.01 | 0.01 | 0.01 | 0.01 | 0.01 | 0.01 | 0.01 | 0.01 | 0.40 | 0.89 |
| FFA 12:0 | 0.19 | 0.15 | 0.27 | 0.21 | 0.16 | 0.26 | 0.19 | 0.16 | 0.26 | 0.20 | 0.17 | 0.25 | 0.84 | 1.00 |
| FFA 14:0 | 0.38 | 0.31 | 0.50 | 0.39 | 0.31 | 0.52 | 0.38 | 0.30 | 0.47 | 0.40 | 0.32 | 0.53 | 0.44 | 0.89 |
| FFA 14:1 | 0.08 | 0.06 | 0.13 | 0.09 | 0.06 | 0.16 | 0.07 | 0.06 | 0.12 | 0.09 | 0.06 | 0.13 | 0.25 | 0.78 |
| FFA 15:0 | 0.47 | 0.43 | 0.53 | 0.49 | 0.43 | 0.54 | 0.46 | 0.43 | 0.49 | 0.47 | 0.44 | 0.53 | 0.20 | 0.78 |
| FFA 16:0 | 116.62 | 110.25 | 126.95 | 116.90 | 110.41 | 134.32 | 115.76 | 108.14 | 125.87 | 119.30 | 111.31 | 129.45 | 0.37 | 0.88 |
| FFA 16:1 | 2.23 | 1.61 | 3.37 | 2.28 | 1.61 | 3.32 | 2.06 | 1.40 | 3.54 | 2.30 | 1.59 | 3.51 | 0.49 | 0.89 |
| FFA 16:2 | 0.09 | 0.06 | 0.13 | 0.10 | 0.08 | 0.14 | 0.09 | 0.06 | 0.13 | 0.09 | 0.07 | 0.16 | 0.16 | 0.74 |
| FFA 16:3 | 0.03 | 0.02 | 0.04 | 0.03 | 0.02 | 0.05 | 0.03 | 0.02 | 0.04 | 0.03 | 0.02 | 0.05 | 0.15 | 0.74 |
| FFA 17:0 | 1.08 | 1.00 | 1.18 | 1.09 | 1.03 | 1.25 | 1.08 | 0.99 | 1.16 | 1.14 | 1.02 | 1.24 | 0.08 | 0.74 |
| FFA 17:1 | 0.33 | 0.28 | 0.42 | 0.33 | 0.29 | 0.41 | 0.31 | 0.27 | 0.39 | 0.35 | 0.29 | 0.41 | 0.32 | 0.83 |
| FFA 18:0 | 39.11 | 35.37 | 43.83 | 39.62 | 37.06 | 45.37 | 38.83 | 35.37 | 42.49 | 40.75 | 36.99 | 44.44 | 0.10 | 0.74 |
| FFA 18:1 | 121.45 | 109.36 | 139.87 | 124.81 | 112.86 | 147.33 | 118.88 | 109.43 | 135.49 | 124.07 | 113.19 | 142.24 | 0.16 | 0.74 |
| FFA 18:2 | 44.27 | 36.35 | 62.21 | 46.91 | 39.05 | 67.97 | 43.38 | 37.78 | 52.85 | 46.10 | 39.04 | 64.87 | 0.20 | 0.78 |
| FFA 18:3 | 0.28 | 0.18 | 0.42 | 0.29 | 0.18 | 0.51 | 0.25 | 0.17 | 0.38 | 0.29 | 0.17 | 0.51 | 0.39 | 0.88 |
| FFA 19:0 | 0.14 | 0.12 | 0.16 | 0.14 | 0.13 | 0.16 | 0.13 | 0.13 | 0.15 | 0.14 | 0.13 | 0.16 | 0.22 | 0.78 |
| FFA 19:1 | 0.22 | 0.20 | 0.26 | 0.23 | 0.20 | 0.27 | 0.22 | 0.20 | 0.25 | 0.23 | 0.21 | 0.28 | 0.29 | 0.79 |
| FFA 20:0 | 2.27 | 2.09 | 2.47 | 2.29 | 2.10 | 2.45 | 2.18 | 2.05 | 2.42 | 2.26 | 2.12 | 2.44 | 0.46 | 0.89 |
| FFA 20:1 | 1.43 | 1.27 | 1.70 | 1.49 | 1.36 | 1.71 | 1.40 | 1.28 | 1.67 | 1.53 | 1.36 | 1.80 | 0.05 | 0.74 |
| FFA 20:2 | 0.39 | 0.27 | 0.60 | 0.45 | 0.28 | 0.75 | 0.34 | 0.24 | 0.51 | 0.41 | 0.28 | 0.64 | 0.11 | 0.74 |
| FFA 20:3 | 0.41 | 0.28 | 0.54 | 0.40 | 0.29 | 0.60 | 0.38 | 0.26 | 0.51 | 0.42 | 0.29 | 0.55 | 0.34 | 0.86 |
| FFA 20:4 | 2.11 | 1.76 | 2.65 | 2.37 | 1.80 | 2.97 | 2.08 | 1.58 | 2.83 | 2.28 | 1.86 | 3.08 | 0.16 | 0.74 |
| FFA 20:5 | 0.22 | 0.17 | 0.38 | 0.26 | 0.15 | 0.40 | 0.24 | 0.13 | 0.33 | 0.24 | 0.16 | 0.40 | 0.55 | 0.89 |
| FFA 22:0 | 0.04 | 0.03 | 0.06 | 0.04 | 0.04 | 0.07 | 0.04 | 0.03 | 0.05 | 0.05 | 0.04 | 0.06 | 0.24 | 0.78 |
| FFA 22:1 | 0.14 | 0.12 | 0.18 | 0.14 | 0.11 | 0.20 | 0.14 | 0.11 | 0.17 | 0.16 | 0.12 | 0.21 | 0.08 | 0.74 |
| FFA 22:2 | 0.01 | 0.01 | 0.02 | 0.02 | 0.01 | 0.02 | 0.01 | 0.01 | 0.02 | 0.01 | 0.01 | 0.02 | 0.09 | 0.74 |
| FFA 22:5 | 0.23 | 0.17 | 0.34 | 0.26 | 0.18 | 0.35 | 0.21 | 0.15 | 0.33 | 0.25 | 0.16 | 0.39 | 0.32 | 0.83 |
| FFA 22:6 | 1.59 | 1.11 | 2.19 | 1.73 | 1.28 | 2.49 | 1.41 | 0.95 | 2.18 | 1.65 | 1.19 | 2.49 | 0.04 | 0.74 |
| FFA 24:0 | 0.72 | 0.65 | 0.79 | 0.75 | 0.65 | 0.81 | 0.71 | 0.65 | 0.78 | 0.73 | 0.65 | 0.79 | 0.38 | 0.88 |
| FFA 24:1 | 0.09 | 0.07 | 0.13 | 0.09 | 0.07 | 0.13 | 0.08 | 0.06 | 0.10 | 0.09 | 0.07 | 0.13 | 0.12 | 0.74 |
| FFA 9:0 | 0.27 | 0.22 | 0.32 | 0.28 | 0.23 | 0.33 | 0.29 | 0.23 | 0.36 | 0.28 | 0.22 | 0.34 | 0.10 | 0.74 |
| FFA C22:1 | 1.84 | 1.20 | 2.65 | 1.72 | 1.12 | 2.92 | 1.73 | 1.25 | 2.57 | 1.59 | 1.11 | 2.69 | 0.95 | 1.00 |
| ffa:oeratio | 0.01 | 0.01 | 0.01 | 0.01 | 0.01 | 0.01 | 0.01 | 0.01 | 0.01 | 0.01 | 0.01 | 0.01 | 0.10 | 0.74 |
| FFAe | 337.62 | 306.12 | 385.37 | 344.93 | 313.45 | 419.46 | 328.28 | 304.54 | 372.11 | 347.19 | 313.43 | 402.69 | 0.16 | 0.74 |
| FFAo | 2.54 | 2.33 | 2.85 | 2.64 | 2.37 | 3.01 | 2.53 | 2.37 | 2.72 | 2.65 | 2.40 | 2.91 | 0.21 | 0.78 |
| FFAs | 340.13 | 308.56 | 388.04 | 347.44 | 315.93 | 422.45 | 330.88 | 306.92 | 374.79 | 349.90 | 315.79 | 405.57 | 0.16 | 0.74 |
| Hexadecadienoylcarnitine C16:2 | 0.02 | 0.01 | 0.02 | 0.01 | 0.01 | 0.02 | 0.01 | 0.01 | 0.02 | 0.01 | 0.01 | 0.02 | 0.26 | 0.78 |
| Hexadecenoylcarnitine C16:1 | 0.02 | 0.02 | 0.03 | 0.02 | 0.01 | 0.03 | 0.02 | 0.02 | 0.03 | 0.02 | 0.02 | 0.03 | 0.20 | 0.78 |
| Hexanoylcarnitine(C6) | 0.02 | 0.02 | 0.03 | 0.02 | 0.02 | 0.03 | 0.02 | 0.02 | 0.03 | 0.02 | 0.02 | 0.03 | 0.24 | 0.78 |
| L-Acetylcarnitine(C2) | 0.19 | 0.16 | 0.22 | 0.17 | 0.13 | 0.22 | 0.16 | 0.13 | 0.20 | 0.17 | 0.14 | 0.21 | 0.01 | 0.30 |
| L-Carnitine | 0.27 | 0.24 | 0.30 | 0.25 | 0.22 | 0.30 | 0.26 | 0.23 | 0.29 | 0.27 | 0.24 | 0.30 | 0.06 | 0.74 |
| linolenyl carnitine C18:3 | 0.01 | 0.01 | 0.01 | 0.01 | 0.01 | 0.01 | 0.01 | 0.01 | 0.01 | 0.01 | 0.01 | 0.01 | 0.83 | 1.00 |
| Linoleyl carnitine C18:2 | 0.11 | 0.08 | 0.13 | 0.11 | 0.09 | 0.13 | 0.09 | 0.08 | 0.13 | 0.10 | 0.09 | 0.13 | 0.52 | 0.89 |
| LPC 14:0 sn-1 | 0.37 | 0.29 | 0.47 | 0.39 | 0.27 | 0.49 | 0.37 | 0.30 | 0.50 | 0.39 | 0.28 | 0.49 | 0.99 | 1.00 |
| LPC 14:0 sn-2 | 0.03 | 0.02 | 0.03 | 0.03 | 0.02 | 0.04 | 0.03 | 0.02 | 0.03 | 0.03 | 0.02 | 0.04 | 0.92 | 1.00 |
| LPC 15:0 sn-1 | 0.11 | 0.09 | 0.13 | 0.11 | 0.09 | 0.14 | 0.11 | 0.09 | 0.14 | 0.12 | 0.10 | 0.14 | 0.35 | 0.88 |
| LPC 15:0 sn-2 | 0.01 | 0.01 | 0.01 | 0.01 | 0.01 | 0.01 | 0.01 | 0.01 | 0.01 | 0.01 | 0.01 | 0.01 | 0.96 | 1.00 |
| LPC 16:0 sn-1 | 16.45 | 14.92 | 18.08 | 16.52 | 14.80 | 19.85 | 16.23 | 14.63 | 18.37 | 17.30 | 15.52 | 19.64 | 0.28 | 0.79 |
| LPC 16:0 sn-2 | 1.83 | 1.64 | 2.05 | 1.86 | 1.59 | 2.27 | 1.81 | 1.53 | 2.02 | 1.93 | 1.70 | 2.24 | 0.14 | 0.74 |
| LPC 16:1 sn-1 | 0.68 | 0.53 | 0.81 | 0.65 | 0.54 | 0.86 | 0.70 | 0.55 | 0.80 | 0.69 | 0.55 | 0.88 | 0.68 | 0.95 |
| LPC 16:1 sn-2 | 0.04 | 0.03 | 0.05 | 0.04 | 0.03 | 0.05 | 0.04 | 0.03 | 0.05 | 0.05 | 0.04 | 0.06 | 0.39 | 0.88 |
| LPC 17:0 | 0.21 | 0.17 | 0.25 | 0.22 | 0.18 | 0.29 | 0.22 | 0.18 | 0.26 | 0.23 | 0.19 | 0.27 | 0.26 | 0.78 |
| LPC 18:0 sn-1 | 6.23 | 5.44 | 7.17 | 6.59 | 5.63 | 7.50 | 6.21 | 5.34 | 7.47 | 6.76 | 5.72 | 7.84 | 0.06 | 0.74 |
| LPC 18:0 sn-2 | 0.36 | 0.31 | 0.42 | 0.38 | 0.32 | 0.44 | 0.36 | 0.30 | 0.45 | 0.39 | 0.32 | 0.46 | 0.22 | 0.78 |
| LPC 18:1 sn-1 | 3.34 | 2.91 | 3.73 | 3.41 | 2.97 | 4.16 | 3.36 | 2.87 | 4.17 | 3.64 | 3.18 | 4.38 | 0.05 | 0.74 |
| LPC 18:1 sn-2 | 0.20 | 0.17 | 0.24 | 0.20 | 0.17 | 0.26 | 0.21 | 0.17 | 0.26 | 0.23 | 0.19 | 0.28 | 0.10 | 0.74 |
| LPC 18:2 sn-1 | 9.17 | 7.67 | 10.23 | 8.92 | 7.60 | 10.70 | 8.80 | 7.38 | 10.12 | 9.40 | 7.26 | 11.25 | 0.58 | 0.90 |
| LPC 18:2 sn-2 | 0.82 | 0.67 | 0.94 | 0.77 | 0.66 | 1.02 | 0.76 | 0.62 | 0.93 | 0.83 | 0.64 | 1.04 | 0.52 | 0.89 |
| LPC 18:3 sn-1 | 0.91 | 0.83 | 1.05 | 0.88 | 0.76 | 1.08 | 0.91 | 0.76 | 1.08 | 0.96 | 0.81 | 1.15 | 0.44 | 0.89 |
| LPC 18:3 sn-2 | 0.13 | 0.10 | 0.16 | 0.12 | 0.10 | 0.16 | 0.12 | 0.09 | 0.16 | 0.13 | 0.10 | 0.16 | 0.63 | 0.94 |
| LPC 20:0 sn-1 | 0.02 | 0.02 | 0.03 | 0.03 | 0.02 | 0.03 | 0.02 | 0.02 | 0.03 | 0.03 | 0.02 | 0.03 | 0.01 | 0.30 |
| LPC 20:0 sn-2 | 0.00 | 0.00 | 0.00 | 0.00 | 0.00 | 0.00 | 0.00 | 0.00 | 0.00 | 0.00 | 0.00 | 0.00 | 0.10 | 0.74 |
| LPC 20:1 sn-1 | 0.04 | 0.03 | 0.05 | 0.04 | 0.03 | 0.05 | 0.04 | 0.03 | 0.05 | 0.04 | 0.03 | 0.05 | 0.00 | 0.13 |
| LPC 20:1 sn-2 | 0.00 | 0.00 | 0.00 | 0.00 | 0.00 | 0.00 | 0.00 | 0.00 | 0.00 | 0.00 | 0.00 | 0.00 | 0.00 | 0.26 |
| LPC 20:2 sn-1 | 0.05 | 0.04 | 0.06 | 0.05 | 0.05 | 0.07 | 0.05 | 0.04 | 0.06 | 0.06 | 0.04 | 0.07 | 0.00 | 0.13 |
| LPC 20:2 sn-2 | 0.00 | 0.00 | 0.00 | 0.00 | 0.00 | 0.00 | 0.00 | 0.00 | 0.00 | 0.00 | 0.00 | 0.00 | 0.01 | 0.31 |
| LPC 20:3 sn-1 | 0.40 | 0.30 | 0.51 | 0.39 | 0.29 | 0.54 | 0.39 | 0.30 | 0.48 | 0.40 | 0.33 | 0.56 | 0.51 | 0.89 |
| LPC 20:4 sn-1 | 2.52 | 2.01 | 3.14 | 2.50 | 1.91 | 3.14 | 2.42 | 1.94 | 3.16 | 2.47 | 1.96 | 3.15 | 0.96 | 1.00 |
| LPC 20:5 | 0.19 | 0.12 | 0.32 | 0.20 | 0.14 | 0.32 | 0.20 | 0.10 | 0.39 | 0.20 | 0.13 | 0.33 | 0.88 | 1.00 |
| LPC 22:0 | 0.01 | 0.00 | 0.01 | 0.01 | 0.01 | 0.01 | 0.01 | 0.00 | 0.01 | 0.01 | 0.01 | 0.01 | 0.07 | 0.74 |
| LPC 22:1 | 0.00 | 0.00 | 0.00 | 0.00 | 0.00 | 0.00 | 0.00 | 0.00 | 0.00 | 0.00 | 0.00 | 0.00 | 0.11 | 0.74 |
| LPC 22:4 | 0.02 | 0.01 | 0.02 | 0.02 | 0.01 | 0.02 | 0.02 | 0.01 | 0.02 | 0.02 | 0.01 | 0.02 | 0.89 | 1.00 |
| LPC 22:5 sn-1 | 0.07 | 0.05 | 0.09 | 0.07 | 0.06 | 0.09 | 0.07 | 0.06 | 0.09 | 0.07 | 0.06 | 0.09 | 0.53 | 0.89 |
| LPC 22:6 sn-1 | 0.63 | 0.45 | 0.82 | 0.68 | 0.50 | 0.90 | 0.66 | 0.50 | 0.83 | 0.70 | 0.53 | 0.92 | 0.12 | 0.74 |
| LPC 24:0 | 0.01 | 0.01 | 0.01 | 0.01 | 0.01 | 0.01 | 0.01 | 0.01 | 0.01 | 0.01 | 0.01 | 0.01 | 0.48 | 0.89 |
| LPC O-16:0 | 0.13 | 0.11 | 0.15 | 0.13 | 0.11 | 0.17 | 0.14 | 0.11 | 0.16 | 0.14 | 0.11 | 0.17 | 0.26 | 0.78 |
| LPC O-16:1 | 0.17 | 0.14 | 0.20 | 0.18 | 0.15 | 0.21 | 0.18 | 0.15 | 0.21 | 0.18 | 0.15 | 0.22 | 0.42 | 0.89 |
| LPC O-18:0 | 0.02 | 0.02 | 0.03 | 0.02 | 0.02 | 0.03 | 0.02 | 0.02 | 0.03 | 0.03 | 0.02 | 0.03 | 0.16 | 0.74 |
| LPC O-18:1 | 0.07 | 0.06 | 0.08 | 0.07 | 0.06 | 0.09 | 0.07 | 0.06 | 0.09 | 0.08 | 0.07 | 0.10 | 0.03 | 0.59 |
| LPC P-18:0 | 0.01 | 0.01 | 0.02 | 0.02 | 0.01 | 0.02 | 0.01 | 0.01 | 0.02 | 0.01 | 0.01 | 0.02 | 0.29 | 0.79 |
| LPC P-18:1 | 0.01 | 0.01 | 0.01 | 0.01 | 0.01 | 0.01 | 0.01 | 0.01 | 0.01 | 0.01 | 0.01 | 0.01 | 0.07 | 0.74 |
| LPCS | 45.66 | 41.31 | 49.51 | 45.90 | 40.06 | 54.64 | 43.89 | 40.08 | 50.87 | 47.48 | 42.12 | 54.09 | 0.17 | 0.76 |
| lpcs:pc:ratio | 0.57 | 0.49 | 0.63 | 0.59 | 0.52 | 0.68 | 0.57 | 0.49 | 0.64 | 0.58 | 0.53 | 0.66 | 0.29 | 0.79 |
| LPE 16:0 | 0.17 | 0.14 | 0.21 | 0.18 | 0.14 | 0.22 | 0.17 | 0.15 | 0.19 | 0.18 | 0.14 | 0.21 | 0.83 | 1.00 |
| LPE 16:1 | 0.01 | 0.00 | 0.01 | 0.01 | 0.00 | 0.01 | 0.01 | 0.00 | 0.01 | 0.01 | 0.00 | 0.01 | 0.51 | 0.89 |
| LPE 18:0 sn-1 | 0.19 | 0.16 | 0.23 | 0.19 | 0.16 | 0.25 | 0.19 | 0.16 | 0.22 | 0.20 | 0.17 | 0.24 | 0.41 | 0.89 |
| LPE 18:0 sn-2 | 0.01 | 0.01 | 0.01 | 0.01 | 0.01 | 0.01 | 0.01 | 0.01 | 0.01 | 0.01 | 0.01 | 0.02 | 0.45 | 0.89 |
| LPE 18:1 sn-1 | 0.12 | 0.10 | 0.15 | 0.12 | 0.10 | 0.15 | 0.12 | 0.10 | 0.14 | 0.12 | 0.10 | 0.15 | 0.87 | 1.00 |
| LPE 18:1 sn-2 | 0.01 | 0.01 | 0.01 | 0.01 | 0.01 | 0.01 | 0.01 | 0.01 | 0.01 | 0.01 | 0.01 | 0.01 | 0.58 | 0.90 |
| LPE 18:2 sn-1 | 0.47 | 0.41 | 0.59 | 0.50 | 0.36 | 0.61 | 0.42 | 0.36 | 0.60 | 0.46 | 0.35 | 0.64 | 0.67 | 0.95 |
| LPE 18:2 sn-2 | 0.04 | 0.03 | 0.05 | 0.04 | 0.03 | 0.05 | 0.03 | 0.03 | 0.05 | 0.04 | 0.03 | 0.05 | 0.45 | 0.89 |
| LPE 18:3sn-1 | 0.00 | 0.00 | 0.01 | 0.00 | 0.00 | 0.01 | 0.00 | 0.00 | 0.01 | 0.00 | 0.00 | 0.01 | 0.71 | 0.97 |
| LPE 20:3 | 0.02 | 0.01 | 0.02 | 0.02 | 0.01 | 0.02 | 0.02 | 0.01 | 0.02 | 0.02 | 0.01 | 0.02 | 0.87 | 1.00 |
| LPE 20:4 sn-1 | 0.33 | 0.27 | 0.42 | 0.32 | 0.25 | 0.40 | 0.32 | 0.27 | 0.39 | 0.33 | 0.25 | 0.43 | 0.64 | 0.94 |
| LPE 20:4 sn-2 | 0.03 | 0.03 | 0.04 | 0.03 | 0.03 | 0.04 | 0.03 | 0.03 | 0.04 | 0.03 | 0.03 | 0.04 | 0.57 | 0.90 |
| LPE 20:5 sn-1 | 0.02 | 0.01 | 0.03 | 0.02 | 0.01 | 0.03 | 0.02 | 0.01 | 0.03 | 0.02 | 0.01 | 0.03 | 1.00 | 1.00 |
| LPE 22:4 sn-1 | 0.02 | 0.01 | 0.02 | 0.01 | 0.01 | 0.02 | 0.01 | 0.01 | 0.02 | 0.01 | 0.01 | 0.02 | 0.45 | 0.89 |
| LPE 22:6 sn-1 | 0.28 | 0.22 | 0.34 | 0.27 | 0.21 | 0.36 | 0.26 | 0.21 | 0.33 | 0.27 | 0.23 | 0.35 | 0.74 | 0.97 |
| Octanoylcarnitine(C8) | 0.08 | 0.05 | 0.11 | 0.06 | 0.05 | 0.10 | 0.07 | 0.05 | 0.09 | 0.07 | 0.04 | 0.10 | 0.31 | 0.83 |
| Oleoylcarnitine C18:1 | 0.08 | 0.07 | 0.10 | 0.08 | 0.07 | 0.10 | 0.08 | 0.06 | 0.11 | 0.09 | 0.07 | 0.11 | 0.95 | 1.00 |
| Palmitoylcarnitine C16 | 0.08 | 0.07 | 0.09 | 0.07 | 0.06 | 0.09 | 0.07 | 0.06 | 0.09 | 0.08 | 0.06 | 0.09 | 0.50 | 0.89 |
| PC 16:0:18:2 | 18.87 | 16.59 | 22.94 | 17.60 | 15.32 | 21.42 | 18.24 | 15.62 | 20.79 | 19.17 | 15.90 | 23.66 | 0.51 | 0.89 |
| PC 30:0 | 0.15 | 0.12 | 0.21 | 0.15 | 0.11 | 0.22 | 0.15 | 0.11 | 0.22 | 0.15 | 0.11 | 0.21 | 0.97 | 1.00 |
| PC 32:1 | 0.97 | 0.68 | 1.31 | 0.85 | 0.58 | 1.22 | 0.87 | 0.63 | 1.24 | 0.90 | 0.61 | 1.22 | 0.75 | 0.97 |
| PC 32:2 | 1.07 | 0.80 | 1.24 | 1.01 | 0.81 | 1.27 | 1.01 | 0.76 | 1.29 | 0.99 | 0.77 | 1.25 | 0.98 | 1.00 |
| PC 33:1 | 0.11 | 0.10 | 0.14 | 0.12 | 0.09 | 0.14 | 0.12 | 0.08 | 0.16 | 0.12 | 0.09 | 0.15 | 0.99 | 1.00 |
| PC 34:0 | 1.37 | 1.20 | 1.58 | 1.40 | 1.14 | 1.76 | 1.41 | 1.18 | 1.72 | 1.41 | 1.19 | 1.67 | 0.95 | 1.00 |
| PC 34:1 | 9.98 | 8.79 | 12.05 | 9.48 | 8.26 | 12.18 | 10.07 | 8.42 | 12.13 | 10.14 | 8.52 | 12.22 | 0.61 | 0.92 |
| PC 34:3 | 2.11 | 1.81 | 2.51 | 2.21 | 1.72 | 2.68 | 2.15 | 1.71 | 2.61 | 2.08 | 1.82 | 2.49 | 0.93 | 1.00 |
| PC 34:4 | 0.24 | 0.18 | 0.32 | 0.26 | 0.19 | 0.32 | 0.24 | 0.18 | 0.32 | 0.24 | 0.16 | 0.33 | 0.96 | 1.00 |
| PC 35:2 | 0.84 | 0.70 | 1.01 | 0.84 | 0.71 | 1.06 | 0.83 | 0.71 | 1.04 | 0.90 | 0.72 | 1.03 | 0.90 | 1.00 |
| PC 35:3 | 0.11 | 0.09 | 0.14 | 0.11 | 0.09 | 0.13 | 0.11 | 0.09 | 0.14 | 0.12 | 0.10 | 0.14 | 0.93 | 1.00 |
| PC 36:2 | 11.81 | 9.84 | 13.72 | 11.05 | 8.98 | 13.93 | 11.24 | 9.81 | 13.59 | 12.04 | 9.61 | 15.11 | 0.59 | 0.91 |
| PC 36:3 | 7.90 | 6.94 | 9.24 | 7.65 | 6.56 | 9.34 | 8.47 | 6.63 | 9.47 | 8.39 | 6.78 | 10.06 | 0.68 | 0.95 |
| PC 36:4 | 6.58 | 5.11 | 7.93 | 5.91 | 4.80 | 7.89 | 6.43 | 4.97 | 8.05 | 6.64 | 4.68 | 8.39 | 0.60 | 0.92 |
| PC 36:5 | 2.11 | 1.60 | 3.02 | 2.45 | 1.51 | 3.04 | 2.17 | 1.41 | 3.52 | 2.26 | 1.66 | 3.06 | 0.97 | 1.00 |
| PC 36:6 | 0.07 | 0.05 | 0.09 | 0.08 | 0.05 | 0.10 | 0.08 | 0.05 | 0.11 | 0.08 | 0.06 | 0.11 | 0.38 | 0.88 |
| PC 38:4 | 4.45 | 3.66 | 5.36 | 4.18 | 3.29 | 5.21 | 4.65 | 3.35 | 5.59 | 4.25 | 3.24 | 5.58 | 0.51 | 0.89 |
| PC 38:5 | 1.94 | 1.41 | 2.82 | 1.73 | 1.48 | 2.69 | 2.15 | 1.44 | 3.01 | 1.90 | 1.46 | 3.13 | 0.55 | 0.89 |
| PC 38:6 | 7.71 | 6.60 | 8.65 | 7.80 | 6.80 | 9.23 | 7.64 | 6.47 | 9.77 | 8.34 | 7.18 | 9.58 | 0.16 | 0.74 |
| PC 38:7 | 0.60 | 0.48 | 0.74 | 0.59 | 0.46 | 0.78 | 0.61 | 0.49 | 0.74 | 0.64 | 0.47 | 0.79 | 0.73 | 0.97 |
| PC O-34:2 | 0.18 | 0.15 | 0.24 | 0.18 | 0.14 | 0.22 | 0.18 | 0.15 | 0.23 | 0.19 | 0.16 | 0.25 | 0.71 | 0.97 |
| PC O-36:4 | 1.43 | 1.10 | 1.68 | 1.34 | 1.09 | 1.58 | 1.33 | 1.05 | 1.56 | 1.38 | 1.14 | 1.64 | 0.47 | 0.89 |
| PC O-36:5 | 0.03 | 0.02 | 0.04 | 0.02 | 0.02 | 0.04 | 0.03 | 0.02 | 0.03 | 0.03 | 0.02 | 0.04 | 0.71 | 0.97 |
| PC O-38:5 | 1.27 | 1.08 | 1.52 | 1.24 | 1.03 | 1.54 | 1.29 | 1.07 | 1.49 | 1.33 | 1.08 | 1.61 | 0.75 | 0.97 |
| PC O-38:6 | 0.30 | 0.25 | 0.37 | 0.30 | 0.23 | 0.36 | 0.29 | 0.25 | 0.37 | 0.30 | 0.24 | 0.37 | 0.92 | 1.00 |
| PCS | 79.62 | 71.26 | 89.94 | 75.14 | 67.44 | 94.86 | 80.45 | 68.47 | 91.37 | 81.12 | 68.22 | 96.93 | 0.56 | 0.90 |
| PE 34:2 | 0.07 | 0.05 | 0.09 | 0.07 | 0.04 | 0.09 | 0.06 | 0.05 | 0.09 | 0.06 | 0.05 | 0.09 | 0.89 | 1.00 |
| PE 38:6 | 0.18 | 0.13 | 0.22 | 0.17 | 0.12 | 0.25 | 0.16 | 0.13 | 0.22 | 0.17 | 0.13 | 0.23 | 0.99 | 1.00 |
| PE O-34:3 | 0.07 | 0.05 | 0.09 | 0.07 | 0.05 | 0.09 | 0.06 | 0.05 | 0.09 | 0.07 | 0.05 | 0.09 | 0.85 | 1.00 |
| PE O-36:5 | 0.28 | 0.22 | 0.35 | 0.27 | 0.21 | 0.35 | 0.26 | 0.21 | 0.31 | 0.26 | 0.21 | 0.36 | 0.78 | 0.99 |
| PE O-38:6 | 0.22 | 0.15 | 0.27 | 0.20 | 0.15 | 0.25 | 0.20 | 0.16 | 0.24 | 0.19 | 0.15 | 0.27 | 0.90 | 1.00 |
| PE O-38:7 | 0.14 | 0.10 | 0.18 | 0.13 | 0.10 | 0.19 | 0.13 | 0.10 | 0.17 | 0.13 | 0.11 | 0.20 | 0.67 | 0.95 |
| Propionyl-L-carnitine(C3) | 0.02 | 0.01 | 0.03 | 0.02 | 0.01 | 0.03 | 0.02 | 0.01 | 0.03 | 0.02 | 0.01 | 0.02 | 0.28 | 0.79 |
| SM 32:1 | 0.77 | 0.63 | 0.98 | 0.77 | 0.61 | 0.92 | 0.73 | 0.55 | 0.95 | 0.80 | 0.59 | 0.97 | 0.90 | 1.00 |
| SM 32:2 | 0.05 | 0.04 | 0.06 | 0.05 | 0.04 | 0.06 | 0.05 | 0.04 | 0.06 | 0.05 | 0.04 | 0.06 | 0.93 | 1.00 |
| SM 33:1 | 0.40 | 0.36 | 0.50 | 0.43 | 0.33 | 0.52 | 0.39 | 0.32 | 0.48 | 0.44 | 0.33 | 0.52 | 0.47 | 0.89 |
| SM 33:2 | 0.01 | 0.01 | 0.01 | 0.01 | 0.01 | 0.01 | 0.01 | 0.01 | 0.01 | 0.01 | 0.01 | 0.01 | 0.74 | 0.97 |
| SM 34:1 | 10.47 | 9.36 | 11.48 | 10.29 | 8.47 | 11.99 | 10.10 | 8.66 | 11.82 | 10.40 | 9.35 | 12.27 | 0.67 | 0.95 |
| SM 34:2 | 2.79 | 2.39 | 3.07 | 2.67 | 2.41 | 3.14 | 2.60 | 2.27 | 3.10 | 2.83 | 2.47 | 3.13 | 0.41 | 0.89 |
| SM 35:2 | 0.03 | 0.03 | 0.04 | 0.03 | 0.03 | 0.04 | 0.03 | 0.03 | 0.04 | 0.03 | 0.03 | 0.04 | 0.83 | 1.00 |
| SM 36:1 | 1.69 | 1.44 | 1.96 | 1.58 | 1.31 | 2.05 | 1.55 | 1.30 | 1.91 | 1.59 | 1.30 | 2.06 | 0.22 | 0.78 |
| SM 36:2 | 1.20 | 1.05 | 1.48 | 1.22 | 1.02 | 1.43 | 1.18 | 0.97 | 1.39 | 1.19 | 0.98 | 1.53 | 0.55 | 0.89 |
| SM 36:3 | 0.07 | 0.06 | 0.08 | 0.07 | 0.06 | 0.08 | 0.07 | 0.06 | 0.08 | 0.07 | 0.06 | 0.08 | 0.77 | 0.98 |
| sm:pc | 0.18 | 0.17 | 0.19 | 0.18 | 0.16 | 0.19 | 0.17 | 0.16 | 0.20 | 0.18 | 0.16 | 0.20 | 0.74 | 0.97 |
| SMS | 17.47 | 15.97 | 19.82 | 17.70 | 14.81 | 20.60 | 16.93 | 14.76 | 19.74 | 17.93 | 15.29 | 20.42 | 0.64 | 0.94 |
| Sphingosine | 0.00 | 0.00 | 0.00 | 0.00 | 0.00 | 0.00 | 0.00 | 0.00 | 0.00 | 0.00 | 0.00 | 0.00 | 0.69 | 0.96 |
| Sphingosine-1-phosphate | 0.08 | 0.06 | 0.10 | 0.08 | 0.06 | 0.11 | 0.07 | 0.05 | 0.10 | 0.08 | 0.06 | 0.10 | 0.15 | 0.74 |
| Stearoylcarnitine C18 | 0.01 | 0.01 | 0.02 | 0.01 | 0.01 | 0.02 | 0.01 | 0.01 | 0.02 | 0.01 | 0.01 | 0.02 | 0.23 | 0.78 |
| Tetradecadiencarnitine C14:2 | 0.02 | 0.02 | 0.03 | 0.02 | 0.02 | 0.03 | 0.02 | 0.01 | 0.03 | 0.02 | 0.01 | 0.04 | 0.45 | 0.89 |
| Tetradecanoylcarnitine C14 | 0.01 | 0.00 | 0.01 | 0.00 | 0.00 | 0.01 | 0.00 | 0.00 | 0.01 | 0.00 | 0.00 | 0.01 | 0.50 | 0.89 |
| Tetradecenoylcarnitine C14:1 | 0.03 | 0.02 | 0.04 | 0.02 | 0.02 | 0.04 | 0.03 | 0.02 | 0.03 | 0.03 | 0.02 | 0.04 | 0.38 | 0.88 |
| Valerylcarnitine(C5) | 0.07 | 0.06 | 0.08 | 0.06 | 0.05 | 0.08 | 0.06 | 0.05 | 0.08 | 0.07 | 0.05 | 0.08 | 0.12 | 0.74 |
