# Supplementary material for: Combined berberine and probiotic treatment as an effective regimen for improving postprandial hyperlipidemia in type 2 diabetes patients: a double blinded placebo controlled randomized study
Source: Gut Microbes. 2021 Dec 20;14(1):2003176. doi: 10.1080/19490976.2021.2003176 (PMC8726654; doi:10.1080/19490976.2021.2003176)
Supplement: Supplemental Material [file KGMI_A_2003176_SM5262.zip › Supplementary information/Data Set 2.docx]

Data Set 2. Changes of plasma postprandial lipid species from baseline to post treatment in all arms

|  | **Plac** | | | | | | Prob | | | | | | BBR | | | | | | Prob+BBR | | | | | |
| --- | --- | --- | --- | --- | --- | --- | --- | --- | --- | --- | --- | --- | --- | --- | --- | --- | --- | --- | --- | --- | --- | --- | --- | --- |
|  | median_v5 | v5_0.25 | v5_0.75 | p value | P.adjust | z score | median_v5 | v5_0.25 | v5_0.75 | p value | P.adjust | z score | median_v5 | v5_0.25 | v5_0.75 | p value | P.adjust | z score | median_v5 | v5_0.25 | v5_0.75 | p value | P.adjust | z score |
| 2-Octenoylcarnitine(C8:1) | 0.17 | 0.13 | 0.23 | 0.50 | 0.70 | -0.68 | 0.16 | 0.12 | 0.22 | 0.86 | 0.92 | -0.18 | 0.17 | 0.09 | 0.23 | 0.50 | 0.70 | -0.67 | 0.15 | 0.10 | 0.22 | 0.01 | 0.07 | -2.47 |
| Butyrylcarnitine(C4) | 0.08 | 0.06 | 0.09 | 0.06 | 0.18 | 1.89 | 0.08 | 0.06 | 0.09 | 0.00 | 0.00 | 3.73 | 0.07 | 0.05 | 0.10 | 0.10 | 0.26 | 1.65 | 0.08 | 0.06 | 0.09 | 0.01 | 0.04 | 2.69 |
| Carnitine C10:1 | 0.13 | 0.10 | 0.16 | 0.04 | 0.15 | -2.05 | 0.14 | 0.09 | 0.18 | 0.20 | 0.38 | -1.28 | 0.11 | 0.09 | 0.14 | 0.01 | 0.04 | -2.75 | 0.11 | 0.08 | 0.15 | 0.00 | 0.00 | -3.57 |
| Carnitine C12:0 | 0.02 | 0.02 | 0.03 | 0.85 | 0.91 | -0.19 | 0.02 | 0.01 | 0.03 | 0.83 | 0.91 | -0.21 | 0.02 | 0.01 | 0.02 | 0.07 | 0.20 | -1.81 | 0.02 | 0.01 | 0.03 | 0.00 | 0.01 | -3.47 |
| Carnitine C12:1 | 0.03 | 0.02 | 0.04 | 0.10 | 0.26 | -1.64 | 0.02 | 0.02 | 0.04 | 0.73 | 0.84 | -0.34 | 0.02 | 0.02 | 0.03 | 0.01 | 0.06 | -2.52 | 0.02 | 0.02 | 0.03 | 0.00 | 0.00 | -4.38 |
| carnitine C12:2 | 0.01 | 0.01 | 0.01 | 0.01 | 0.07 | -2.46 | 0.01 | 0.01 | 0.01 | 0.12 | 0.29 | -1.57 | 0.01 | 0.00 | 0.01 | 0.00 | 0.03 | -2.85 | 0.01 | 0.00 | 0.01 | 0.01 | 0.07 | -2.47 |
| carnitine C14:3 | 0.00 | 0.00 | 0.00 | 0.79 | 0.88 | -0.27 | 0.00 | 0.00 | 0.00 | 0.98 | 0.99 | -0.03 | 0.00 | 0.00 | 0.00 | 0.28 | 0.48 | -1.09 | 0.00 | 0.00 | 0.00 | 0.03 | 0.11 | -2.21 |
| Decadienoylcarnitine C10:3 | 0.12 | 0.08 | 0.17 | 0.99 | 0.99 | -0.01 | 0.11 | 0.08 | 0.15 | 0.51 | 0.70 | -0.67 | 0.11 | 0.06 | 0.16 | 0.20 | 0.38 | -1.27 | 0.09 | 0.06 | 0.15 | 0.02 | 0.08 | -2.39 |
| Decanoylcarnitine(C10) | 0.09 | 0.07 | 0.13 | 0.56 | 0.73 | -0.59 | 0.09 | 0.06 | 0.12 | 0.35 | 0.56 | -0.93 | 0.07 | 0.05 | 0.10 | 0.01 | 0.07 | -2.48 | 0.07 | 0.05 | 0.10 | 0.00 | 0.00 | -4.27 |
| DecenoylcarnitineC10:2 | 0.01 | 0.01 | 0.02 | 0.12 | 0.29 | -1.57 | 0.01 | 0.01 | 0.02 | 0.10 | 0.26 | -1.62 | 0.01 | 0.01 | 0.02 | 0.01 | 0.05 | -2.66 | 0.01 | 0.01 | 0.02 | 0.01 | 0.06 | -2.51 |
| fatty amide C20:1 | 0.17 | 0.12 | 0.26 | 0.04 | 0.15 | 2.05 | 0.17 | 0.12 | 0.26 | 0.08 | 0.23 | 1.74 | 0.16 | 0.12 | 0.27 | 0.03 | 0.13 | 2.11 | 0.16 | 0.12 | 0.25 | 0.14 | 0.31 | 1.49 |
| fatty amide C22:0 | 0.02 | 0.01 | 0.02 | 0.78 | 0.88 | -0.27 | 0.02 | 0.01 | 0.02 | 0.70 | 0.83 | 0.39 | 0.02 | 0.01 | 0.02 | 0.78 | 0.87 | 0.28 | 0.02 | 0.01 | 0.02 | 0.55 | 0.73 | -0.60 |
| FFA 10:0 | 0.11 | 0.08 | 0.14 | 0.00 | 0.02 | -3.07 | 0.11 | 0.09 | 0.14 | 0.01 | 0.04 | -2.74 | 0.12 | 0.09 | 0.14 | 0.00 | 0.00 | -4.07 | 0.11 | 0.08 | 0.14 | 0.00 | 0.00 | -4.28 |
| FFA 11:0 | 0.01 | 0.01 | 0.01 | 0.00 | 0.00 | -4.34 | 0.01 | 0.01 | 0.01 | 0.00 | 0.00 | -4.37 | 0.01 | 0.01 | 0.01 | 0.00 | 0.00 | -4.96 | 0.01 | 0.01 | 0.01 | 0.00 | 0.00 | -5.35 |
| FFA 12:0 | 0.16 | 0.12 | 0.20 | 0.00 | 0.01 | -3.48 | 0.17 | 0.12 | 0.24 | 0.10 | 0.26 | -1.62 | 0.16 | 0.13 | 0.20 | 0.00 | 0.03 | -2.87 | 0.16 | 0.13 | 0.21 | 0.00 | 0.00 | -4.01 |
| FFA 14:0 | 0.32 | 0.26 | 0.43 | 0.02 | 0.08 | -2.40 | 0.33 | 0.26 | 0.50 | 0.19 | 0.38 | -1.30 | 0.31 | 0.25 | 0.40 | 0.02 | 0.08 | -2.40 | 0.31 | 0.25 | 0.38 | 0.00 | 0.00 | -4.22 |
| FFA 14:1 | 0.08 | 0.05 | 0.12 | 0.85 | 0.91 | -0.19 | 0.08 | 0.05 | 0.13 | 0.36 | 0.56 | -0.92 | 0.06 | 0.05 | 0.11 | 0.38 | 0.59 | -0.87 | 0.07 | 0.05 | 0.13 | 0.16 | 0.34 | -1.42 |
| FFA 15:0 | 0.44 | 0.34 | 0.50 | 0.00 | 0.02 | -3.00 | 0.47 | 0.38 | 0.52 | 0.07 | 0.21 | -1.78 | 0.43 | 0.39 | 0.47 | 0.02 | 0.10 | -2.26 | 0.42 | 0.37 | 0.48 | 0.00 | 0.00 | -3.76 |
| FFA 16:0 | 115.69 | 65.55 | 127.41 | 0.06 | 0.18 | -1.90 | 117.60 | 96.40 | 128.68 | 0.05 | 0.17 | -1.95 | 113.22 | 100.01 | 123.81 | 0.03 | 0.13 | -2.12 | 110.94 | 73.13 | 123.56 | 0.00 | 0.00 | -3.58 |
| FFA 16:1 | 1.95 | 1.36 | 3.34 | 0.30 | 0.50 | -1.05 | 1.80 | 1.31 | 3.12 | 0.16 | 0.35 | -1.39 | 1.50 | 1.16 | 2.34 | 0.04 | 0.15 | -2.06 | 1.53 | 1.16 | 2.49 | 0.00 | 0.02 | -3.00 |
| FFA 16:2 | 0.09 | 0.06 | 0.14 | 0.94 | 0.97 | 0.07 | 0.10 | 0.06 | 0.14 | 0.31 | 0.52 | -1.01 | 0.08 | 0.06 | 0.11 | 0.55 | 0.73 | -0.60 | 0.08 | 0.05 | 0.12 | 0.13 | 0.31 | -1.50 |
| FFA 16:3 | 0.03 | 0.02 | 0.05 | 0.47 | 0.67 | 0.72 | 0.03 | 0.02 | 0.04 | 0.24 | 0.44 | -1.17 | 0.03 | 0.02 | 0.04 | 0.92 | 0.95 | -0.10 | 0.02 | 0.02 | 0.04 | 0.02 | 0.08 | -2.41 |
| FFA 17:0 | 1.06 | 0.79 | 1.19 | 0.04 | 0.15 | -2.04 | 1.07 | 0.94 | 1.20 | 0.04 | 0.16 | -2.01 | 1.02 | 0.95 | 1.15 | 0.04 | 0.15 | -2.06 | 1.02 | 0.93 | 1.13 | 0.00 | 0.00 | -4.02 |
| FFA 17:1 | 0.31 | 0.25 | 0.40 | 0.17 | 0.36 | -1.38 | 0.32 | 0.25 | 0.41 | 0.37 | 0.57 | -0.90 | 0.28 | 0.25 | 0.35 | 0.07 | 0.21 | -1.79 | 0.29 | 0.25 | 0.36 | 0.00 | 0.02 | -3.00 |
| FFA 18:0 | 37.86 | 33.17 | 43.90 | 0.12 | 0.29 | -1.57 | 38.26 | 33.08 | 42.64 | 0.02 | 0.10 | -2.30 | 37.71 | 32.92 | 41.10 | 0.06 | 0.18 | -1.92 | 36.47 | 32.71 | 40.77 | 0.00 | 0.00 | -4.01 |
| FFA 18:1 | 117.22 | 73.11 | 137.99 | 0.03 | 0.13 | -2.15 | 119.48 | 104.11 | 132.46 | 0.01 | 0.06 | -2.51 | 112.06 | 101.18 | 128.88 | 0.02 | 0.08 | -2.38 | 108.59 | 92.87 | 124.99 | 0.00 | 0.00 | -4.17 |
| FFA 18:2 | 42.94 | 34.27 | 56.33 | 0.31 | 0.51 | -1.02 | 45.12 | 33.94 | 58.69 | 0.18 | 0.37 | -1.34 | 39.63 | 33.74 | 50.92 | 0.40 | 0.60 | -0.85 | 39.23 | 32.44 | 51.96 | 0.04 | 0.15 | -2.02 |
| FFA 18:3 | 0.22 | 0.14 | 0.39 | 0.16 | 0.34 | -1.42 | 0.21 | 0.13 | 0.46 | 0.13 | 0.30 | -1.51 | 0.22 | 0.12 | 0.32 | 0.08 | 0.22 | -1.75 | 0.17 | 0.12 | 0.34 | 0.00 | 0.03 | -2.82 |
| FFA 19:0 | 0.13 | 0.11 | 0.14 | 0.00 | 0.00 | -3.62 | 0.13 | 0.11 | 0.15 | 0.00 | 0.00 | -4.32 | 0.13 | 0.11 | 0.14 | 0.00 | 0.00 | -3.68 | 0.13 | 0.11 | 0.14 | 0.00 | 0.00 | -5.71 |
| FFA 19:1 | 0.22 | 0.18 | 0.27 | 0.62 | 0.78 | -0.50 | 0.23 | 0.20 | 0.27 | 0.60 | 0.76 | -0.53 | 0.21 | 0.18 | 0.25 | 0.18 | 0.37 | -1.33 | 0.21 | 0.18 | 0.24 | 0.00 | 0.01 | -3.16 |
| FFA 20:0 | 2.07 | 1.72 | 2.21 | 0.00 | 0.00 | -4.97 | 2.11 | 1.77 | 2.21 | 0.00 | 0.00 | -5.08 | 2.08 | 1.93 | 2.20 | 0.00 | 0.01 | -3.27 | 2.06 | 1.75 | 2.20 | 0.00 | 0.00 | -5.05 |
| FFA 20:1 | 1.44 | 1.18 | 1.77 | 0.62 | 0.78 | -0.49 | 1.43 | 1.21 | 1.68 | 0.13 | 0.30 | -1.51 | 1.37 | 1.22 | 1.63 | 0.18 | 0.37 | -1.33 | 1.34 | 1.20 | 1.60 | 0.00 | 0.01 | -3.32 |
| FFA 20:2 | 0.43 | 0.30 | 0.67 | 0.12 | 0.29 | 1.55 | 0.43 | 0.30 | 0.70 | 0.52 | 0.71 | 0.65 | 0.32 | 0.24 | 0.60 | 0.43 | 0.63 | 0.79 | 0.40 | 0.25 | 0.56 | 0.66 | 0.80 | -0.44 |
| FFA 20:3 | 0.41 | 0.29 | 0.58 | 0.18 | 0.37 | 1.33 | 0.43 | 0.27 | 0.61 | 0.61 | 0.77 | 0.51 | 0.34 | 0.24 | 0.56 | 0.53 | 0.71 | 0.63 | 0.38 | 0.25 | 0.51 | 0.22 | 0.41 | -1.22 |
| FFA 20:4 | 2.00 | 1.57 | 2.86 | 0.98 | 0.99 | 0.03 | 2.07 | 1.61 | 2.79 | 0.22 | 0.41 | -1.22 | 1.92 | 1.52 | 2.73 | 0.77 | 0.87 | -0.29 | 1.85 | 1.45 | 2.45 | 0.01 | 0.04 | -2.71 |
| FFA 20:5 | 0.22 | 0.16 | 0.35 | 0.63 | 0.78 | -0.49 | 0.24 | 0.15 | 0.37 | 0.85 | 0.91 | 0.19 | 0.23 | 0.14 | 0.36 | 0.81 | 0.90 | 0.24 | 0.18 | 0.12 | 0.34 | 0.02 | 0.09 | -2.36 |
| FFA 22:0 | 0.03 | 0.02 | 0.05 | 0.01 | 0.04 | -2.77 | 0.04 | 0.02 | 0.05 | 0.00 | 0.01 | -3.14 | 0.03 | 0.02 | 0.04 | 0.04 | 0.13 | -2.11 | 0.03 | 0.02 | 0.04 | 0.00 | 0.00 | -3.75 |
| FFA 22:1 | 0.14 | 0.12 | 0.18 | 0.84 | 0.91 | 0.20 | 0.14 | 0.12 | 0.17 | 0.64 | 0.78 | -0.47 | 0.14 | 0.11 | 0.19 | 0.29 | 0.50 | 1.06 | 0.13 | 0.12 | 0.17 | 0.01 | 0.05 | -2.59 |
| FFA 22:2 | 0.01 | 0.01 | 0.02 | 0.24 | 0.44 | 1.17 | 0.01 | 0.01 | 0.02 | 0.79 | 0.88 | -0.26 | 0.01 | 0.01 | 0.02 | 0.76 | 0.87 | 0.30 | 0.01 | 0.01 | 0.02 | 0.27 | 0.48 | -1.11 |
| FFA 22:5 | 0.24 | 0.16 | 0.36 | 0.47 | 0.67 | 0.72 | 0.28 | 0.15 | 0.42 | 0.65 | 0.80 | 0.45 | 0.21 | 0.14 | 0.32 | 0.91 | 0.95 | 0.12 | 0.20 | 0.14 | 0.34 | 0.23 | 0.42 | -1.20 |
| FFA 22:6 | 1.42 | 1.08 | 2.24 | 0.88 | 0.93 | -0.15 | 1.66 | 1.09 | 2.52 | 0.85 | 0.91 | -0.19 | 1.47 | 0.96 | 2.13 | 0.57 | 0.74 | 0.57 | 1.37 | 1.00 | 2.18 | 0.16 | 0.35 | -1.41 |
| FFA 24:0 | 0.60 | 0.37 | 0.67 | 0.00 | 0.00 | -6.50 | 0.60 | 0.41 | 0.69 | 0.00 | 0.00 | -6.08 | 0.60 | 0.53 | 0.70 | 0.00 | 0.00 | -5.31 | 0.59 | 0.39 | 0.67 | 0.00 | 0.00 | -6.43 |
| FFA 24:1 | 0.10 | 0.07 | 0.14 | 0.18 | 0.37 | 1.33 | 0.10 | 0.07 | 0.13 | 0.56 | 0.73 | 0.58 | 0.09 | 0.07 | 0.13 | 0.05 | 0.16 | 1.97 | 0.08 | 0.06 | 0.12 | 0.29 | 0.50 | -1.06 |
| FFA 9:0 | 0.25 | 0.21 | 0.31 | 0.18 | 0.37 | -1.36 | 0.25 | 0.21 | 0.30 | 0.06 | 0.19 | -1.86 | 0.26 | 0.21 | 0.30 | 0.00 | 0.01 | -3.50 | 0.25 | 0.21 | 0.30 | 0.02 | 0.11 | -2.26 |
| FFA C22:1 | 2.04 | 1.44 | 2.81 | 0.34 | 0.55 | 0.95 | 2.13 | 1.36 | 2.74 | 0.17 | 0.36 | 1.38 | 2.07 | 1.45 | 2.75 | 0.20 | 0.38 | 1.29 | 2.07 | 1.30 | 2.82 | 0.64 | 0.78 | 0.47 |
| ffa:oeratio | 0.01 | 0.01 | 0.01 | 0.06 | 0.18 | 1.89 | 0.01 | 0.01 | 0.01 | 0.01 | 0.07 | 2.47 | 0.01 | 0.01 | 0.01 | 0.45 | 0.65 | 0.76 | 0.01 | 0.01 | 0.01 | 0.01 | 0.04 | 2.72 |
| FFAe | 328.09 | 226.87 | 381.18 | 0.07 | 0.20 | -1.83 | 339.74 | 287.83 | 376.33 | 0.05 | 0.16 | -1.97 | 318.86 | 283.04 | 366.00 | 0.05 | 0.17 | -1.92 | 305.40 | 277.98 | 354.37 | 0.00 | 0.00 | -3.79 |
| FFAo | 2.45 | 1.96 | 2.80 | 0.04 | 0.14 | -2.08 | 2.53 | 2.16 | 2.84 | 0.06 | 0.20 | -1.85 | 2.36 | 2.21 | 2.62 | 0.01 | 0.06 | -2.56 | 2.38 | 2.18 | 2.57 | 0.00 | 0.00 | -4.21 |
| FFAs | 330.57 | 228.62 | 383.99 | 0.07 | 0.20 | -1.82 | 342.35 | 289.70 | 379.08 | 0.05 | 0.16 | -1.98 | 321.32 | 285.28 | 368.27 | 0.05 | 0.17 | -1.94 | 307.96 | 280.23 | 357.09 | 0.00 | 0.00 | -3.79 |
| Hexadecadienoylcarnitine C16:2 | 0.01 | 0.01 | 0.02 | 0.21 | 0.40 | -1.25 | 0.01 | 0.01 | 0.02 | 0.72 | 0.84 | -0.36 | 0.01 | 0.01 | 0.02 | 0.03 | 0.13 | -2.13 | 0.01 | 0.01 | 0.02 | 0.00 | 0.03 | -2.86 |
| Hexadecenoylcarnitine C16:1 | 0.02 | 0.02 | 0.03 | 0.73 | 0.84 | 0.34 | 0.02 | 0.02 | 0.03 | 0.71 | 0.84 | 0.37 | 0.02 | 0.01 | 0.03 | 0.48 | 0.68 | -0.71 | 0.02 | 0.02 | 0.03 | 0.01 | 0.05 | -2.66 |
| Hexanoylcarnitine(C6) | 0.02 | 0.02 | 0.03 | 0.18 | 0.37 | -1.36 | 0.02 | 0.02 | 0.03 | 0.39 | 0.59 | -0.86 | 0.02 | 0.01 | 0.03 | 0.02 | 0.08 | -2.40 | 0.02 | 0.01 | 0.03 | 0.00 | 0.01 | -3.37 |
| L-Acetylcarnitine(C2) | 0.16 | 0.09 | 0.22 | 0.00 | 0.01 | -3.30 | 0.16 | 0.09 | 0.21 | 0.30 | 0.50 | -1.05 | 0.15 | 0.10 | 0.20 | 0.04 | 0.16 | -2.01 | 0.15 | 0.10 | 0.21 | 0.07 | 0.20 | -1.83 |
| L-Carnitine | 0.26 | 0.17 | 0.29 | 0.03 | 0.13 | -2.12 | 0.25 | 0.18 | 0.30 | 0.66 | 0.80 | -0.44 | 0.24 | 0.17 | 0.28 | 0.02 | 0.10 | -2.30 | 0.25 | 0.18 | 0.29 | 0.00 | 0.00 | -3.53 |
| linolenyl carnitine C18:3 | 0.01 | 0.01 | 0.01 | 0.75 | 0.86 | -0.32 | 0.01 | 0.01 | 0.01 | 0.85 | 0.91 | -0.19 | 0.01 | 0.00 | 0.01 | 0.63 | 0.78 | -0.48 | 0.01 | 0.01 | 0.01 | 0.13 | 0.31 | -1.50 |
| Linoleyl carnitine C18:2 | 0.10 | 0.08 | 0.13 | 0.62 | 0.78 | -0.49 | 0.11 | 0.09 | 0.13 | 0.73 | 0.84 | 0.34 | 0.10 | 0.08 | 0.12 | 0.56 | 0.74 | -0.58 | 0.11 | 0.08 | 0.14 | 0.86 | 0.92 | 0.18 |
| LPC 14:0 sn-1 | 0.39 | 0.28 | 0.54 | 0.19 | 0.38 | 1.31 | 0.39 | 0.27 | 0.53 | 0.58 | 0.75 | 0.55 | 0.38 | 0.27 | 0.56 | 0.33 | 0.53 | 0.98 | 0.34 | 0.28 | 0.47 | 0.19 | 0.38 | -1.30 |
| LPC 14:0 sn-2 | 0.03 | 0.02 | 0.04 | 0.36 | 0.56 | 0.92 | 0.03 | 0.02 | 0.04 | 0.94 | 0.96 | -0.08 | 0.03 | 0.02 | 0.04 | 0.54 | 0.73 | 0.61 | 0.02 | 0.02 | 0.04 | 0.09 | 0.24 | -1.69 |
| LPC 15:0 sn-1 | 0.12 | 0.09 | 0.15 | 0.03 | 0.12 | 2.18 | 0.11 | 0.09 | 0.14 | 0.85 | 0.91 | -0.19 | 0.12 | 0.09 | 0.15 | 0.08 | 0.22 | 1.77 | 0.11 | 0.09 | 0.15 | 0.69 | 0.82 | 0.40 |
| LPC 15:0 sn-2 | 0.01 | 0.01 | 0.01 | 0.81 | 0.90 | 0.24 | 0.01 | 0.01 | 0.01 | 0.18 | 0.37 | -1.34 | 0.01 | 0.01 | 0.01 | 0.97 | 0.98 | -0.04 | 0.01 | 0.01 | 0.01 | 0.30 | 0.50 | -1.04 |
| LPC 16:0 sn-1 | 16.72 | 13.93 | 19.98 | 0.46 | 0.67 | 0.73 | 17.41 | 14.11 | 20.17 | 0.94 | 0.96 | 0.08 | 16.36 | 13.82 | 18.92 | 0.92 | 0.95 | -0.10 | 16.18 | 13.94 | 18.87 | 0.05 | 0.17 | -1.93 |
| LPC 16:0 sn-2 | 1.84 | 1.40 | 2.28 | 0.83 | 0.91 | 0.22 | 1.86 | 1.57 | 2.35 | 0.91 | 0.95 | -0.11 | 1.80 | 1.41 | 2.13 | 0.76 | 0.87 | -0.30 | 1.78 | 1.52 | 2.04 | 0.00 | 0.03 | -2.83 |
| LPC 16:1 sn-1 | 0.67 | 0.53 | 0.85 | 0.19 | 0.38 | 1.30 | 0.68 | 0.53 | 0.87 | 0.95 | 0.97 | 0.06 | 0.70 | 0.53 | 0.98 | 0.25 | 0.45 | 1.15 | 0.65 | 0.51 | 0.81 | 0.29 | 0.50 | -1.06 |
| LPC 16:1 sn-2 | 0.04 | 0.03 | 0.06 | 0.59 | 0.75 | 0.54 | 0.04 | 0.03 | 0.06 | 0.68 | 0.81 | 0.41 | 0.04 | 0.03 | 0.07 | 0.52 | 0.71 | 0.64 | 0.04 | 0.03 | 0.05 | 0.10 | 0.26 | -1.63 |
| LPC 17:0 | 0.21 | 0.17 | 0.27 | 0.78 | 0.87 | -0.28 | 0.21 | 0.18 | 0.29 | 0.53 | 0.72 | -0.62 | 0.21 | 0.16 | 0.25 | 0.04 | 0.16 | -2.01 | 0.20 | 0.16 | 0.26 | 0.00 | 0.01 | -3.39 |
| LPC 18:0 sn-1 | 5.96 | 5.20 | 7.49 | 0.64 | 0.79 | 0.46 | 6.49 | 5.42 | 7.61 | 0.55 | 0.73 | -0.60 | 5.75 | 4.84 | 6.63 | 0.01 | 0.06 | -2.58 | 5.86 | 4.92 | 6.83 | 0.00 | 0.00 | -3.75 |
| LPC 18:0 sn-2 | 0.36 | 0.29 | 0.45 | 0.91 | 0.95 | 0.11 | 0.37 | 0.31 | 0.44 | 0.25 | 0.46 | -1.14 | 0.33 | 0.26 | 0.39 | 0.00 | 0.03 | -2.92 | 0.34 | 0.27 | 0.40 | 0.00 | 0.00 | -4.30 |
| LPC 18:1 sn-1 | 3.53 | 3.01 | 4.44 | 0.07 | 0.20 | 1.84 | 3.57 | 3.02 | 4.40 | 0.45 | 0.65 | 0.75 | 3.67 | 2.89 | 4.58 | 0.03 | 0.13 | 2.12 | 3.53 | 2.99 | 4.43 | 0.64 | 0.79 | -0.46 |
| LPC 18:1 sn-2 | 0.22 | 0.17 | 0.28 | 0.28 | 0.48 | 1.09 | 0.22 | 0.18 | 0.29 | 0.15 | 0.33 | 1.45 | 0.22 | 0.18 | 0.31 | 0.04 | 0.15 | 2.04 | 0.22 | 0.18 | 0.28 | 0.55 | 0.73 | -0.60 |
| LPC 18:2 sn-1 | 9.71 | 7.37 | 11.78 | 0.12 | 0.29 | 1.55 | 10.14 | 7.85 | 12.19 | 0.03 | 0.13 | 2.12 | 9.09 | 7.26 | 11.16 | 0.18 | 0.37 | 1.35 | 9.15 | 7.83 | 11.82 | 0.49 | 0.69 | 0.69 |
| LPC 18:2 sn-2 | 0.83 | 0.59 | 1.09 | 0.40 | 0.61 | 0.84 | 0.86 | 0.63 | 1.15 | 0.09 | 0.24 | 1.70 | 0.76 | 0.59 | 1.02 | 0.43 | 0.63 | 0.79 | 0.79 | 0.63 | 1.09 | 0.72 | 0.84 | -0.36 |
| LPC 18:3 sn-1 | 0.93 | 0.72 | 1.29 | 0.37 | 0.57 | 0.90 | 0.96 | 0.77 | 1.32 | 0.19 | 0.38 | 1.30 | 0.89 | 0.70 | 1.22 | 0.62 | 0.78 | 0.50 | 0.93 | 0.73 | 1.16 | 0.70 | 0.82 | -0.39 |
| LPC 18:3 sn-2 | 0.12 | 0.08 | 0.18 | 0.72 | 0.84 | 0.36 | 0.12 | 0.08 | 0.18 | 0.59 | 0.75 | 0.54 | 0.13 | 0.08 | 0.17 | 0.82 | 0.90 | 0.22 | 0.12 | 0.08 | 0.16 | 0.13 | 0.31 | -1.50 |
| LPC 20:0 sn-1 | 0.02 | 0.02 | 0.03 | 0.11 | 0.26 | 1.62 | 0.03 | 0.02 | 0.03 | 0.78 | 0.88 | -0.27 | 0.02 | 0.02 | 0.03 | 0.30 | 0.50 | -1.03 | 0.02 | 0.02 | 0.03 | 0.14 | 0.31 | -1.48 |
| LPC 20:0 sn-2 | 0.00 | 0.00 | 0.00 | 0.29 | 0.50 | 1.06 | 0.00 | 0.00 | 0.00 | 0.56 | 0.73 | 0.58 | 0.00 | 0.00 | 0.00 | 0.31 | 0.52 | -1.01 | 0.00 | 0.00 | 0.00 | 0.20 | 0.38 | -1.28 |
| LPC 20:1 sn-1 | 0.04 | 0.03 | 0.05 | 0.06 | 0.18 | 1.89 | 0.04 | 0.03 | 0.05 | 0.57 | 0.74 | 0.57 | 0.04 | 0.03 | 0.05 | 0.20 | 0.38 | -1.27 | 0.04 | 0.03 | 0.05 | 0.00 | 0.01 | -3.30 |
| LPC 20:1 sn-2 | 0.00 | 0.00 | 0.00 | 0.07 | 0.20 | 1.82 | 0.00 | 0.00 | 0.00 | 0.37 | 0.57 | 0.90 | 0.00 | 0.00 | 0.00 | 0.50 | 0.70 | -0.68 | 0.00 | 0.00 | 0.00 | 0.00 | 0.00 | -3.52 |
| LPC 20:2 sn-1 | 0.05 | 0.04 | 0.07 | 0.01 | 0.05 | 2.60 | 0.06 | 0.05 | 0.07 | 0.87 | 0.92 | 0.16 | 0.05 | 0.04 | 0.06 | 0.15 | 0.33 | -1.46 | 0.05 | 0.04 | 0.07 | 0.42 | 0.62 | -0.81 |
| LPC 20:2 sn-2 | 0.00 | 0.00 | 0.00 | 0.10 | 0.26 | 1.65 | 0.00 | 0.00 | 0.00 | 0.99 | 0.99 | -0.01 | 0.00 | 0.00 | 0.00 | 0.11 | 0.28 | -1.59 | 0.00 | 0.00 | 0.00 | 0.00 | 0.03 | -2.90 |
| LPC 20:3 sn-1 | 0.43 | 0.33 | 0.57 | 0.02 | 0.10 | 2.29 | 0.42 | 0.31 | 0.59 | 0.13 | 0.30 | 1.53 | 0.39 | 0.27 | 0.53 | 0.93 | 0.96 | 0.09 | 0.41 | 0.32 | 0.52 | 0.42 | 0.62 | -0.81 |
| LPC 20:4 sn-1 | 2.68 | 1.94 | 3.71 | 0.05 | 0.16 | 1.97 | 2.57 | 1.99 | 3.52 | 0.25 | 0.44 | 1.16 | 2.34 | 1.84 | 3.04 | 0.95 | 0.97 | 0.07 | 2.27 | 1.82 | 2.97 | 0.21 | 0.39 | -1.26 |
| LPC 20:5 | 0.22 | 0.13 | 0.40 | 0.07 | 0.21 | 1.78 | 0.23 | 0.13 | 0.37 | 0.39 | 0.59 | 0.86 | 0.22 | 0.10 | 0.39 | 0.61 | 0.77 | 0.51 | 0.16 | 0.10 | 0.28 | 0.04 | 0.14 | -2.08 |
| LPC 22:0 | 0.01 | 0.01 | 0.01 | 0.09 | 0.25 | 1.68 | 0.01 | 0.01 | 0.01 | 0.77 | 0.87 | 0.29 | 0.01 | 0.00 | 0.01 | 0.53 | 0.71 | 0.63 | 0.01 | 0.01 | 0.01 | 0.18 | 0.37 | 1.33 |
| LPC 22:1 | 0.00 | 0.00 | 0.00 | 0.03 | 0.13 | 2.13 | 0.00 | 0.00 | 0.01 | 0.10 | 0.26 | 1.63 | 0.00 | 0.00 | 0.00 | 0.32 | 0.53 | 0.99 | 0.00 | 0.00 | 0.00 | 0.11 | 0.27 | -1.60 |
| LPC 22:4 | 0.02 | 0.01 | 0.03 | 0.03 | 0.11 | 2.23 | 0.02 | 0.01 | 0.03 | 0.07 | 0.21 | 1.79 | 0.02 | 0.01 | 0.02 | 0.51 | 0.71 | 0.65 | 0.02 | 0.01 | 0.02 | 0.73 | 0.84 | -0.35 |
| LPC 22:5 sn-1 | 0.08 | 0.06 | 0.11 | 0.01 | 0.05 | 2.64 | 0.08 | 0.06 | 0.10 | 0.19 | 0.38 | 1.31 | 0.07 | 0.06 | 0.09 | 0.64 | 0.78 | 0.47 | 0.07 | 0.06 | 0.10 | 0.98 | 0.99 | 0.02 |
| LPC 22:6 sn-1 | 0.64 | 0.47 | 1.03 | 0.07 | 0.20 | 1.83 | 0.68 | 0.50 | 0.92 | 0.86 | 0.92 | 0.18 | 0.63 | 0.42 | 0.89 | 0.86 | 0.92 | -0.18 | 0.64 | 0.49 | 0.94 | 0.08 | 0.23 | -1.74 |
| LPC 24:0 | 0.01 | 0.01 | 0.01 | 0.54 | 0.73 | 0.61 | 0.01 | 0.01 | 0.01 | 0.69 | 0.82 | 0.40 | 0.01 | 0.01 | 0.01 | 0.43 | 0.63 | -0.79 | 0.01 | 0.01 | 0.01 | 0.70 | 0.83 | -0.38 |
| LPC O-16:0 | 0.15 | 0.11 | 0.18 | 0.01 | 0.04 | 2.77 | 0.14 | 0.12 | 0.18 | 0.05 | 0.16 | 1.99 | 0.12 | 0.10 | 0.16 | 0.51 | 0.70 | -0.66 | 0.13 | 0.11 | 0.16 | 0.18 | 0.37 | -1.34 |
| LPC O-16:1 | 0.18 | 0.15 | 0.23 | 0.01 | 0.04 | 2.76 | 0.19 | 0.15 | 0.23 | 0.14 | 0.32 | 1.47 | 0.18 | 0.14 | 0.22 | 0.30 | 0.50 | 1.03 | 0.17 | 0.15 | 0.23 | 0.75 | 0.86 | -0.31 |
| LPC O-18:0 | 0.03 | 0.02 | 0.03 | 0.02 | 0.08 | 2.42 | 0.03 | 0.02 | 0.03 | 0.29 | 0.50 | 1.05 | 0.02 | 0.02 | 0.03 | 0.78 | 0.87 | -0.28 | 0.02 | 0.02 | 0.03 | 0.09 | 0.25 | -1.68 |
| LPC O-18:1 | 0.08 | 0.06 | 0.10 | 0.03 | 0.13 | 2.15 | 0.08 | 0.06 | 0.09 | 0.41 | 0.61 | 0.83 | 0.07 | 0.06 | 0.09 | 0.16 | 0.35 | -1.40 | 0.07 | 0.06 | 0.09 | 0.01 | 0.04 | -2.71 |
| LPC P-18:0 | 0.01 | 0.01 | 0.02 | 0.28 | 0.48 | 1.09 | 0.02 | 0.01 | 0.02 | 0.52 | 0.71 | 0.64 | 0.01 | 0.01 | 0.02 | 0.66 | 0.80 | 0.44 | 0.01 | 0.01 | 0.02 | 0.66 | 0.80 | -0.44 |
| LPC P-18:1 | 0.01 | 0.01 | 0.01 | 0.02 | 0.08 | 2.39 | 0.01 | 0.01 | 0.02 | 0.45 | 0.66 | 0.75 | 0.01 | 0.01 | 0.01 | 0.75 | 0.86 | -0.32 | 0.01 | 0.01 | 0.02 | 0.89 | 0.94 | -0.14 |
| LPCS | 47.52 | 37.76 | 55.14 | 0.14 | 0.31 | 1.49 | 47.98 | 40.54 | 58.13 | 0.51 | 0.70 | 0.67 | 43.83 | 37.24 | 53.20 | 0.72 | 0.84 | 0.36 | 45.05 | 39.20 | 52.65 | 0.10 | 0.26 | -1.65 |
| lpcs:pc:ratio | 0.58 | 0.52 | 0.64 | 0.31 | 0.52 | 1.01 | 0.59 | 0.52 | 0.67 | 0.99 | 0.99 | 0.01 | 0.58 | 0.51 | 0.64 | 0.23 | 0.42 | 1.20 | 0.58 | 0.53 | 0.66 | 0.50 | 0.70 | 0.67 |
| LPE 16:0 | 0.18 | 0.15 | 0.21 | 0.07 | 0.20 | 1.83 | 0.18 | 0.14 | 0.22 | 0.92 | 0.95 | 0.10 | 0.17 | 0.14 | 0.22 | 0.71 | 0.83 | 0.37 | 0.17 | 0.13 | 0.21 | 0.57 | 0.74 | -0.57 |
| LPE 16:1 | 0.01 | 0.01 | 0.01 | 0.35 | 0.56 | 0.93 | 0.01 | 0.00 | 0.01 | 0.64 | 0.78 | -0.47 | 0.01 | 0.00 | 0.01 | 0.95 | 0.97 | -0.07 | 0.01 | 0.00 | 0.01 | 0.26 | 0.47 | -1.12 |
| LPE 18:0 sn-1 | 0.19 | 0.16 | 0.23 | 0.06 | 0.19 | 1.87 | 0.19 | 0.16 | 0.24 | 0.45 | 0.65 | -0.75 | 0.17 | 0.15 | 0.22 | 0.05 | 0.17 | -1.96 | 0.18 | 0.15 | 0.22 | 0.00 | 0.01 | -3.24 |
| LPE 18:0 sn-2 | 0.01 | 0.01 | 0.01 | 0.34 | 0.55 | 0.96 | 0.01 | 0.01 | 0.01 | 0.83 | 0.91 | -0.21 | 0.01 | 0.01 | 0.01 | 0.03 | 0.13 | -2.11 | 0.01 | 0.01 | 0.01 | 0.00 | 0.00 | -4.33 |
| LPE 18:1 sn-1 | 0.13 | 0.10 | 0.16 | 0.20 | 0.38 | 1.28 | 0.12 | 0.10 | 0.15 | 0.41 | 0.61 | -0.83 | 0.13 | 0.10 | 0.17 | 0.01 | 0.04 | 2.77 | 0.13 | 0.10 | 0.16 | 0.19 | 0.38 | 1.31 |
| LPE 18:1 sn-2 | 0.01 | 0.01 | 0.01 | 0.66 | 0.80 | 0.43 | 0.01 | 0.01 | 0.01 | 0.59 | 0.75 | -0.54 | 0.01 | 0.01 | 0.01 | 0.04 | 0.15 | 2.06 | 0.01 | 0.01 | 0.01 | 0.49 | 0.69 | -0.69 |
| LPE 18:2 sn-1 | 0.48 | 0.38 | 0.64 | 0.38 | 0.59 | 0.87 | 0.50 | 0.39 | 0.66 | 0.29 | 0.50 | 1.06 | 0.49 | 0.38 | 0.71 | 0.02 | 0.10 | 2.30 | 0.47 | 0.39 | 0.66 | 0.17 | 0.35 | 1.39 |
| LPE 18:2 sn-2 | 0.04 | 0.03 | 0.05 | 0.66 | 0.80 | -0.44 | 0.04 | 0.03 | 0.06 | 0.90 | 0.95 | 0.12 | 0.04 | 0.03 | 0.06 | 0.33 | 0.54 | 0.97 | 0.04 | 0.03 | 0.05 | 0.83 | 0.91 | -0.22 |
| LPE 18:3sn-1 | 0.00 | 0.00 | 0.01 | 0.10 | 0.26 | 1.63 | 0.00 | 0.00 | 0.01 | 0.52 | 0.71 | 0.64 | 0.00 | 0.00 | 0.01 | 0.18 | 0.37 | 1.35 | 0.00 | 0.00 | 0.01 | 0.52 | 0.71 | -0.64 |
| LPE 20:3 | 0.02 | 0.01 | 0.02 | 0.02 | 0.09 | 2.34 | 0.02 | 0.01 | 0.02 | 0.36 | 0.56 | 0.92 | 0.02 | 0.01 | 0.02 | 0.30 | 0.50 | 1.03 | 0.02 | 0.01 | 0.02 | 0.38 | 0.58 | 0.88 |
| LPE 20:4 sn-1 | 0.36 | 0.24 | 0.49 | 0.06 | 0.18 | 1.90 | 0.34 | 0.26 | 0.48 | 0.13 | 0.30 | 1.52 | 0.35 | 0.24 | 0.46 | 0.16 | 0.35 | 1.41 | 0.33 | 0.25 | 0.44 | 0.78 | 0.87 | 0.29 |
| LPE 20:4 sn-2 | 0.03 | 0.02 | 0.05 | 0.68 | 0.81 | 0.41 | 0.03 | 0.02 | 0.04 | 0.56 | 0.73 | 0.58 | 0.03 | 0.02 | 0.04 | 0.69 | 0.82 | 0.39 | 0.03 | 0.02 | 0.04 | 0.09 | 0.24 | -1.72 |
| LPE 20:5 sn-1 | 0.02 | 0.01 | 0.03 | 0.06 | 0.19 | 1.87 | 0.02 | 0.01 | 0.03 | 0.16 | 0.35 | 1.39 | 0.02 | 0.01 | 0.04 | 0.27 | 0.48 | 1.09 | 0.01 | 0.01 | 0.03 | 0.47 | 0.68 | -0.72 |
| LPE 22:4 sn-1 | 0.02 | 0.01 | 0.02 | 0.20 | 0.38 | 1.28 | 0.02 | 0.01 | 0.02 | 0.54 | 0.73 | 0.61 | 0.01 | 0.01 | 0.02 | 0.81 | 0.90 | 0.24 | 0.01 | 0.01 | 0.02 | 0.74 | 0.85 | -0.33 |
| LPE 22:6 sn-1 | 0.28 | 0.20 | 0.40 | 0.12 | 0.29 | 1.55 | 0.27 | 0.20 | 0.37 | 0.55 | 0.73 | 0.59 | 0.28 | 0.19 | 0.38 | 0.57 | 0.74 | 0.57 | 0.25 | 0.19 | 0.36 | 0.30 | 0.50 | -1.05 |
| Octanoylcarnitine(C8) | 0.07 | 0.05 | 0.10 | 0.83 | 0.91 | -0.21 | 0.07 | 0.05 | 0.09 | 0.58 | 0.74 | -0.56 | 0.05 | 0.04 | 0.07 | 0.02 | 0.09 | -2.34 | 0.06 | 0.04 | 0.07 | 0.00 | 0.00 | -3.61 |
| Oleoylcarnitine C18:1 | 0.08 | 0.06 | 0.10 | 0.15 | 0.33 | -1.45 | 0.08 | 0.06 | 0.10 | 0.07 | 0.20 | -1.81 | 0.07 | 0.06 | 0.09 | 0.00 | 0.01 | -3.42 | 0.07 | 0.06 | 0.09 | 0.00 | 0.00 | -4.16 |
| Palmitoylcarnitine C16 | 0.08 | 0.07 | 0.09 | 0.73 | 0.84 | -0.35 | 0.07 | 0.06 | 0.09 | 0.92 | 0.95 | -0.10 | 0.07 | 0.06 | 0.09 | 0.87 | 0.92 | 0.17 | 0.07 | 0.06 | 0.09 | 0.64 | 0.78 | -0.47 |
| PC 16:0:18:2 | 18.15 | 14.75 | 24.17 | 0.99 | 0.99 | -0.01 | 18.94 | 15.09 | 24.39 | 0.40 | 0.60 | 0.84 | 17.39 | 14.24 | 21.83 | 0.51 | 0.70 | -0.67 | 18.39 | 15.16 | 22.67 | 0.14 | 0.32 | -1.47 |
| PC 30:0 | 0.16 | 0.11 | 0.22 | 0.53 | 0.72 | 0.62 | 0.15 | 0.10 | 0.24 | 0.33 | 0.54 | 0.96 | 0.15 | 0.10 | 0.21 | 0.18 | 0.37 | -1.33 | 0.12 | 0.10 | 0.18 | 0.00 | 0.02 | -3.09 |
| PC 32:1 | 0.95 | 0.67 | 1.22 | 0.99 | 0.99 | -0.01 | 0.85 | 0.57 | 1.13 | 0.44 | 0.64 | -0.77 | 0.84 | 0.56 | 1.16 | 0.20 | 0.38 | -1.29 | 0.76 | 0.54 | 1.08 | 0.01 | 0.05 | -2.61 |
| PC 32:2 | 0.92 | 0.72 | 1.24 | 0.27 | 0.48 | -1.10 | 1.01 | 0.72 | 1.27 | 0.66 | 0.80 | -0.45 | 0.85 | 0.66 | 1.22 | 0.05 | 0.16 | -2.00 | 0.90 | 0.70 | 1.14 | 0.00 | 0.02 | -2.95 |
| PC 33:1 | 0.11 | 0.09 | 0.15 | 0.18 | 0.37 | 1.34 | 0.11 | 0.08 | 0.15 | 0.82 | 0.90 | -0.23 | 0.11 | 0.09 | 0.15 | 0.89 | 0.94 | -0.13 | 0.11 | 0.08 | 0.14 | 0.05 | 0.16 | -1.96 |
| PC 34:0 | 1.43 | 1.13 | 1.74 | 0.73 | 0.84 | 0.35 | 1.43 | 1.14 | 1.75 | 0.67 | 0.81 | 0.42 | 1.33 | 1.09 | 1.65 | 0.18 | 0.37 | -1.33 | 1.23 | 1.06 | 1.64 | 0.04 | 0.15 | -2.04 |
| PC 34:1 | 10.01 | 7.77 | 13.01 | 0.57 | 0.74 | 0.57 | 10.46 | 7.75 | 13.10 | 0.52 | 0.71 | 0.64 | 9.69 | 7.89 | 12.72 | 0.88 | 0.93 | -0.15 | 9.31 | 7.97 | 12.31 | 0.16 | 0.35 | -1.41 |
| PC 34:3 | 2.04 | 1.71 | 2.44 | 0.05 | 0.16 | -1.97 | 2.03 | 1.49 | 2.45 | 0.04 | 0.15 | -2.06 | 2.03 | 1.60 | 2.61 | 0.18 | 0.37 | -1.34 | 1.87 | 1.64 | 2.34 | 0.01 | 0.05 | -2.67 |
| PC 34:4 | 0.23 | 0.15 | 0.35 | 0.92 | 0.95 | -0.10 | 0.22 | 0.15 | 0.32 | 0.10 | 0.26 | -1.65 | 0.21 | 0.16 | 0.32 | 0.10 | 0.26 | -1.65 | 0.20 | 0.14 | 0.28 | 0.00 | 0.01 | -3.33 |
| PC 35:2 | 0.80 | 0.63 | 1.03 | 0.24 | 0.44 | -1.17 | 0.80 | 0.69 | 0.97 | 0.41 | 0.61 | -0.83 | 0.79 | 0.63 | 0.96 | 0.02 | 0.08 | -2.41 | 0.83 | 0.63 | 1.03 | 0.00 | 0.03 | -2.81 |
| PC 35:3 | 0.13 | 0.10 | 0.16 | 0.01 | 0.08 | 2.43 | 0.12 | 0.10 | 0.16 | 0.12 | 0.29 | 1.56 | 0.12 | 0.09 | 0.16 | 0.10 | 0.26 | 1.65 | 0.13 | 0.10 | 0.15 | 0.13 | 0.30 | 1.53 |
| PC 36:2 | 11.73 | 9.69 | 14.66 | 0.66 | 0.80 | 0.45 | 11.73 | 9.74 | 15.24 | 0.29 | 0.50 | 1.06 | 10.80 | 9.26 | 13.99 | 0.40 | 0.61 | -0.84 | 11.13 | 9.25 | 13.99 | 0.12 | 0.29 | -1.56 |
| PC 36:3 | 8.06 | 6.60 | 9.35 | 0.80 | 0.88 | 0.26 | 8.22 | 6.63 | 9.84 | 0.38 | 0.59 | 0.87 | 7.63 | 6.26 | 9.44 | 0.35 | 0.56 | -0.93 | 7.86 | 6.68 | 9.53 | 0.35 | 0.55 | -0.94 |
| PC 36:4 | 6.40 | 4.44 | 8.71 | 0.52 | 0.71 | 0.64 | 6.04 | 4.45 | 8.47 | 0.30 | 0.50 | 1.03 | 6.01 | 4.32 | 8.00 | 0.64 | 0.79 | -0.46 | 5.84 | 4.39 | 7.69 | 0.10 | 0.26 | -1.64 |
| PC 36:5 | 2.12 | 1.47 | 3.02 | 0.98 | 0.99 | 0.03 | 2.11 | 1.41 | 3.20 | 0.92 | 0.95 | 0.10 | 2.09 | 1.23 | 3.28 | 0.10 | 0.26 | -1.65 | 1.68 | 1.16 | 2.34 | 0.00 | 0.00 | -4.21 |
| PC 36:6 | 0.07 | 0.05 | 0.09 | 0.64 | 0.78 | -0.47 | 0.07 | 0.04 | 0.11 | 0.32 | 0.52 | -1.00 | 0.07 | 0.04 | 0.11 | 0.04 | 0.15 | -2.03 | 0.06 | 0.04 | 0.10 | 0.00 | 0.00 | -3.57 |
| PC 38:4 | 4.48 | 3.53 | 5.90 | 0.17 | 0.36 | 1.37 | 4.29 | 3.35 | 5.93 | 0.16 | 0.35 | 1.40 | 4.22 | 3.32 | 5.61 | 0.83 | 0.91 | -0.21 | 4.01 | 3.18 | 5.28 | 0.26 | 0.47 | -1.12 |
| PC 38:5 | 1.96 | 1.45 | 2.93 | 0.34 | 0.55 | 0.95 | 2.01 | 1.48 | 3.01 | 0.24 | 0.44 | 1.16 | 2.06 | 1.48 | 2.96 | 0.96 | 0.98 | -0.05 | 1.90 | 1.43 | 2.64 | 0.35 | 0.55 | -0.94 |
| PC 38:6 | 7.61 | 6.52 | 10.34 | 0.05 | 0.17 | 1.95 | 8.12 | 6.70 | 9.79 | 0.20 | 0.38 | 1.28 | 7.55 | 6.08 | 9.85 | 0.91 | 0.95 | -0.12 | 7.73 | 6.41 | 10.37 | 0.20 | 0.38 | -1.28 |
| PC 38:7 | 0.66 | 0.48 | 0.80 | 0.85 | 0.91 | 0.19 | 0.65 | 0.52 | 0.89 | 0.04 | 0.13 | 2.11 | 0.62 | 0.47 | 0.78 | 0.81 | 0.90 | 0.24 | 0.64 | 0.49 | 0.82 | 0.98 | 0.99 | -0.03 |
| PC O-34:2 | 0.19 | 0.14 | 0.24 | 0.92 | 0.95 | -0.10 | 0.18 | 0.14 | 0.25 | 0.36 | 0.57 | 0.91 | 0.18 | 0.14 | 0.23 | 0.68 | 0.81 | -0.42 | 0.18 | 0.14 | 0.22 | 0.05 | 0.16 | -2.00 |
| PC O-36:4 | 1.40 | 1.07 | 1.76 | 0.95 | 0.97 | -0.07 | 1.42 | 1.13 | 1.79 | 0.17 | 0.35 | 1.38 | 1.20 | 0.99 | 1.65 | 0.38 | 0.58 | -0.88 | 1.30 | 1.03 | 1.58 | 0.05 | 0.17 | -1.95 |
| PC O-36:5 | 0.03 | 0.02 | 0.04 | 0.36 | 0.57 | 0.91 | 0.03 | 0.02 | 0.04 | 0.07 | 0.20 | 1.82 | 0.03 | 0.02 | 0.04 | 0.40 | 0.60 | 0.84 | 0.03 | 0.02 | 0.04 | 0.71 | 0.83 | 0.37 |
| PC O-38:5 | 1.25 | 1.07 | 1.60 | 0.73 | 0.84 | 0.35 | 1.28 | 1.08 | 1.62 | 0.18 | 0.37 | 1.33 | 1.18 | 0.98 | 1.46 | 0.09 | 0.24 | -1.70 | 1.16 | 0.98 | 1.48 | 0.00 | 0.02 | -3.10 |
| PC O-38:6 | 0.30 | 0.23 | 0.37 | 0.89 | 0.94 | -0.14 | 0.30 | 0.25 | 0.38 | 0.23 | 0.42 | 1.21 | 0.27 | 0.22 | 0.36 | 0.18 | 0.37 | -1.35 | 0.28 | 0.21 | 0.34 | 0.03 | 0.11 | -2.24 |
| PCS | 78.64 | 64.79 | 99.83 | 0.70 | 0.83 | 0.38 | 79.34 | 65.54 | 97.84 | 0.46 | 0.66 | 0.74 | 76.81 | 62.44 | 95.08 | 0.46 | 0.66 | -0.75 | 75.46 | 64.02 | 93.66 | 0.10 | 0.26 | -1.63 |
| PE 34:2 | 0.07 | 0.05 | 0.09 | 0.59 | 0.75 | 0.54 | 0.07 | 0.04 | 0.10 | 0.58 | 0.75 | 0.55 | 0.06 | 0.04 | 0.08 | 0.02 | 0.09 | -2.34 | 0.06 | 0.04 | 0.09 | 0.12 | 0.29 | -1.55 |
| PE 38:6 | 0.16 | 0.12 | 0.22 | 0.29 | 0.50 | -1.05 | 0.15 | 0.11 | 0.23 | 0.04 | 0.15 | -2.03 | 0.13 | 0.10 | 0.17 | 0.00 | 0.00 | -4.57 | 0.13 | 0.08 | 0.20 | 0.00 | 0.00 | -4.58 |
| PE O-34:3 | 0.06 | 0.05 | 0.09 | 0.60 | 0.76 | -0.53 | 0.07 | 0.05 | 0.10 | 0.30 | 0.50 | 1.04 | 0.06 | 0.05 | 0.08 | 0.07 | 0.20 | -1.82 | 0.06 | 0.04 | 0.08 | 0.07 | 0.20 | -1.84 |
| PE O-36:5 | 0.28 | 0.20 | 0.37 | 0.41 | 0.61 | 0.82 | 0.28 | 0.21 | 0.39 | 0.38 | 0.59 | 0.87 | 0.24 | 0.18 | 0.32 | 0.31 | 0.52 | -1.01 | 0.25 | 0.18 | 0.31 | 0.01 | 0.04 | -2.74 |
| PE O-38:6 | 0.19 | 0.14 | 0.24 | 0.14 | 0.33 | -1.46 | 0.19 | 0.14 | 0.26 | 0.88 | 0.93 | 0.16 | 0.15 | 0.12 | 0.23 | 0.01 | 0.06 | -2.57 | 0.17 | 0.12 | 0.21 | 0.00 | 0.00 | -4.22 |
| PE O-38:7 | 0.13 | 0.10 | 0.17 | 0.50 | 0.70 | -0.67 | 0.14 | 0.10 | 0.18 | 0.49 | 0.69 | 0.69 | 0.11 | 0.08 | 0.16 | 0.13 | 0.30 | -1.53 | 0.11 | 0.09 | 0.16 | 0.00 | 0.00 | -4.09 |
| Propionyl-L-carnitine(C3) | 0.02 | 0.02 | 0.03 | 0.01 | 0.06 | 2.54 | 0.02 | 0.02 | 0.03 | 0.00 | 0.00 | 3.78 | 0.02 | 0.02 | 0.03 | 0.02 | 0.10 | 2.28 | 0.02 | 0.02 | 0.03 | 0.00 | 0.00 | 3.89 |
| SM 32:1 | 0.72 | 0.59 | 0.87 | 0.00 | 0.02 | -3.01 | 0.72 | 0.57 | 0.99 | 0.09 | 0.24 | -1.70 | 0.70 | 0.50 | 0.91 | 0.00 | 0.03 | -2.90 | 0.67 | 0.51 | 0.80 | 0.00 | 0.01 | -3.49 |
| SM 32:2 | 0.05 | 0.04 | 0.05 | 0.13 | 0.30 | -1.52 | 0.05 | 0.04 | 0.06 | 0.32 | 0.52 | -0.99 | 0.04 | 0.03 | 0.05 | 0.00 | 0.01 | -3.25 | 0.04 | 0.03 | 0.05 | 0.00 | 0.00 | -4.99 |
| SM 33:1 | 0.40 | 0.32 | 0.49 | 0.30 | 0.50 | -1.04 | 0.41 | 0.34 | 0.50 | 0.29 | 0.50 | -1.07 | 0.40 | 0.29 | 0.48 | 0.15 | 0.33 | -1.45 | 0.37 | 0.31 | 0.48 | 0.00 | 0.01 | -3.13 |
| SM 33:2 | 0.01 | 0.01 | 0.01 | 0.05 | 0.16 | -1.98 | 0.01 | 0.01 | 0.01 | 0.11 | 0.28 | -1.58 | 0.01 | 0.01 | 0.01 | 0.00 | 0.01 | -3.45 | 0.01 | 0.01 | 0.01 | 0.00 | 0.00 | -3.64 |
| SM 34:1 | 10.31 | 8.82 | 12.70 | 0.50 | 0.70 | 0.67 | 10.43 | 9.04 | 12.78 | 0.21 | 0.39 | 1.26 | 10.07 | 8.14 | 11.80 | 0.25 | 0.46 | -1.14 | 9.53 | 8.59 | 11.80 | 0.06 | 0.18 | -1.90 |
| SM 34:2 | 2.60 | 2.29 | 3.01 | 0.00 | 0.02 | -3.08 | 2.58 | 2.28 | 3.03 | 0.02 | 0.08 | -2.40 | 2.34 | 2.03 | 2.77 | 0.00 | 0.00 | -4.92 | 2.51 | 2.12 | 2.74 | 0.00 | 0.00 | -5.06 |
| SM 35:2 | 0.03 | 0.03 | 0.04 | 0.08 | 0.23 | -1.73 | 0.03 | 0.03 | 0.04 | 0.09 | 0.25 | -1.69 | 0.03 | 0.02 | 0.03 | 0.00 | 0.00 | -4.37 | 0.03 | 0.02 | 0.04 | 0.00 | 0.00 | -4.84 |
| SM 36:1 | 1.56 | 1.25 | 1.94 | 0.08 | 0.23 | -1.73 | 1.52 | 1.29 | 1.86 | 0.36 | 0.57 | -0.91 | 1.38 | 1.06 | 1.74 | 0.00 | 0.02 | -3.06 | 1.35 | 1.11 | 1.76 | 0.00 | 0.01 | -3.42 |
| SM 36:2 | 1.16 | 0.98 | 1.40 | 0.00 | 0.02 | -3.01 | 1.14 | 0.92 | 1.36 | 0.03 | 0.11 | -2.21 | 0.98 | 0.83 | 1.14 | 0.00 | 0.00 | -5.01 | 1.00 | 0.82 | 1.27 | 0.00 | 0.00 | -5.43 |
| SM 36:3 | 0.06 | 0.05 | 0.07 | 0.00 | 0.00 | -4.82 | 0.06 | 0.05 | 0.07 | 0.00 | 0.02 | -3.10 | 0.06 | 0.05 | 0.07 | 0.00 | 0.00 | -4.76 | 0.06 | 0.05 | 0.07 | 0.00 | 0.00 | -5.11 |
| sm:pc | 0.18 | 0.16 | 0.20 | 0.55 | 0.73 | -0.60 | 0.18 | 0.16 | 0.20 | 0.48 | 0.68 | -0.70 | 0.17 | 0.15 | 0.19 | 0.20 | 0.38 | -1.28 | 0.17 | 0.16 | 0.19 | 0.23 | 0.43 | -1.19 |
| SMS | 16.82 | 14.95 | 20.79 | 0.82 | 0.90 | -0.23 | 17.45 | 14.92 | 20.27 | 0.74 | 0.85 | 0.33 | 15.95 | 13.50 | 18.77 | 0.03 | 0.12 | -2.19 | 15.66 | 14.01 | 19.05 | 0.00 | 0.02 | -2.97 |
| Sphingosine | 0.00 | 0.00 | 0.00 | 0.77 | 0.87 | -0.29 | 0.00 | 0.00 | 0.00 | 0.11 | 0.26 | -1.62 | 0.00 | 0.00 | 0.00 | 0.07 | 0.20 | -1.82 | 0.00 | 0.00 | 0.00 | 0.30 | 0.50 | -1.04 |
| Sphingosine-1-phosphate | 0.08 | 0.06 | 0.10 | 0.97 | 0.99 | 0.04 | 0.08 | 0.07 | 0.10 | 0.48 | 0.68 | -0.70 | 0.07 | 0.06 | 0.09 | 0.42 | 0.63 | -0.80 | 0.08 | 0.06 | 0.10 | 0.22 | 0.41 | -1.22 |
| Stearoylcarnitine C18 | 0.02 | 0.01 | 0.02 | 0.28 | 0.50 | 1.07 | 0.01 | 0.01 | 0.02 | 0.61 | 0.77 | 0.51 | 0.01 | 0.01 | 0.02 | 0.45 | 0.65 | 0.75 | 0.02 | 0.01 | 0.02 | 0.22 | 0.41 | 1.22 |
| Tetradecadiencarnitine C14:2 | 0.02 | 0.02 | 0.03 | 0.55 | 0.73 | -0.60 | 0.02 | 0.02 | 0.03 | 0.63 | 0.78 | 0.48 | 0.02 | 0.01 | 0.03 | 0.19 | 0.38 | -1.30 | 0.02 | 0.01 | 0.03 | 0.03 | 0.12 | -2.19 |
| Tetradecanoylcarnitine C14 | 0.01 | 0.00 | 0.01 | 0.12 | 0.29 | 1.55 | 0.00 | 0.00 | 0.01 | 0.42 | 0.62 | 0.81 | 0.00 | 0.00 | 0.01 | 0.28 | 0.48 | -1.09 | 0.00 | 0.00 | 0.01 | 0.37 | 0.57 | -0.90 |
| Tetradecenoylcarnitine C14:1 | 0.03 | 0.02 | 0.04 | 0.76 | 0.86 | -0.31 | 0.03 | 0.02 | 0.04 | 0.85 | 0.91 | 0.19 | 0.02 | 0.02 | 0.03 | 0.16 | 0.35 | -1.40 | 0.02 | 0.02 | 0.03 | 0.00 | 0.01 | -3.39 |
| Valerylcarnitine(C5) | 0.07 | 0.06 | 0.09 | 0.40 | 0.61 | 0.83 | 0.06 | 0.05 | 0.08 | 0.03 | 0.13 | 2.12 | 0.06 | 0.05 | 0.08 | 0.57 | 0.74 | -0.56 | 0.06 | 0.05 | 0.07 | 0.03 | 0.13 | -2.14 |
