# Supplementary material for: Combined berberine and probiotic treatment as an effective regimen for improving postprandial hyperlipidemia in type 2 diabetes patients: a double blinded placebo controlled randomized study
Source: Gut Microbes. 2021 Dec 20;14(1):2003176. doi: 10.1080/19490976.2021.2003176 (PMC8726654; doi:10.1080/19490976.2021.2003176)
Supplement: Supplemental Material [file KGMI_A_2003176_SM5262.zip › Supplementary information/Data Set 3.docx]

Data Set 3. The annotations of the 73 genes annotated as lipid metabolism-related genes in 9 ingested assembled probiotics genome.

| Genome ID | Species | Gene_id | Identity | E_value | Kegg_geneID | Ko_id | Ko_name | Ko_defi | Ko_EC | [PATH:Ko_class] Level1--Note |
| --- | --- | --- | --- | --- | --- | --- | --- | --- | --- | --- |
| BL88-Onlly | *Bifidobacterium longum* | GL001093 | 99.6 | 5.00E-149 | blb:BBMN68_1310 | K01048 | pldB | lysophospholipase | 3.1.1.5 | [PATH:ko00564] Level1--Metabolism \| Level2--Lipid metabolism \| Level3--Glycerophospholipid metabolism |
| BL88-Onlly | *Bifidobacterium longum* | GL000277 | 100 | 0 | blm:BLLJ_0763 | K01897 | ACSL, fadD | long-chain acyl-CoA synthetase | 6.2.1.3 | [PATH:ko02024] Level1--Cellular Processes \| Level2--Cellular community - prokaryotes \| Level3--Quorum sensing;[PATH:ko01100] Level1--Metabolism \| Level2--Global and overview maps \| Level3--Metabolic pathways;[PATH:ko00061] Level1--Metabolism \| Level2--Lipid metabolism \| Level3--Fatty acid biosynthesis;[PATH:ko01212] Level1--Metabolism \| Level2--Global and overview maps \| Level3--Fatty acid metabolism;[PATH:ko00071] Level1--Metabolism \| Level2--Lipid metabolism \| Level3--Fatty acid degradation |
| BL88-Onlly | *Bifidobacterium longum* | GL000808 | 100 | 0 | blm:BLLJ_1513 | K01897 | ACSL, fadD | long-chain acyl-CoA synthetase | 6.2.1.3 | [PATH:ko02024] Level1--Cellular Processes \| Level2--Cellular community - prokaryotes \| Level3--Quorum sensing;[PATH:ko01100] Level1--Metabolism \| Level2--Global and overview maps \| Level3--Metabolic pathways;[PATH:ko00061] Level1--Metabolism \| Level2--Lipid metabolism \| Level3--Fatty acid biosynthesis;[PATH:ko01212] Level1--Metabolism \| Level2--Global and overview maps \| Level3--Fatty acid metabolism;[PATH:ko00071] Level1--Metabolism \| Level2--Lipid metabolism \| Level3--Fatty acid degradation |
| BL88-Onlly | *Bifidobacterium longum* | GL001041 | 100 | 0 | blm:BLLJ_0348 | K01897 | ACSL, fadD | long-chain acyl-CoA synthetase | 6.2.1.3 | [PATH:ko02024] Level1--Cellular Processes \| Level2--Cellular community - prokaryotes \| Level3--Quorum sensing;[PATH:ko01100] Level1--Metabolism \| Level2--Global and overview maps \| Level3--Metabolic pathways;[PATH:ko00061] Level1--Metabolism \| Level2--Lipid metabolism \| Level3--Fatty acid biosynthesis;[PATH:ko01212] Level1--Metabolism \| Level2--Global and overview maps \| Level3--Fatty acid metabolism;[PATH:ko00071] Level1--Metabolism \| Level2--Lipid metabolism \| Level3--Fatty acid degradation |
| BL88-Onlly | *Bifidobacterium longum* | GL001211 | 100 | 2.00E-177 | blm:BLLJ_0809 | K13953 | adhP | alcohol dehydrogenase, propanol-preferring | 1.1.1.1 | [PATH:ko00010] Level1--Metabolism \| Level2--Carbohydrate metabolism \| Level3--Glycolysis / Gluconeogenesis;[PATH:ko01120] Level1--Metabolism \| Level2--Global and overview maps \| Level3--Microbial metabolism in diverse environments;[PATH:ko01130] Level1--Metabolism \| Level2--Global and overview maps \| Level3--Biosynthesis of antibiotics;[PATH:ko00350] Level1--Metabolism \| Level2--Amino acid metabolism \| Level3--Tyrosine metabolism;[PATH:ko01100] Level1--Metabolism \| Level2--Global and overview maps \| Level3--Metabolic pathways;[PATH:ko01220] Level1--Metabolism \| Level2--Global and overview maps \| Level3--Degradation of aromatic compounds;[PATH:ko01110] Level1--Metabolism \| Level2--Global and overview maps \| Level3--Biosynthesis of secondary metabolites;[PATH:ko00625] Level1--Metabolism \| Level2--Xenobiotics biodegradation and metabolism \| Level3--Chloroalkane and chloroalkene degradation;[PATH:ko00626] Level1--Metabolism \| Level2--Xenobiotics biodegradation and metabolism \| Level3--Naphthalene degradation;[PATH:ko00071] Level1--Metabolism \| Level2--Lipid metabolism \| Level3--Fatty acid degradation |
| BL88-Onlly | *Bifidobacterium longum* | GL001339 | 100 | 0 | blg:BIL_09030 | K00981 | E2.7.7.41, CDS1, CDS2, cdsA | phosphatidate cytidylyltransferase | 2.7.7.41 | [PATH:ko01100] Level1--Metabolism \| Level2--Global and overview maps \| Level3--Metabolic pathways;[PATH:ko01110] Level1--Metabolism \| Level2--Global and overview maps \| Level3--Biosynthesis of secondary metabolites;[PATH:ko00564] Level1--Metabolism \| Level2--Lipid metabolism \| Level3--Glycerophospholipid metabolism |
| BL88-Onlly | *Bifidobacterium longum* | GL001345 | 100 | 0 | blm:BLLJ_1134 | K01897 | ACSL, fadD | long-chain acyl-CoA synthetase | 6.2.1.3 | [PATH:ko02024] Level1--Cellular Processes \| Level2--Cellular community - prokaryotes \| Level3--Quorum sensing;[PATH:ko01100] Level1--Metabolism \| Level2--Global and overview maps \| Level3--Metabolic pathways;[PATH:ko00061] Level1--Metabolism \| Level2--Lipid metabolism \| Level3--Fatty acid biosynthesis;[PATH:ko01212] Level1--Metabolism \| Level2--Global and overview maps \| Level3--Fatty acid metabolism;[PATH:ko00071] Level1--Metabolism \| Level2--Lipid metabolism \| Level3--Fatty acid degradation |
| BL88-Onlly | *Bifidobacterium longum* | GL001384 | 100 | 2.00E-134 | blm:BLLJ_1179 | K00655 | plsC | 1-acyl-sn-glycerol-3-phosphate acyltransferase | 2.3.1.51 | [PATH:ko01100] Level1--Metabolism \| Level2--Global and overview maps \| Level3--Metabolic pathways;[PATH:ko01110] Level1--Metabolism \| Level2--Global and overview maps \| Level3--Biosynthesis of secondary metabolites;[PATH:ko00564] Level1--Metabolism \| Level2--Lipid metabolism \| Level3--Glycerophospholipid metabolism;[PATH:ko00561] Level1--Metabolism \| Level2--Lipid metabolism \| Level3--Glycerolipid metabolism |
| BL88-Onlly | *Bifidobacterium longum* | GL001501 | 100 | 0 | blm:BLLJ_1730 | K11533 | fas | fatty acid synthase, bacteria type | 2.3.1.- | [PATH:ko01100] Level1--Metabolism \| Level2--Global and overview maps \| Level3--Metabolic pathways;[PATH:ko00061] Level1--Metabolism \| Level2--Lipid metabolism \| Level3--Fatty acid biosynthesis;[PATH:ko01212] Level1--Metabolism \| Level2--Global and overview maps \| Level3--Fatty acid metabolism |
| BB8 | *Bifidobacterium breve* | GL000390 | 100 | 0 | bbrd:BBBR_0354 | K01897 | ACSL, fadD | long-chain acyl-CoA synthetase | 6.2.1.3 | [PATH:ko02024] Level1--Cellular Processes \| Level2--Cellular community - prokaryotes \| Level3--Quorum sensing;[PATH:ko01100] Level1--Metabolism \| Level2--Global and overview maps \| Level3--Metabolic pathways;[PATH:ko00061] Level1--Metabolism \| Level2--Lipid metabolism \| Level3--Fatty acid biosynthesis;[PATH:ko01212] Level1--Metabolism \| Level2--Global and overview maps \| Level3--Fatty acid metabolism;[PATH:ko00071] Level1--Metabolism \| Level2--Lipid metabolism \| Level3--Fatty acid degradation |
| BB8 | *Bifidobacterium breve* | GL000694 | 100 | 3.00E-114 | bbrd:BBBR_0061 | K01048 | pldB | lysophospholipase | 3.1.1.5 | [PATH:ko00564] Level1--Metabolism \| Level2--Lipid metabolism \| Level3--Glycerophospholipid metabolism |
| BB8 | *Bifidobacterium breve* | GL000888 | 100 | 0 | bbrd:BBBR_1488 | K01897 | ACSL, fadD | long-chain acyl-CoA synthetase | 6.2.1.3 | [PATH:ko02024] Level1--Cellular Processes \| Level2--Cellular community - prokaryotes \| Level3--Quorum sensing;[PATH:ko01100] Level1--Metabolism \| Level2--Global and overview maps \| Level3--Metabolic pathways;[PATH:ko00061] Level1--Metabolism \| Level2--Lipid metabolism \| Level3--Fatty acid biosynthesis;[PATH:ko01212] Level1--Metabolism \| Level2--Global and overview maps \| Level3--Fatty acid metabolism;[PATH:ko00071] Level1--Metabolism \| Level2--Lipid metabolism \| Level3--Fatty acid degradation |
| BB8 | *Bifidobacterium breve* | GL001132 | 100 | 0 | bbrd:BBBR_1720 | K11533 | fas | fatty acid synthase, bacteria type | 2.3.1.- | [PATH:ko01100] Level1--Metabolism \| Level2--Global and overview maps \| Level3--Metabolic pathways;[PATH:ko00061] Level1--Metabolism \| Level2--Lipid metabolism \| Level3--Fatty acid biosynthesis;[PATH:ko01212] Level1--Metabolism \| Level2--Global and overview maps \| Level3--Fatty acid metabolism |
| BB8 | *Bifidobacterium breve* | GL001247 | 100 | 0 | bbrd:BBBR_1060 | K01897 | ACSL, fadD | long-chain acyl-CoA synthetase | 6.2.1.3 | [PATH:ko02024] Level1--Cellular Processes \| Level2--Cellular community - prokaryotes \| Level3--Quorum sensing;[PATH:ko01100] Level1--Metabolism \| Level2--Global and overview maps \| Level3--Metabolic pathways;[PATH:ko00061] Level1--Metabolism \| Level2--Lipid metabolism \| Level3--Fatty acid biosynthesis;[PATH:ko01212] Level1--Metabolism \| Level2--Global and overview maps \| Level3--Fatty acid metabolism;[PATH:ko00071] Level1--Metabolism \| Level2--Lipid metabolism \| Level3--Fatty acid degradation |
| BB8 | *Bifidobacterium breve* | GL001253 | 100 | 0 | bbrd:BBBR_1054 | K00981 | E2.7.7.41, CDS1, CDS2, cdsA | phosphatidate cytidylyltransferase | 2.7.7.41 | [PATH:ko01100] Level1--Metabolism \| Level2--Global and overview maps \| Level3--Metabolic pathways;[PATH:ko01110] Level1--Metabolism \| Level2--Global and overview maps \| Level3--Biosynthesis of secondary metabolites;[PATH:ko00564] Level1--Metabolism \| Level2--Lipid metabolism \| Level3--Glycerophospholipid metabolism |
| BB8 | *Bifidobacterium breve* | GL001435 | 100 | 0 | bbrd:BBBR_0882 | K01897 | ACSL, fadD | long-chain acyl-CoA synthetase | 6.2.1.3 | [PATH:ko02024] Level1--Cellular Processes \| Level2--Cellular community - prokaryotes \| Level3--Quorum sensing;[PATH:ko01100] Level1--Metabolism \| Level2--Global and overview maps \| Level3--Metabolic pathways;[PATH:ko00061] Level1--Metabolism \| Level2--Lipid metabolism \| Level3--Fatty acid biosynthesis;[PATH:ko01212] Level1--Metabolism \| Level2--Global and overview maps \| Level3--Fatty acid metabolism;[PATH:ko00071] Level1--Metabolism \| Level2--Lipid metabolism \| Level3--Fatty acid degradation |
| BB8 | *Bifidobacterium breve* | GL001690 | 100 | 3.00E-136 | bbrd:BBBR_1174 | K00655 | plsC | 1-acyl-sn-glycerol-3-phosphate acyltransferase | 2.3.1.51 | [PATH:ko01100] Level1--Metabolism \| Level2--Global and overview maps \| Level3--Metabolic pathways;[PATH:ko01110] Level1--Metabolism \| Level2--Global and overview maps \| Level3--Biosynthesis of secondary metabolites;[PATH:ko00564] Level1--Metabolism \| Level2--Lipid metabolism \| Level3--Glycerophospholipid metabolism;[PATH:ko00561] Level1--Metabolism \| Level2--Lipid metabolism \| Level3--Glycerolipid metabolism |
| LC18 | *Lactobacillus casei* | GL000660 | 78.33 | 3.00E-108 | lrc:LOCK908_1110 | K10804 | tesA | acyl-CoA thioesterase I | 3.1.2.- 3.1.1.5 | [PATH:ko01040] Level1--Metabolism \| Level2--Lipid metabolism \| Level3--Biosynthesis of unsaturated fatty acids |
| LC18 | *Lactobacillus casei* | GL001935 | 88.6 | 8.00E-150 | lrg:LRHM_2036 | K00645 | fabD | [acyl-carrier-protein] S-malonyltransferase | 2.3.1.39 | [PATH:ko01100] Level1--Metabolism \| Level2--Global and overview maps \| Level3--Metabolic pathways;[PATH:ko00061] Level1--Metabolism \| Level2--Lipid metabolism \| Level3--Fatty acid biosynthesis;[PATH:ko01212] Level1--Metabolism \| Level2--Global and overview maps \| Level3--Fatty acid metabolism |
| LC18 | *Lactobacillus casei* | GL000841 | 92.59 | 2.00E-114 | lrc:LOCK908_1668 | K00655 | plsC | 1-acyl-sn-glycerol-3-phosphate acyltransferase | 2.3.1.51 | [PATH:ko01100] Level1--Metabolism \| Level2--Global and overview maps \| Level3--Metabolic pathways;[PATH:ko01110] Level1--Metabolism \| Level2--Global and overview maps \| Level3--Biosynthesis of secondary metabolites;[PATH:ko00564] Level1--Metabolism \| Level2--Lipid metabolism \| Level3--Glycerophospholipid metabolism;[PATH:ko00561] Level1--Metabolism \| Level2--Lipid metabolism \| Level3--Glycerolipid metabolism |
| LC18 | *Lactobacillus casei* | GL001539 | 93.04 | 6.00E-82 | lca:LSEI_1984 | K01073 | E3.1.2.20 | acyl-CoA hydrolase | 3.1.2.20 | -- |
| LC18 | *Lactobacillus casei* | GL001933 | 94.47 | 0 | lrc:LOCK908_2175 | K09458 | fabF | 3-oxoacyl-[acyl-carrier-protein] synthase II | 2.3.1.179 | [PATH:ko01100] Level1--Metabolism \| Level2--Global and overview maps \| Level3--Metabolic pathways;[PATH:ko00061] Level1--Metabolism \| Level2--Lipid metabolism \| Level3--Fatty acid biosynthesis;[PATH:ko01212] Level1--Metabolism \| Level2--Global and overview maps \| Level3--Fatty acid metabolism;[PATH:ko00780] Level1--Metabolism \| Level2--Metabolism of cofactors and vitamins \| Level3--Biotin metabolism |
| LC18 | *Lactobacillus casei* | GL000653 | 95.89 | 1.00E-177 | lca:LSEI_1060 | K07029 | dagK | diacylglycerol kinase (ATP) | 2.7.1.107 | [PATH:ko01100] Level1--Metabolism \| Level2--Global and overview maps \| Level3--Metabolic pathways;[PATH:ko01110] Level1--Metabolism \| Level2--Global and overview maps \| Level3--Biosynthesis of secondary metabolites;[PATH:ko00564] Level1--Metabolism \| Level2--Lipid metabolism \| Level3--Glycerophospholipid metabolism;[PATH:ko00561] Level1--Metabolism \| Level2--Lipid metabolism \| Level3--Glycerolipid metabolism |
| LC18 | *Lactobacillus casei* | GL000833 | 96.18 | 2.00E-134 | lro:LOCK900_1567 | K00981 | E2.7.7.41, CDS1, CDS2, cdsA | phosphatidate cytidylyltransferase | 2.7.7.41 | [PATH:ko01100] Level1--Metabolism \| Level2--Global and overview maps \| Level3--Metabolic pathways;[PATH:ko01110] Level1--Metabolism \| Level2--Global and overview maps \| Level3--Biosynthesis of secondary metabolites;[PATH:ko00564] Level1--Metabolism \| Level2--Lipid metabolism \| Level3--Glycerophospholipid metabolism |
| LC18 | *Lactobacillus casei* | GL001936 | 97.5 | 1.00E-178 | lrc:LOCK908_2178 | K02371 | fabK | enoyl-[acyl-carrier protein] reductase II | 1.3.1.9 | [PATH:ko01100] Level1--Metabolism \| Level2--Global and overview maps \| Level3--Metabolic pathways;[PATH:ko00061] Level1--Metabolism \| Level2--Lipid metabolism \| Level3--Fatty acid biosynthesis;[PATH:ko01212] Level1--Metabolism \| Level2--Global and overview maps \| Level3--Fatty acid metabolism |
| LC18 | *Lactobacillus casei* | GL001940 | 98.62 | 2.00E-77 | lrc:LOCK908_2182 | K02372 | fabZ | 3-hydroxyacyl-[acyl-carrier-protein] dehydratase | 4.2.1.59 | [PATH:ko01100] Level1--Metabolism \| Level2--Global and overview maps \| Level3--Metabolic pathways;[PATH:ko00061] Level1--Metabolism \| Level2--Lipid metabolism \| Level3--Fatty acid biosynthesis;[PATH:ko01212] Level1--Metabolism \| Level2--Global and overview maps \| Level3--Fatty acid metabolism;[PATH:ko00780] Level1--Metabolism \| Level2--Metabolism of cofactors and vitamins \| Level3--Biotin metabolism |
| LC18 | *Lactobacillus casei* | GL001931 | 98.63 | 1.00E-78 | lrc:LOCK908_2173 | K02372 | fabZ | 3-hydroxyacyl-[acyl-carrier-protein] dehydratase | 4.2.1.59 | [PATH:ko01100] Level1--Metabolism \| Level2--Global and overview maps \| Level3--Metabolic pathways;[PATH:ko00061] Level1--Metabolism \| Level2--Lipid metabolism \| Level3--Fatty acid biosynthesis;[PATH:ko01212] Level1--Metabolism \| Level2--Global and overview maps \| Level3--Fatty acid metabolism;[PATH:ko00780] Level1--Metabolism \| Level2--Metabolism of cofactors and vitamins \| Level3--Biotin metabolism |
| LCR15 | *Lactobacillus crispatus* | GL002056 | 90.22 | 5.00E-115 | lae:LBAT_0279 | K02371 | fabK | enoyl-[acyl-carrier protein] reductase II | 1.3.1.9 | [PATH:ko01100] Level1--Metabolism \| Level2--Global and overview maps \| Level3--Metabolic pathways;[PATH:ko00061] Level1--Metabolism \| Level2--Lipid metabolism \| Level3--Fatty acid biosynthesis;[PATH:ko01212] Level1--Metabolism \| Level2--Global and overview maps \| Level3--Fatty acid metabolism |
| LCR15 | *Lactobacillus crispatus* | GL001385 | 91.51 | 3.00E-140 | lay:LAB52_06475 | K00981 | E2.7.7.41, CDS1, CDS2, cdsA | phosphatidate cytidylyltransferase | 2.7.7.41 | [PATH:ko01100] Level1--Metabolism \| Level2--Global and overview maps \| Level3--Metabolic pathways;[PATH:ko01110] Level1--Metabolism \| Level2--Global and overview maps \| Level3--Biosynthesis of secondary metabolites;[PATH:ko00564] Level1--Metabolism \| Level2--Lipid metabolism \| Level3--Glycerophospholipid metabolism |
| LCR15 | *Lactobacillus crispatus* | GL001378 | 99.47 | 9.00E-106 | lcr:LCRIS_01287 | K00655 | plsC | 1-acyl-sn-glycerol-3-phosphate acyltransferase | 2.3.1.51 | [PATH:ko01100] Level1--Metabolism \| Level2--Global and overview maps \| Level3--Metabolic pathways;[PATH:ko01110] Level1--Metabolism \| Level2--Global and overview maps \| Level3--Biosynthesis of secondary metabolites;[PATH:ko00564] Level1--Metabolism \| Level2--Lipid metabolism \| Level3--Glycerophospholipid metabolism;[PATH:ko00561] Level1--Metabolism \| Level2--Lipid metabolism \| Level3--Glycerolipid metabolism |
| LCR15 | *Lactobacillus crispatus* | GL000500 | 99.67 | 7.00E-178 | lcr:LCRIS_00533 | K07029 | dagK | diacylglycerol kinase (ATP) | 2.7.1.107 | [PATH:ko01100] Level1--Metabolism \| Level2--Global and overview maps \| Level3--Metabolic pathways;[PATH:ko01110] Level1--Metabolism \| Level2--Global and overview maps \| Level3--Biosynthesis of secondary metabolites;[PATH:ko00564] Level1--Metabolism \| Level2--Lipid metabolism \| Level3--Glycerophospholipid metabolism;[PATH:ko00561] Level1--Metabolism \| Level2--Lipid metabolism \| Level3--Glycerolipid metabolism |
| LF33 | *Lactobacillus fermentum* | GL000682 | 98.66 | 1.00E-169 | lff:LBFF_0332 | K00645 | fabD | [acyl-carrier-protein] S-malonyltransferase | 2.3.1.39 | [PATH:ko01100] Level1--Metabolism \| Level2--Global and overview maps \| Level3--Metabolic pathways;[PATH:ko00061] Level1--Metabolism \| Level2--Lipid metabolism \| Level3--Fatty acid biosynthesis;[PATH:ko01212] Level1--Metabolism \| Level2--Global and overview maps \| Level3--Fatty acid metabolism |
| LF33 | *Lactobacillus fermentum* | GL001897 | 98.84 | 0 | lff:LBFF_1744 | K13953 | adhP | alcohol dehydrogenase, propanol-preferring | 1.1.1.1 | [PATH:ko00010] Level1--Metabolism \| Level2--Carbohydrate metabolism \| Level3--Glycolysis / Gluconeogenesis;[PATH:ko01120] Level1--Metabolism \| Level2--Global and overview maps \| Level3--Microbial metabolism in diverse environments;[PATH:ko01130] Level1--Metabolism \| Level2--Global and overview maps \| Level3--Biosynthesis of antibiotics;[PATH:ko00350] Level1--Metabolism \| Level2--Amino acid metabolism \| Level3--Tyrosine metabolism;[PATH:ko01100] Level1--Metabolism \| Level2--Global and overview maps \| Level3--Metabolic pathways;[PATH:ko01220] Level1--Metabolism \| Level2--Global and overview maps \| Level3--Degradation of aromatic compounds;[PATH:ko01110] Level1--Metabolism \| Level2--Global and overview maps \| Level3--Biosynthesis of secondary metabolites;[PATH:ko00625] Level1--Metabolism \| Level2--Xenobiotics biodegradation and metabolism \| Level3--Chloroalkane and chloroalkene degradation;[PATH:ko00626] Level1--Metabolism \| Level2--Xenobiotics biodegradation and metabolism \| Level3--Naphthalene degradation;[PATH:ko00071] Level1--Metabolism \| Level2--Lipid metabolism \| Level3--Fatty acid degradation |
| LF33 | *Lactobacillus fermentum* | GL000885 | 98.85 | 5.00E-144 | lor:AYI71_05355 | K00981 | E2.7.7.41, CDS1, CDS2, cdsA | phosphatidate cytidylyltransferase | 2.7.7.41 | [PATH:ko01100] Level1--Metabolism \| Level2--Global and overview maps \| Level3--Metabolic pathways;[PATH:ko01110] Level1--Metabolism \| Level2--Global and overview maps \| Level3--Biosynthesis of secondary metabolites;[PATH:ko00564] Level1--Metabolism \| Level2--Lipid metabolism \| Level3--Glycerophospholipid metabolism |
| LF33 | *Lactobacillus fermentum* | GL000893 | 99.05 | 2.00E-117 | lor:AYI71_05395 | K00655 | plsC | 1-acyl-sn-glycerol-3-phosphate acyltransferase | 2.3.1.51 | [PATH:ko01100] Level1--Metabolism \| Level2--Global and overview maps \| Level3--Metabolic pathways;[PATH:ko01110] Level1--Metabolism \| Level2--Global and overview maps \| Level3--Biosynthesis of secondary metabolites;[PATH:ko00564] Level1--Metabolism \| Level2--Lipid metabolism \| Level3--Glycerophospholipid metabolism;[PATH:ko00561] Level1--Metabolism \| Level2--Lipid metabolism \| Level3--Glycerolipid metabolism |
| LF33 | *Lactobacillus fermentum* | GL000964 | 99.11 | 0 | lff:LBFF_1614 | K07029 | dagK | diacylglycerol kinase (ATP) | 2.7.1.107 | [PATH:ko01100] Level1--Metabolism \| Level2--Global and overview maps \| Level3--Metabolic pathways;[PATH:ko01110] Level1--Metabolism \| Level2--Global and overview maps \| Level3--Biosynthesis of secondary metabolites;[PATH:ko00564] Level1--Metabolism \| Level2--Lipid metabolism \| Level3--Glycerophospholipid metabolism;[PATH:ko00561] Level1--Metabolism \| Level2--Lipid metabolism \| Level3--Glycerolipid metabolism |
| LF33 | *Lactobacillus fermentum* | GL000678 | 99.31 | 8.00E-79 | lor:AYI71_07655 | K02372 | fabZ | 3-hydroxyacyl-[acyl-carrier-protein] dehydratase | 4.2.1.59 | [PATH:ko01100] Level1--Metabolism \| Level2--Global and overview maps \| Level3--Metabolic pathways;[PATH:ko00061] Level1--Metabolism \| Level2--Lipid metabolism \| Level3--Fatty acid biosynthesis;[PATH:ko01212] Level1--Metabolism \| Level2--Global and overview maps \| Level3--Fatty acid metabolism;[PATH:ko00780] Level1--Metabolism \| Level2--Metabolism of cofactors and vitamins \| Level3--Biotin metabolism |
| LF33 | *Lactobacillus fermentum* | GL000357 | 99.37 | 3.00E-180 | lfe:LAF_1637 | K02371 | fabK | enoyl-[acyl-carrier protein] reductase II | 1.3.1.9 | [PATH:ko01100] Level1--Metabolism \| Level2--Global and overview maps \| Level3--Metabolic pathways;[PATH:ko00061] Level1--Metabolism \| Level2--Lipid metabolism \| Level3--Fatty acid biosynthesis;[PATH:ko01212] Level1--Metabolism \| Level2--Global and overview maps \| Level3--Fatty acid metabolism |
| LF33 | *Lactobacillus fermentum* | GL001845 | 99.41 | 0 | lor:AYI71_11130 | K13953 | adhP | alcohol dehydrogenase, propanol-preferring | 1.1.1.1 | [PATH:ko00010] Level1--Metabolism \| Level2--Carbohydrate metabolism \| Level3--Glycolysis / Gluconeogenesis;[PATH:ko01120] Level1--Metabolism \| Level2--Global and overview maps \| Level3--Microbial metabolism in diverse environments;[PATH:ko01130] Level1--Metabolism \| Level2--Global and overview maps \| Level3--Biosynthesis of antibiotics;[PATH:ko00350] Level1--Metabolism \| Level2--Amino acid metabolism \| Level3--Tyrosine metabolism;[PATH:ko01100] Level1--Metabolism \| Level2--Global and overview maps \| Level3--Metabolic pathways;[PATH:ko01220] Level1--Metabolism \| Level2--Global and overview maps \| Level3--Degradation of aromatic compounds;[PATH:ko01110] Level1--Metabolism \| Level2--Global and overview maps \| Level3--Biosynthesis of secondary metabolites;[PATH:ko00625] Level1--Metabolism \| Level2--Xenobiotics biodegradation and metabolism \| Level3--Chloroalkane and chloroalkene degradation;[PATH:ko00626] Level1--Metabolism \| Level2--Xenobiotics biodegradation and metabolism \| Level3--Naphthalene degradation;[PATH:ko00071] Level1--Metabolism \| Level2--Lipid metabolism \| Level3--Fatty acid degradation |
| LF33 | *Lactobacillus fermentum* | GL000680 | 99.5 | 0 | lfe:LAF_0314 | K09458 | fabF | 3-oxoacyl-[acyl-carrier-protein] synthase II | 2.3.1.179 | [PATH:ko01100] Level1--Metabolism \| Level2--Global and overview maps \| Level3--Metabolic pathways;[PATH:ko00061] Level1--Metabolism \| Level2--Lipid metabolism \| Level3--Fatty acid biosynthesis;[PATH:ko01212] Level1--Metabolism \| Level2--Global and overview maps \| Level3--Fatty acid metabolism;[PATH:ko00780] Level1--Metabolism \| Level2--Metabolism of cofactors and vitamins \| Level3--Biotin metabolism |
| LF33 | *Lactobacillus fermentum* | GL000686 | 100 | 6.00E-84 | lfe:LAF_0308 | K02372 | fabZ | 3-hydroxyacyl-[acyl-carrier-protein] dehydratase | 4.2.1.59 | [PATH:ko01100] Level1--Metabolism \| Level2--Global and overview maps \| Level3--Metabolic pathways;[PATH:ko00061] Level1--Metabolism \| Level2--Lipid metabolism \| Level3--Fatty acid biosynthesis;[PATH:ko01212] Level1--Metabolism \| Level2--Global and overview maps \| Level3--Fatty acid metabolism;[PATH:ko00780] Level1--Metabolism \| Level2--Metabolism of cofactors and vitamins \| Level3--Biotin metabolism |
| LG23 | *Lactobacillus gasseri* | GL000792 | 84.35 | 0 | ljn:T285_02840 | K01613 | psd, PISD | phosphatidylserine decarboxylase | 4.1.1.65 | [PATH:ko01100] Level1--Metabolism \| Level2--Global and overview maps \| Level3--Metabolic pathways;[PATH:ko01110] Level1--Metabolism \| Level2--Global and overview maps \| Level3--Biosynthesis of secondary metabolites;[PATH:ko00564] Level1--Metabolism \| Level2--Lipid metabolism \| Level3--Glycerophospholipid metabolism |
| LG23 | *Lactobacillus gasseri* | GL001477 | 98.68 | 9.00E-174 | lga:LGAS_1510 | K07029 | dagK | diacylglycerol kinase (ATP) | 2.7.1.107 | [PATH:ko01100] Level1--Metabolism \| Level2--Global and overview maps \| Level3--Metabolic pathways;[PATH:ko01110] Level1--Metabolism \| Level2--Global and overview maps \| Level3--Biosynthesis of secondary metabolites;[PATH:ko00564] Level1--Metabolism \| Level2--Lipid metabolism \| Level3--Glycerophospholipid metabolism;[PATH:ko00561] Level1--Metabolism \| Level2--Lipid metabolism \| Level3--Glycerolipid metabolism |
| LG23 | *Lactobacillus gasseri* | GL000554 | 99.02 | 5.00E-115 | lga:LGAS_0799 | K00655 | plsC | 1-acyl-sn-glycerol-3-phosphate acyltransferase | 2.3.1.51 | [PATH:ko01100] Level1--Metabolism \| Level2--Global and overview maps \| Level3--Metabolic pathways;[PATH:ko01110] Level1--Metabolism \| Level2--Global and overview maps \| Level3--Biosynthesis of secondary metabolites;[PATH:ko00564] Level1--Metabolism \| Level2--Lipid metabolism \| Level3--Glycerophospholipid metabolism;[PATH:ko00561] Level1--Metabolism \| Level2--Lipid metabolism \| Level3--Glycerolipid metabolism |
| LG23 | *Lactobacillus gasseri* | GL000547 | 99.62 | 4.00E-146 | lga:LGAS_0806 | K00981 | E2.7.7.41, CDS1, CDS2, cdsA | phosphatidate cytidylyltransferase | 2.7.7.41 | [PATH:ko01100] Level1--Metabolism \| Level2--Global and overview maps \| Level3--Metabolic pathways;[PATH:ko01110] Level1--Metabolism \| Level2--Global and overview maps \| Level3--Biosynthesis of secondary metabolites;[PATH:ko00564] Level1--Metabolism \| Level2--Lipid metabolism \| Level3--Glycerophospholipid metabolism |
| LP-Onlly | *Lactobacillus plantarum* | GL000205 | 61.21 | 2.00E-56 | lsa:LCA_0644 | K01073 | E3.1.2.20 | acyl-CoA hydrolase | 3.1.2.20 | -- |
| LP-Onlly | *Lactobacillus plantarum* | GL002419 | 99.03 | 9.00E-175 | lpl:lp_0912 | K02371 | fabK | enoyl-[acyl-carrier protein] reductase II | 1.3.1.9 | [PATH:ko01100] Level1--Metabolism \| Level2--Global and overview maps \| Level3--Metabolic pathways;[PATH:ko00061] Level1--Metabolism \| Level2--Lipid metabolism \| Level3--Fatty acid biosynthesis;[PATH:ko01212] Level1--Metabolism \| Level2--Global and overview maps \| Level3--Fatty acid metabolism |
| LP-Onlly | *Lactobacillus plantarum* | GL000195 | 99.52 | 0 | lpt:zj316_1673 | K09458 | fabF | 3-oxoacyl-[acyl-carrier-protein] synthase II | 2.3.1.179 | [PATH:ko01100] Level1--Metabolism \| Level2--Global and overview maps \| Level3--Metabolic pathways;[PATH:ko00061] Level1--Metabolism \| Level2--Lipid metabolism \| Level3--Fatty acid biosynthesis;[PATH:ko01212] Level1--Metabolism \| Level2--Global and overview maps \| Level3--Fatty acid metabolism;[PATH:ko00780] Level1--Metabolism \| Level2--Metabolism of cofactors and vitamins \| Level3--Biotin metabolism |
| LP-Onlly | *Lactobacillus plantarum* | GL000190 | 100 | 9.00E-82 | lpb:SH83_06930 | K02372 | fabZ | 3-hydroxyacyl-[acyl-carrier-protein] dehydratase | 4.2.1.59 | [PATH:ko01100] Level1--Metabolism \| Level2--Global and overview maps \| Level3--Metabolic pathways;[PATH:ko00061] Level1--Metabolism \| Level2--Lipid metabolism \| Level3--Fatty acid biosynthesis;[PATH:ko01212] Level1--Metabolism \| Level2--Global and overview maps \| Level3--Fatty acid metabolism;[PATH:ko00780] Level1--Metabolism \| Level2--Metabolism of cofactors and vitamins \| Level3--Biotin metabolism |
| LP-Onlly | *Lactobacillus plantarum* | GL000193 | 100 | 3.00E-180 | lpb:SH83_06945 | K00645 | fabD | [acyl-carrier-protein] S-malonyltransferase | 2.3.1.39 | [PATH:ko01100] Level1--Metabolism \| Level2--Global and overview maps \| Level3--Metabolic pathways;[PATH:ko00061] Level1--Metabolism \| Level2--Lipid metabolism \| Level3--Fatty acid biosynthesis;[PATH:ko01212] Level1--Metabolism \| Level2--Global and overview maps \| Level3--Fatty acid metabolism |
| LP-Onlly | *Lactobacillus plantarum* | GL000197 | 100 | 3.00E-74 | lps:LPST_C1334 | K02372 | fabZ | 3-hydroxyacyl-[acyl-carrier-protein] dehydratase | 4.2.1.59 | [PATH:ko01100] Level1--Metabolism \| Level2--Global and overview maps \| Level3--Metabolic pathways;[PATH:ko00061] Level1--Metabolism \| Level2--Lipid metabolism \| Level3--Fatty acid biosynthesis;[PATH:ko01212] Level1--Metabolism \| Level2--Global and overview maps \| Level3--Fatty acid metabolism;[PATH:ko00780] Level1--Metabolism \| Level2--Metabolism of cofactors and vitamins \| Level3--Biotin metabolism |
| LP-Onlly | *Lactobacillus plantarum* | GL000519 | 100 | 1.00E-145 | lpb:SH83_08445 | K00981 | E2.7.7.41, CDS1, CDS2, cdsA | phosphatidate cytidylyltransferase | 2.7.7.41 | [PATH:ko01100] Level1--Metabolism \| Level2--Global and overview maps \| Level3--Metabolic pathways;[PATH:ko01110] Level1--Metabolism \| Level2--Global and overview maps \| Level3--Biosynthesis of secondary metabolites;[PATH:ko00564] Level1--Metabolism \| Level2--Lipid metabolism \| Level3--Glycerophospholipid metabolism |
| LP-Onlly | *Lactobacillus plantarum* | GL000529 | 100 | 1.00E-120 | lpj:JDM1_1722 | K00655 | plsC | 1-acyl-sn-glycerol-3-phosphate acyltransferase | 2.3.1.51 | [PATH:ko01100] Level1--Metabolism \| Level2--Global and overview maps \| Level3--Metabolic pathways;[PATH:ko01110] Level1--Metabolism \| Level2--Global and overview maps \| Level3--Biosynthesis of secondary metabolites;[PATH:ko00564] Level1--Metabolism \| Level2--Lipid metabolism \| Level3--Glycerophospholipid metabolism;[PATH:ko00561] Level1--Metabolism \| Level2--Lipid metabolism \| Level3--Glycerolipid metabolism |
| LP-Onlly | *Lactobacillus plantarum* | GL003077 | 100 | 0 | lpb:SH83_04770 | K07029 | dagK | diacylglycerol kinase (ATP) | 2.7.1.107 | [PATH:ko01100] Level1--Metabolism \| Level2--Global and overview maps \| Level3--Metabolic pathways;[PATH:ko01110] Level1--Metabolism \| Level2--Global and overview maps \| Level3--Biosynthesis of secondary metabolites;[PATH:ko00564] Level1--Metabolism \| Level2--Lipid metabolism \| Level3--Glycerophospholipid metabolism;[PATH:ko00561] Level1--Metabolism \| Level2--Lipid metabolism \| Level3--Glycerolipid metabolism |
| LR22 | *Lactobacillus rhamnosus* | GL002722 | 93.67 | 5.00E-84 | lca:LSEI_1984 | K01073 | E3.1.2.20 | acyl-CoA hydrolase | 3.1.2.20 | -- |
| LR22 | *Lactobacillus rhamnosus* | GL002271 | 94.23 | 7.00E-132 | lrc:LOCK908_1110 | K10804 | tesA | acyl-CoA thioesterase I | 3.1.2.- 3.1.1.5 | [PATH:ko01040] Level1--Metabolism \| Level2--Lipid metabolism \| Level3--Biosynthesis of unsaturated fatty acids |
| LR22 | *Lactobacillus rhamnosus* | GL000874 | 99.62 | 9.00E-145 | lro:LOCK900_1567 | K00981 | E2.7.7.41, CDS1, CDS2, cdsA | phosphatidate cytidylyltransferase | 2.7.7.41 | [PATH:ko01100] Level1--Metabolism \| Level2--Global and overview maps \| Level3--Metabolic pathways;[PATH:ko01110] Level1--Metabolism \| Level2--Global and overview maps \| Level3--Biosynthesis of secondary metabolites;[PATH:ko00564] Level1--Metabolism \| Level2--Lipid metabolism \| Level3--Glycerophospholipid metabolism |
| LR22 | *Lactobacillus rhamnosus* | GL001739 | 99.67 | 2.00E-176 | lrg:LRHM_2036 | K00645 | fabD | [acyl-carrier-protein] S-malonyltransferase | 2.3.1.39 | [PATH:ko01100] Level1--Metabolism \| Level2--Global and overview maps \| Level3--Metabolic pathways;[PATH:ko00061] Level1--Metabolism \| Level2--Lipid metabolism \| Level3--Fatty acid biosynthesis;[PATH:ko01212] Level1--Metabolism \| Level2--Global and overview maps \| Level3--Fatty acid metabolism |
| LR22 | *Lactobacillus rhamnosus* | GL001737 | 99.75 | 0 | lro:LOCK900_2063 | K09458 | fabF | 3-oxoacyl-[acyl-carrier-protein] synthase II | 2.3.1.179 | [PATH:ko01100] Level1--Metabolism \| Level2--Global and overview maps \| Level3--Metabolic pathways;[PATH:ko00061] Level1--Metabolism \| Level2--Lipid metabolism \| Level3--Fatty acid biosynthesis;[PATH:ko01212] Level1--Metabolism \| Level2--Global and overview maps \| Level3--Fatty acid metabolism;[PATH:ko00780] Level1--Metabolism \| Level2--Metabolism of cofactors and vitamins \| Level3--Biotin metabolism |
| LR22 | *Lactobacillus rhamnosus* | GL000411 | 100 | 7.00E-122 | lrg:LRHM_1567 | K00655 | plsC | 1-acyl-sn-glycerol-3-phosphate acyltransferase | 2.3.1.51 | [PATH:ko01100] Level1--Metabolism \| Level2--Global and overview maps \| Level3--Metabolic pathways;[PATH:ko01110] Level1--Metabolism \| Level2--Global and overview maps \| Level3--Biosynthesis of secondary metabolites;[PATH:ko00564] Level1--Metabolism \| Level2--Lipid metabolism \| Level3--Glycerophospholipid metabolism;[PATH:ko00561] Level1--Metabolism \| Level2--Lipid metabolism \| Level3--Glycerolipid metabolism |
| LR22 | *Lactobacillus rhamnosus* | GL001735 | 100 | 7.00E-80 | lrc:LOCK908_2173 | K02372 | fabZ | 3-hydroxyacyl-[acyl-carrier-protein] dehydratase | 4.2.1.59 | [PATH:ko01100] Level1--Metabolism \| Level2--Global and overview maps \| Level3--Metabolic pathways;[PATH:ko00061] Level1--Metabolism \| Level2--Lipid metabolism \| Level3--Fatty acid biosynthesis;[PATH:ko01212] Level1--Metabolism \| Level2--Global and overview maps \| Level3--Fatty acid metabolism;[PATH:ko00780] Level1--Metabolism \| Level2--Metabolism of cofactors and vitamins \| Level3--Biotin metabolism |
| LR22 | *Lactobacillus rhamnosus* | GL001740 | 100 | 0 | lrc:LOCK908_2178 | K02371 | fabK | enoyl-[acyl-carrier protein] reductase II | 1.3.1.9 | [PATH:ko01100] Level1--Metabolism \| Level2--Global and overview maps \| Level3--Metabolic pathways;[PATH:ko00061] Level1--Metabolism \| Level2--Lipid metabolism \| Level3--Fatty acid biosynthesis;[PATH:ko01212] Level1--Metabolism \| Level2--Global and overview maps \| Level3--Fatty acid metabolism |
| LR22 | *Lactobacillus rhamnosus* | GL001744 | 100 | 3.00E-78 | lrc:LOCK908_2182 | K02372 | fabZ | 3-hydroxyacyl-[acyl-carrier-protein] dehydratase | 4.2.1.59 | [PATH:ko01100] Level1--Metabolism \| Level2--Global and overview maps \| Level3--Metabolic pathways;[PATH:ko00061] Level1--Metabolism \| Level2--Lipid metabolism \| Level3--Fatty acid biosynthesis;[PATH:ko01212] Level1--Metabolism \| Level2--Global and overview maps \| Level3--Fatty acid metabolism;[PATH:ko00780] Level1--Metabolism \| Level2--Metabolism of cofactors and vitamins \| Level3--Biotin metabolism |
| LR22 | *Lactobacillus rhamnosus* | GL002263 | 100 | 0 | lra:LRHK_1062 | K07029 | dagK | diacylglycerol kinase (ATP) | 2.7.1.107 | [PATH:ko01100] Level1--Metabolism \| Level2--Global and overview maps \| Level3--Metabolic pathways;[PATH:ko01110] Level1--Metabolism \| Level2--Global and overview maps \| Level3--Biosynthesis of secondary metabolites;[PATH:ko00564] Level1--Metabolism \| Level2--Lipid metabolism \| Level3--Glycerophospholipid metabolism;[PATH:ko00561] Level1--Metabolism \| Level2--Lipid metabolism \| Level3--Glycerolipid metabolism |
| LS86 | *Lactobacillus salivarius* | GL000018 | 98.08 | 3.00E-178 | lsi:HN6_00422 | K00645 | fabD | [acyl-carrier-protein] S-malonyltransferase | 2.3.1.39 | [PATH:ko01100] Level1--Metabolism \| Level2--Global and overview maps \| Level3--Metabolic pathways;[PATH:ko00061] Level1--Metabolism \| Level2--Lipid metabolism \| Level3--Fatty acid biosynthesis;[PATH:ko01212] Level1--Metabolism \| Level2--Global and overview maps \| Level3--Fatty acid metabolism |
| LS86 | *Lactobacillus salivarius* | GL001728 | 98.66 | 7.00E-166 | lsj:LSJ_2148c | K02371 | fabK | enoyl-[acyl-carrier protein] reductase II | 1.3.1.9 | [PATH:ko01100] Level1--Metabolism \| Level2--Global and overview maps \| Level3--Metabolic pathways;[PATH:ko00061] Level1--Metabolism \| Level2--Lipid metabolism \| Level3--Fatty acid biosynthesis;[PATH:ko01212] Level1--Metabolism \| Level2--Global and overview maps \| Level3--Fatty acid metabolism |
| LS86 | *Lactobacillus salivarius* | GL000073 | 99 | 1.00E-110 | lsj:LSJ_0555c | K00655 | plsC | 1-acyl-sn-glycerol-3-phosphate acyltransferase | 2.3.1.51 | [PATH:ko01100] Level1--Metabolism \| Level2--Global and overview maps \| Level3--Metabolic pathways;[PATH:ko01110] Level1--Metabolism \| Level2--Global and overview maps \| Level3--Biosynthesis of secondary metabolites;[PATH:ko00564] Level1--Metabolism \| Level2--Lipid metabolism \| Level3--Glycerophospholipid metabolism;[PATH:ko00561] Level1--Metabolism \| Level2--Lipid metabolism \| Level3--Glycerolipid metabolism |
| LS86 | *Lactobacillus salivarius* | GL001312 | 99.41 | 9.00E-95 | lsl:LSL_1721 | K01073 | E3.1.2.20 | acyl-CoA hydrolase | 3.1.2.20 | -- |
| LS86 | *Lactobacillus salivarius* | GL000014 | 100 | 1.00E-81 | lsj:LSJ_0478 | K02372 | fabZ | 3-hydroxyacyl-[acyl-carrier-protein] dehydratase | 4.2.1.59 | [PATH:ko01100] Level1--Metabolism \| Level2--Global and overview maps \| Level3--Metabolic pathways;[PATH:ko00061] Level1--Metabolism \| Level2--Lipid metabolism \| Level3--Fatty acid biosynthesis;[PATH:ko01212] Level1--Metabolism \| Level2--Global and overview maps \| Level3--Fatty acid metabolism;[PATH:ko00780] Level1--Metabolism \| Level2--Metabolism of cofactors and vitamins \| Level3--Biotin metabolism |
| LS86 | *Lactobacillus salivarius* | GL000020 | 100 | 0 | lsj:LSJ_0484 | K09458 | fabF | 3-oxoacyl-[acyl-carrier-protein] synthase II | 2.3.1.179 | [PATH:ko01100] Level1--Metabolism \| Level2--Global and overview maps \| Level3--Metabolic pathways;[PATH:ko00061] Level1--Metabolism \| Level2--Lipid metabolism \| Level3--Fatty acid biosynthesis;[PATH:ko01212] Level1--Metabolism \| Level2--Global and overview maps \| Level3--Fatty acid metabolism;[PATH:ko00780] Level1--Metabolism \| Level2--Metabolism of cofactors and vitamins \| Level3--Biotin metabolism |
| LS86 | *Lactobacillus salivarius* | GL000022 | 100 | 7.00E-65 | lsl:LSL_0457 | K02372 | fabZ | 3-hydroxyacyl-[acyl-carrier-protein] dehydratase | 4.2.1.59 | [PATH:ko01100] Level1--Metabolism \| Level2--Global and overview maps \| Level3--Metabolic pathways;[PATH:ko00061] Level1--Metabolism \| Level2--Lipid metabolism \| Level3--Fatty acid biosynthesis;[PATH:ko01212] Level1--Metabolism \| Level2--Global and overview maps \| Level3--Fatty acid metabolism;[PATH:ko00780] Level1--Metabolism \| Level2--Metabolism of cofactors and vitamins \| Level3--Biotin metabolism |
| LS86 | *Lactobacillus salivarius* | GL000126 | 100 | 5.00E-146 | lsj:LSJ_0611 | K00981 | E2.7.7.41, CDS1, CDS2, cdsA | phosphatidate cytidylyltransferase | 2.7.7.41 | [PATH:ko01100] Level1--Metabolism \| Level2--Global and overview maps \| Level3--Metabolic pathways;[PATH:ko01110] Level1--Metabolism \| Level2--Global and overview maps \| Level3--Biosynthesis of secondary metabolites;[PATH:ko00564] Level1--Metabolism \| Level2--Lipid metabolism \| Level3--Glycerophospholipid metabolism |
| LS86 | *Lactobacillus salivarius* | GL001719 | 100 | 0 | lsl:LSL_1343 | K07029 | dagK | diacylglycerol kinase (ATP) | 2.7.1.107 | [PATH:ko01100] Level1--Metabolism \| Level2--Global and overview maps \| Level3--Metabolic pathways;[PATH:ko01110] Level1--Metabolism \| Level2--Global and overview maps \| Level3--Biosynthesis of secondary metabolites;[PATH:ko00564] Level1--Metabolism \| Level2--Lipid metabolism \| Level3--Glycerophospholipid metabolism;[PATH:ko00561] Level1--Metabolism \| Level2--Lipid metabolism \| Level3--Glycerolipid metabolism |
