# Supplementary material for: Combined berberine and probiotic treatment as an effective regimen for improving postprandial hyperlipidemia in type 2 diabetes patients: a double blinded placebo controlled randomized study
Source: Gut Microbes. 2021 Dec 20;14(1):2003176. doi: 10.1080/19490976.2021.2003176 (PMC8726654; doi:10.1080/19490976.2021.2003176)
Supplement: Supplemental Material [file KGMI_A_2003176_SM5262.zip › Supplementary information/Data Set 5.docx]

Data Set 5. The associations between the key lipid metabolites and the clinical characteristics in the Prob+BBR group.

|  | TC | | | TG | | | LDL_C | | | TC_2h | | | TG_2h | | | LDL_C_2h | | |
| --- | --- | --- | --- | --- | --- | --- | --- | --- | --- | --- | --- | --- | --- | --- | --- | --- | --- | --- |
|  | estimate | SE | p.value | estimate | SE | p.value | estimate | SE | p.value | estimate | SE | p.value | estimate | SE | p.value | estimate | 2h_SE | p.value |
| FFA 15:0 | 18.33 | 6.45 | 0.00 | 0.29 | 0.09 | 0.00 | 14.96 | 6.00 | 0.01 | 17.54 | 5.58 | 0.00 | 0.12 | 0.05 | 0.02 | 9.78 | 4.07 | 0.02 |
| FFA 22:0 | 10.87 | 3.08 | 0.00 | 0.15 | 0.04 | 0.00 | 8.11 | 2.53 | 0.00 | 8.44 | 3.07 | 0.01 | 0.09 | 0.03 | 0.00 | 3.36 | 2.51 | 0.18 |
| FFA 14:0 | 13.66 | 4.67 | 0.00 | 0.26 | 0.07 | 0.00 | 10.29 | 4.00 | 0.01 | 11.28 | 4.30 | 0.01 | 0.12 | 0.05 | 0.01 | 5.33 | 3.30 | 0.11 |
| FFA 18:1 | 15.98 | 5.58 | 0.00 | 0.16 | 0.07 | 0.01 | 14.20 | 4.43 | 0.00 | 14.43 | 5.24 | 0.01 | 0.09 | 0.05 | 0.06 | 8.18 | 4.00 | 0.04 |
| L-Carnitine | 21.29 | 7.25 | 0.00 | 0.28 | 0.10 | 0.00 | 14.68 | 5.39 | 0.01 | 18.66 | 6.17 | 0.00 | 0.14 | 0.07 | 0.05 | 11.90 | 5.40 | 0.03 |
| FFAo | 21.10 | 7.08 | 0.00 | 0.32 | 0.11 | 0.00 | 17.12 | 5.87 | 0.00 | 18.71 | 6.83 | 0.01 | 0.17 | 0.06 | 0.01 | 9.44 | 5.07 | 0.06 |
| FFA 17:0 | 19.25 | 7.10 | 0.01 | 0.26 | 0.10 | 0.01 | 16.62 | 5.81 | 0.00 | 17.18 | 6.71 | 0.01 | 0.16 | 0.06 | 0.00 | 8.54 | 5.13 | 0.10 |
| FFA 16:0 | 18.43 | 6.62 | 0.01 | 0.19 | 0.07 | 0.01 | 15.88 | 5.44 | 0.00 | 16.70 | 5.77 | 0.00 | 0.14 | 0.05 | 0.00 | 8.68 | 4.81 | 0.07 |
| FFAe | 17.47 | 5.97 | 0.00 | 0.19 | 0.07 | 0.01 | 15.30 | 4.75 | 0.00 | 16.11 | 5.75 | 0.01 | 0.12 | 0.05 | 0.03 | 8.59 | 4.35 | 0.05 |
| FFAs | 17.50 | 5.98 | 0.00 | 0.19 | 0.07 | 0.01 | 15.32 | 4.76 | 0.00 | 16.13 | 5.76 | 0.01 | 0.12 | 0.05 | 0.03 | 8.61 | 4.36 | 0.05 |
| FFA 18:0 | 24.40 | 9.13 | 0.01 | 0.31 | 0.11 | 0.01 | 23.38 | 7.13 | 0.00 | 21.36 | 8.95 | 0.02 | 0.22 | 0.07 | 0.00 | 11.17 | 6.73 | 0.10 |
| PE O-38:6 | 20.16 | 5.15 | 0.00 | -0.03 | 0.06 | 0.67 | 15.40 | 4.06 | 0.00 | 16.39 | 4.99 | 0.00 | 0.03 | 0.06 | 0.56 | 12.47 | 4.00 | 0.00 |
| Hexanoylcarnitine(C6) | 15.36 | 6.22 | 0.01 | 0.30 | 0.08 | 0.00 | 14.09 | 4.73 | 0.00 | 17.60 | 5.32 | 0.00 | 0.16 | 0.06 | 0.01 | 12.12 | 4.00 | 0.00 |
| LPE 18:0 sn-2 | 50.70 | 7.88 | 0.00 | 0.68 | 0.10 | 0.00 | 31.03 | 6.95 | 0.00 | 51.52 | 6.74 | 0.00 | 0.67 | 0.07 | 0.00 | 24.78 | 6.03 | 0.00 |
| PE O-38:7 | 25.95 | 5.08 | 0.00 | 0.01 | 0.07 | 0.85 | 20.78 | 4.40 | 0.00 | 21.52 | 5.26 | 0.00 | 0.03 | 0.06 | 0.62 | 17.38 | 4.01 | 0.00 |
| Decanoylcarnitine(C10) | 17.48 | 5.21 | 0.00 | 0.25 | 0.07 | 0.00 | 13.89 | 3.68 | 0.00 | 17.84 | 4.67 | 0.00 | 0.13 | 0.05 | 0.01 | 12.25 | 3.39 | 0.00 |
| FFA 20:1 | 15.41 | 6.36 | 0.02 | 0.17 | 0.07 | 0.02 | 16.42 | 4.74 | 0.00 | 11.37 | 7.20 | 0.11 | 0.07 | 0.06 | 0.21 | 7.24 | 5.18 | 0.16 |
| LPC 18:0 sn-1 | 61.99 | 11.93 | 0.00 | 0.74 | 0.12 | 0.00 | 38.19 | 8.85 | 0.00 | 66.54 | 9.43 | 0.00 | 0.74 | 0.09 | 0.00 | 33.36 | 8.19 | 0.00 |
| LPC 18:0 sn-2 | 57.11 | 8.79 | 0.00 | 0.61 | 0.10 | 0.00 | 38.62 | 6.53 | 0.00 | 61.74 | 7.34 | 0.00 | 0.61 | 0.08 | 0.00 | 34.42 | 6.33 | 0.00 |
| PC 36:5 | 19.72 | 3.88 | 0.00 | 0.16 | 0.06 | 0.01 | 12.20 | 3.65 | 0.00 | 16.28 | 4.27 | 0.00 | 0.09 | 0.05 | 0.10 | 8.89 | 3.47 | 0.01 |
| Carnitine C12:1 | 15.90 | 5.10 | 0.00 | 0.22 | 0.07 | 0.00 | 13.02 | 3.77 | 0.00 | 15.74 | 4.66 | 0.00 | 0.09 | 0.05 | 0.09 | 11.45 | 3.56 | 0.00 |
