# Supplementary material for: Combined berberine and probiotic treatment as an effective regimen for improving postprandial hyperlipidemia in type 2 diabetes patients: a double blinded placebo controlled randomized study
Source: Gut Microbes. 2021 Dec 20;14(1):2003176. doi: 10.1080/19490976.2021.2003176 (PMC8726654; doi:10.1080/19490976.2021.2003176)
Supplement: Supplemental Material [file KGMI_A_2003176_SM5262.zip › Supplementary information/Data Set 6.docx]

Data Set 6. Correlations between gut microbiota and the levels of cholesterol and triglycerides after Prob+BBR treatment.

|  | fTC | | pTC | | fLDLc | | pLDLc | | fTG | | pTG | | fHDL | | pHDL | |
| --- | --- | --- | --- | --- | --- | --- | --- | --- | --- | --- | --- | --- | --- | --- | --- | --- |
|  | rho | p.value | rho | p.value | rho | p.value.2 | rho | p.value.3 | rho | p.value.4 | rho | p.value.5 | rho | p.value.6 | rho | p.value.7 |
| Bifidobacterium_breve | -0.213 | 0.035 | -0.200 | 0.049 | -0.182 | 0.072 | -0.208 | 0.041 | 0.047 | 0.643 | 0.033 | 0.751 | -0.113 | 0.264 | -0.060 | 0.558 |
| Bifidobacterium_longum | -0.130 | 0.199 | -0.132 | 0.199 | -0.132 | 0.192 | -0.146 | 0.155 | -0.095 | 0.349 | -0.095 | 0.357 | -0.065 | 0.526 | 0.085 | 0.406 |
| unclassified_Erysipelotrichaceae_bacterium_3_1_53 | -0.037 | 0.717 | -0.086 | 0.400 | -0.043 | 0.676 | -0.077 | 0.452 | -0.012 | 0.904 | -0.002 | 0.985 | -0.126 | 0.214 | -0.078 | 0.445 |
| Odoribacter_splanchnicus | -0.029 | 0.775 | -0.033 | 0.748 | -0.042 | 0.680 | 0.035 | 0.732 | -0.157 | 0.120 | -0.171 | 0.094 | 0.063 | 0.539 | -0.001 | 0.989 |
| unclassified_Ruminococcaceae_bacterium_D16 | 0.001 | 0.995 | 0.020 | 0.847 | -0.001 | 0.992 | 0.102 | 0.322 | -0.139 | 0.171 | -0.141 | 0.167 | -0.036 | 0.722 | -0.002 | 0.983 |
| Bacteroides_thetaiotaomicron | -0.129 | 0.202 | -0.097 | 0.347 | -0.127 | 0.211 | -0.067 | 0.517 | -0.084 | 0.407 | -0.104 | 0.310 | -0.044 | 0.669 | -0.086 | 0.405 |
| Bacteroides_plebeius | -0.064 | 0.526 | -0.095 | 0.354 | -0.061 | 0.549 | -0.032 | 0.757 | 0.046 | 0.649 | 0.034 | 0.741 | 0.003 | 0.974 | -0.010 | 0.919 |
| Ruminococcus_bromii | 0.034 | 0.739 | 0.095 | 0.354 | 0.074 | 0.466 | 0.171 | 0.094 | -0.153 | 0.131 | -0.112 | 0.274 | 0.079 | 0.435 | 0.048 | 0.637 |
| Paraprevotella_xylaniphila | 0.047 | 0.642 | 0.131 | 0.199 | 0.088 | 0.386 | 0.125 | 0.221 | -0.035 | 0.733 | 0.030 | 0.768 | -0.008 | 0.938 | -0.034 | 0.739 |
| unclassified_Clostridium_sp._D5 | -0.023 | 0.824 | 0.030 | 0.773 | -0.073 | 0.475 | 0.102 | 0.321 | -0.200 | 0.048 | -0.165 | 0.105 | 0.137 | 0.176 | 0.121 | 0.238 |
| Prevotella_bivia | 0.072 | 0.481 | 0.124 | 0.226 | 0.066 | 0.513 | 0.089 | 0.385 | -0.025 | 0.809 | 0.047 | 0.650 | 0.032 | 0.754 | -0.099 | 0.337 |
| Eubacterium_dolichum | 0.113 | 0.267 | 0.115 | 0.260 | 0.094 | 0.356 | 0.177 | 0.082 | -0.096 | 0.343 | -0.086 | 0.400 | -0.032 | 0.753 | -0.009 | 0.930 |
| Eggerthella_lenta | 0.234 | 0.020 | 0.243 | 0.016 | 0.222 | 0.027 | 0.237 | 0.020 | 0.034 | 0.740 | 0.063 | 0.542 | 0.160 | 0.115 | 0.060 | 0.560 |
| unclassified_Citrobacter_sp._30_2 | 0.024 | 0.813 | -0.015 | 0.883 | 0.033 | 0.745 | -0.035 | 0.731 | 0.028 | 0.785 | -0.008 | 0.937 | 0.023 | 0.818 | 0.011 | 0.913 |
| unclassified_Veillonella_sp._oral_taxon_158 | -0.025 | 0.803 | -0.035 | 0.731 | -0.050 | 0.623 | -0.044 | 0.670 | 0.001 | 0.994 | -0.023 | 0.824 | 0.120 | 0.235 | 0.115 | 0.264 |
| Streptococcus_gordonii | -0.015 | 0.886 | -0.081 | 0.431 | -0.045 | 0.660 | -0.139 | 0.173 | 0.043 | 0.673 | -0.037 | 0.722 | 0.073 | 0.474 | 0.173 | 0.089 |
| Streptococcus_anginosus | 0.101 | 0.322 | 0.057 | 0.577 | 0.086 | 0.400 | 0.018 | 0.863 | 0.102 | 0.313 | 0.033 | 0.748 | -0.004 | 0.967 | 0.057 | 0.581 |
| Lactobacillus_salivarius | -0.042 | 0.683 | -0.060 | 0.561 | 0.034 | 0.736 | -0.056 | 0.588 | 0.155 | 0.126 | 0.070 | 0.495 | -0.188 | 0.063 | -0.121 | 0.239 |
| Lactobacillus_gasseri | -0.085 | 0.404 | -0.089 | 0.383 | -0.098 | 0.333 | -0.144 | 0.161 | 0.263 | 0.009 | 0.177 | 0.083 | -0.158 | 0.119 | -0.116 | 0.256 |
| Lactobacillus_fermentum | -0.163 | 0.107 | -0.191 | 0.060 | -0.122 | 0.228 | -0.165 | 0.106 | 0.047 | 0.645 | 0.006 | 0.957 | -0.225 | 0.025 | -0.116 | 0.259 |
| Lactobacillus_casei | -0.094 | 0.354 | -0.106 | 0.300 | -0.067 | 0.513 | -0.122 | 0.235 | 0.084 | 0.409 | 0.069 | 0.503 | -0.149 | 0.140 | -0.084 | 0.414 |
| Lactobacillus_crispatus | -0.213 | 0.034 | -0.179 | 0.079 | -0.166 | 0.100 | -0.143 | 0.163 | -0.064 | 0.527 | -0.071 | 0.488 | -0.186 | 0.066 | -0.094 | 0.359 |
| Lactobacillus_rhamnosus | -0.149 | 0.140 | -0.129 | 0.206 | -0.115 | 0.258 | -0.134 | 0.190 | 0.068 | 0.507 | 0.053 | 0.609 | -0.205 | 0.042 | -0.144 | 0.160 |
| Lactobacillus_plantarum | -0.110 | 0.278 | -0.119 | 0.244 | -0.100 | 0.327 | -0.134 | 0.190 | 0.023 | 0.820 | 0.022 | 0.831 | -0.149 | 0.141 | -0.067 | 0.517 |
