# Supplementary material for: Combined berberine and probiotic treatment as an effective regimen for improving postprandial hyperlipidemia in type 2 diabetes patients: a double blinded placebo controlled randomized study
Source: Gut Microbes. 2021 Dec 20;14(1):2003176. doi: 10.1080/19490976.2021.2003176 (PMC8726654; doi:10.1080/19490976.2021.2003176)
Supplement: Supplemental Material [file KGMI_A_2003176_SM5262.zip › Supplementary information/Data Set 7.docx]

Data Set 7. The associations between the treatment response to key lipid metabolites and gut microbiota in the Prob+BBR group.

|  | PE O-38_6 | | | LPE 18_0 sn-2 | | | PE O-38_7 | | | Decanoylcarnitine(C10) | | | FFA 20_1 | | | LPC 18_0 sn-1 | | | LPC 18_0 sn-2_ | | | PC 36_5 | | | Carnitine C12:1 | | | FFA 15_0 | | | FFA 22_0 | | | FFA 14_0 | | | FFA 18_1 | | | L-Carnitine_ | | | FFAo | | | FFA 17_0_ | | | FFA 16_0_ | | | FFAe | | | FFAs | | | FFA 18_0 | | |
| --- | --- | --- | --- | --- | --- | --- | --- | --- | --- | --- | --- | --- | --- | --- | --- | --- | --- | --- | --- | --- | --- | --- | --- | --- | --- | --- | --- | --- | --- | --- | --- | --- | --- | --- | --- | --- | --- | --- | --- | --- | --- | --- | --- | --- | --- | --- | --- | --- | --- | --- | --- | --- | --- | --- | --- | --- | --- | --- | --- | --- |
|  | estimate | SE | p.value | estimate | SE | p.value | estimate | SE | p.value | estimate | SE | p.value | estimate | SE | p.value | estimate | SE | p.value | estimate | SE | p.value | estimate | SE | p.value | estimate | SE | p.value | estimate | SE | p.value | estimate | SE | p.value | estimate | SE | p.value | estimate | SE | p.value | estimate | SE | p.value | estimate | SE | p.value | estimate | SE | p.value | estimate | SE | p.value | estimate | SE | p.value | estimate | SE | p.value | estimate | SE | p.value |
| Ruminococcus_bromii | 0.11 | 0.06 | 0.10 | 0.09 | 0.07 | 0.19 | 0.13 | 0.06 | 0.03 | 0.12 | 0.06 | 0.04 | 0.05 | 0.07 | 0.51 | 0.07 | 0.07 | 0.35 | 0.15 | 0.07 | 0.05 | 0.16 | 0.05 | 0.00 | 0.14 | 0.06 | 0.01 | 0.09 | 0.08 | 0.26 | 0.14 | 0.07 | 0.06 | 0.06 | 0.08 | 0.42 | 0.08 | 0.08 | 0.33 | 0.10 | 0.07 | 0.18 | 0.08 | 0.08 | 0.35 | 0.06 | 0.08 | 0.45 | 0.06 | 0.09 | 0.50 | 0.08 | 0.08 | 0.34 | 0.08 | 0.08 | 0.34 | 0.05 | 0.08 | 0.54 |
| unclassified_Ruminococcaceae_bacterium_D16 | 0.13 | 0.05 | 0.01 | 0.04 | 0.06 | 0.54 | 0.17 | 0.06 | 0.00 | 0.21 | 0.05 | 0.00 | 0.18 | 0.07 | 0.01 | 0.07 | 0.06 | 0.26 | 0.14 | 0.07 | 0.05 | 0.13 | 0.06 | 0.02 | 0.21 | 0.05 | 0.00 | 0.10 | 0.06 | 0.11 | 0.22 | 0.07 | 0.00 | 0.15 | 0.06 | 0.02 | 0.18 | 0.07 | 0.01 | 0.12 | 0.08 | 0.13 | 0.14 | 0.07 | 0.04 | 0.12 | 0.07 | 0.08 | 0.09 | 0.07 | 0.19 | 0.16 | 0.07 | 0.02 | 0.16 | 0.07 | 0.02 | 0.12 | 0.07 | 0.09 |
| Streptococcus_anginosus | 0.11 | 0.08 | 0.15 | 0.29 | 0.07 | 0.00 | 0.11 | 0.07 | 0.12 | 0.14 | 0.07 | 0.04 | 0.11 | 0.06 | 0.05 | 0.13 | 0.06 | 0.04 | 0.24 | 0.08 | 0.00 | 0.02 | 0.07 | 0.80 | 0.12 | 0.06 | 0.04 | 0.06 | 0.06 | 0.31 | 0.14 | 0.07 | 0.05 | 0.05 | 0.06 | 0.40 | 0.10 | 0.06 | 0.13 | 0.10 | 0.07 | 0.18 | 0.09 | 0.06 | 0.11 | 0.09 | 0.06 | 0.09 | 0.08 | 0.06 | 0.17 | 0.10 | 0.06 | 0.10 | 0.10 | 0.06 | 0.10 | 0.10 | 0.05 | 0.05 |
| Streptococcus_gordonii | 0.09 | 0.07 | 0.23 | 0.16 | 0.06 | 0.01 | 0.04 | 0.07 | 0.58 | 0.08 | 0.07 | 0.23 | 0.05 | 0.06 | 0.40 | 0.10 | 0.07 | 0.19 | 0.06 | 0.07 | 0.37 | -0.06 | 0.07 | 0.37 | 0.03 | 0.07 | 0.67 | 0.03 | 0.05 | 0.58 | 0.10 | 0.05 | 0.07 | 0.05 | 0.06 | 0.37 | 0.07 | 0.05 | 0.20 | -0.01 | 0.07 | 0.85 | 0.06 | 0.05 | 0.23 | 0.08 | 0.05 | 0.13 | 0.10 | 0.06 | 0.06 | 0.08 | 0.05 | 0.13 | 0.08 | 0.05 | 0.13 | 0.08 | 0.06 | 0.13 |
| Bacteroides_thetaiotaomicron | -0.14 | 0.07 | 0.05 | -0.08 | 0.05 | 0.15 | -0.09 | 0.07 | 0.20 | -0.06 | 0.06 | 0.33 | 0.01 | 0.07 | 0.85 | -0.08 | 0.07 | 0.26 | -0.05 | 0.07 | 0.47 | -0.04 | 0.06 | 0.54 | -0.04 | 0.07 | 0.55 | 0.06 | 0.07 | 0.38 | 0.02 | 0.06 | 0.77 | 0.04 | 0.06 | 0.49 | 0.00 | 0.06 | 0.94 | 0.12 | 0.08 | 0.12 | 0.04 | 0.07 | 0.58 | 0.00 | 0.07 | 0.95 | -0.02 | 0.07 | 0.81 | 0.02 | 0.07 | 0.78 | 0.02 | 0.07 | 0.78 | -0.01 | 0.07 | 0.91 |
| Paraprevotella_xylaniphila | 0.07 | 0.07 | 0.33 | 0.03 | 0.06 | 0.58 | 0.03 | 0.07 | 0.66 | 0.16 | 0.07 | 0.02 | 0.04 | 0.07 | 0.53 | 0.08 | 0.06 | 0.21 | 0.12 | 0.07 | 0.07 | -0.04 | 0.08 | 0.58 | 0.15 | 0.07 | 0.04 | 0.02 | 0.06 | 0.72 | 0.06 | 0.06 | 0.28 | 0.06 | 0.06 | 0.37 | 0.07 | 0.07 | 0.26 | 0.02 | 0.07 | 0.75 | 0.06 | 0.06 | 0.36 | 0.05 | 0.06 | 0.43 | 0.03 | 0.06 | 0.68 | 0.06 | 0.07 | 0.34 | 0.06 | 0.07 | 0.34 | 0.02 | 0.06 | 0.70 |
| unclassified_Clostridium_sp._D5 | 0.05 | 0.06 | 0.38 | 0.01 | 0.05 | 0.89 | 0.10 | 0.06 | 0.12 | 0.14 | 0.05 | 0.01 | 0.15 | 0.07 | 0.02 | -0.07 | 0.07 | 0.31 | 0.00 | 0.07 | 0.97 | 0.01 | 0.07 | 0.93 | 0.11 | 0.06 | 0.05 | 0.14 | 0.05 | 0.01 | 0.18 | 0.05 | 0.00 | 0.16 | 0.05 | 0.00 | 0.19 | 0.06 | 0.00 | 0.06 | 0.07 | 0.39 | 0.16 | 0.06 | 0.00 | 0.17 | 0.06 | 0.01 | 0.17 | 0.06 | 0.01 | 0.18 | 0.06 | 0.00 | 0.18 | 0.06 | 0.00 | 0.16 | 0.06 | 0.01 |
| unclassified_Veillonella_sp._oral_taxon_158 | 0.04 | 0.07 | 0.55 | 0.03 | 0.08 | 0.65 | 0.06 | 0.07 | 0.44 | 0.04 | 0.07 | 0.51 | 0.07 | 0.08 | 0.40 | 0.01 | 0.07 | 0.94 | 0.01 | 0.08 | 0.92 | 0.08 | 0.08 | 0.33 | 0.08 | 0.07 | 0.22 | 0.05 | 0.07 | 0.52 | 0.15 | 0.07 | 0.04 | 0.07 | 0.06 | 0.31 | 0.11 | 0.07 | 0.13 | -0.12 | 0.09 | 0.18 | 0.07 | 0.07 | 0.33 | 0.08 | 0.07 | 0.27 | 0.11 | 0.06 | 0.08 | 0.11 | 0.07 | 0.10 | 0.11 | 0.07 | 0.10 | 0.09 | 0.07 | 0.21 |
| Odoribacter_splanchnicus | 0.08 | 0.07 | 0.29 | -0.10 | 0.07 | 0.14 | 0.05 | 0.07 | 0.51 | 0.12 | 0.07 | 0.08 | -0.01 | 0.07 | 0.83 | -0.11 | 0.08 | 0.13 | -0.07 | 0.08 | 0.38 | 0.04 | 0.06 | 0.54 | 0.17 | 0.07 | 0.02 | -0.04 | 0.07 | 0.56 | -0.08 | 0.06 | 0.20 | -0.05 | 0.06 | 0.39 | -0.07 | 0.07 | 0.32 | 0.20 | 0.07 | 0.00 | -0.03 | 0.07 | 0.66 | -0.06 | 0.07 | 0.39 | -0.12 | 0.07 | 0.08 | -0.08 | 0.07 | 0.26 | -0.08 | 0.07 | 0.26 | -0.07 | 0.07 | 0.29 |
| Eggerthella_lenta | 0.17 | 0.07 | 0.01 | 0.23 | 0.05 | 0.00 | 0.16 | 0.07 | 0.02 | 0.19 | 0.05 | 0.00 | 0.07 | 0.06 | 0.22 | 0.18 | 0.07 | 0.01 | 0.20 | 0.07 | 0.00 | 0.13 | 0.06 | 0.02 | 0.14 | 0.05 | 0.01 | 0.18 | 0.07 | 0.01 | 0.18 | 0.06 | 0.00 | 0.14 | 0.06 | 0.03 | 0.12 | 0.06 | 0.06 | 0.10 | 0.08 | 0.25 | 0.16 | 0.06 | 0.01 | 0.12 | 0.06 | 0.05 | 0.12 | 0.07 | 0.08 | 0.13 | 0.06 | 0.03 | 0.13 | 0.06 | 0.03 | 0.10 | 0.06 | 0.09 |
| Bacteroides_plebeius | 0.07 | 0.06 | 0.26 | -0.02 | 0.06 | 0.78 | 0.03 | 0.07 | 0.63 | 0.05 | 0.07 | 0.52 | -0.11 | 0.06 | 0.06 | 0.01 | 0.07 | 0.87 | 0.01 | 0.07 | 0.92 | 0.10 | 0.07 | 0.15 | 0.07 | 0.06 | 0.28 | -0.01 | 0.05 | 0.86 | -0.02 | 0.06 | 0.73 | -0.06 | 0.05 | 0.27 | 0.00 | 0.05 | 0.97 | 0.01 | 0.07 | 0.88 | -0.02 | 0.05 | 0.74 | 0.01 | 0.05 | 0.93 | 0.03 | 0.05 | 0.56 | -0.01 | 0.06 | 0.81 | -0.01 | 0.06 | 0.81 | -0.02 | 0.06 | 0.75 |
| unclassified_Erysipelotrichaceae_bacterium_3_1_53 | -0.13 | 0.07 | 0.07 | -0.13 | 0.06 | 0.03 | -0.08 | 0.07 | 0.28 | 0.06 | 0.08 | 0.44 | 0.12 | 0.08 | 0.13 | -0.09 | 0.06 | 0.15 | -0.04 | 0.07 | 0.54 | -0.03 | 0.07 | 0.62 | 0.11 | 0.07 | 0.15 | 0.00 | 0.06 | 0.98 | 0.12 | 0.07 | 0.10 | 0.08 | 0.07 | 0.26 | 0.08 | 0.07 | 0.25 | -0.11 | 0.11 | 0.32 | 0.06 | 0.07 | 0.39 | 0.06 | 0.06 | 0.31 | 0.04 | 0.06 | 0.55 | 0.09 | 0.07 | 0.19 | 0.09 | 0.07 | 0.19 | 0.08 | 0.06 | 0.21 |
| Prevotella_bivia | 0.11 | 0.08 | 0.13 | 0.08 | 0.07 | 0.24 | 0.08 | 0.07 | 0.25 | 0.15 | 0.07 | 0.03 | -0.02 | 0.06 | 0.79 | 0.10 | 0.07 | 0.14 | 0.11 | 0.07 | 0.10 | 0.01 | 0.08 | 0.86 | 0.15 | 0.08 | 0.05 | -0.06 | 0.07 | 0.41 | -0.05 | 0.07 | 0.46 | -0.07 | 0.07 | 0.29 | -0.03 | 0.06 | 0.64 | 0.02 | 0.08 | 0.75 | -0.04 | 0.06 | 0.55 | -0.01 | 0.06 | 0.87 | -0.02 | 0.06 | 0.72 | -0.03 | 0.06 | 0.65 | -0.03 | 0.06 | 0.65 | -0.01 | 0.06 | 0.92 |
| Eubacterium_dolichum | 0.22 | 0.05 | 0.00 | 0.13 | 0.05 | 0.01 | 0.25 | 0.05 | 0.00 | 0.15 | 0.05 | 0.00 | 0.13 | 0.08 | 0.13 | 0.17 | 0.07 | 0.02 | 0.18 | 0.07 | 0.01 | 0.18 | 0.06 | 0.00 | 0.16 | 0.05 | 0.00 | 0.13 | 0.06 | 0.03 | 0.13 | 0.07 | 0.07 | 0.14 | 0.07 | 0.06 | 0.14 | 0.07 | 0.04 | 0.24 | 0.07 | 0.00 | 0.14 | 0.07 | 0.03 | 0.13 | 0.06 | 0.04 | 0.10 | 0.07 | 0.14 | 0.13 | 0.07 | 0.05 | 0.13 | 0.07 | 0.05 | 0.12 | 0.07 | 0.10 |
| unclassified_Citrobacter_sp._30_2 | -0.11 | 0.07 | 0.13 | -0.07 | 0.05 | 0.14 | -0.13 | 0.07 | 0.06 | -0.19 | 0.05 | 0.00 | 0.02 | 0.07 | 0.76 | 0.03 | 0.07 | 0.65 | -0.02 | 0.06 | 0.73 | -0.16 | 0.06 | 0.01 | -0.18 | 0.06 | 0.00 | -0.08 | 0.08 | 0.27 | -0.14 | 0.07 | 0.04 | -0.12 | 0.07 | 0.06 | -0.04 | 0.06 | 0.49 | -0.20 | 0.07 | 0.00 | -0.05 | 0.07 | 0.44 | -0.03 | 0.06 | 0.64 | -0.04 | 0.06 | 0.51 | -0.05 | 0.06 | 0.46 | -0.05 | 0.06 | 0.46 | -0.02 | 0.07 | 0.80 |
| Bifidobacterium_breve | -0.25 | 0.07 | 0.00 | -0.13 | 0.06 | 0.02 | -0.18 | 0.07 | 0.01 | -0.13 | 0.06 | 0.04 | -0.09 | 0.08 | 0.22 | -0.15 | 0.06 | 0.01 | -0.19 | 0.07 | 0.00 | -0.14 | 0.06 | 0.02 | -0.09 | 0.06 | 0.12 | -0.09 | 0.06 | 0.17 | -0.09 | 0.07 | 0.17 | -0.11 | 0.07 | 0.13 | -0.12 | 0.07 | 0.11 | -0.07 | 0.07 | 0.34 | -0.11 | 0.07 | 0.11 | -0.10 | 0.07 | 0.12 | -0.11 | 0.07 | 0.10 | -0.11 | 0.07 | 0.11 | -0.11 | 0.07 | 0.11 | -0.10 | 0.07 | 0.12 |
| Bifidobacterium_longum | 0.06 | 0.06 | 0.27 | 0.12 | 0.05 | 0.02 | 0.05 | 0.06 | 0.35 | 0.14 | 0.04 | 0.00 | 0.05 | 0.06 | 0.39 | 0.08 | 0.07 | 0.30 | 0.06 | 0.06 | 0.36 | 0.08 | 0.06 | 0.21 | 0.16 | 0.05 | 0.00 | 0.13 | 0.07 | 0.08 | 0.15 | 0.07 | 0.02 | 0.10 | 0.08 | 0.20 | 0.13 | 0.07 | 0.05 | 0.07 | 0.06 | 0.23 | 0.11 | 0.07 | 0.11 | 0.13 | 0.07 | 0.05 | 0.16 | 0.07 | 0.02 | 0.11 | 0.07 | 0.10 | 0.11 | 0.07 | 0.10 | 0.15 | 0.06 | 0.02 |
| Lactobacillus_casei | -0.20 | 0.07 | 0.01 | -0.16 | 0.05 | 0.00 | -0.17 | 0.07 | 0.01 | -0.22 | 0.05 | 0.00 | -0.13 | 0.07 | 0.08 | -0.16 | 0.06 | 0.01 | -0.15 | 0.06 | 0.01 | -0.17 | 0.06 | 0.00 | -0.20 | 0.05 | 0.00 | -0.14 | 0.07 | 0.04 | -0.16 | 0.07 | 0.02 | -0.16 | 0.06 | 0.01 | -0.17 | 0.07 | 0.02 | -0.16 | 0.07 | 0.02 | -0.14 | 0.07 | 0.03 | -0.14 | 0.07 | 0.05 | -0.17 | 0.07 | 0.02 | -0.16 | 0.07 | 0.02 | -0.16 | 0.07 | 0.02 | -0.15 | 0.07 | 0.03 |
| Lactobacillus_crispatus | -0.21 | 0.07 | 0.00 | -0.13 | 0.07 | 0.04 | -0.15 | 0.07 | 0.02 | -0.17 | 0.06 | 0.01 | -0.02 | 0.08 | 0.78 | -0.09 | 0.07 | 0.17 | -0.10 | 0.07 | 0.16 | -0.17 | 0.05 | 0.00 | -0.12 | 0.06 | 0.05 | -0.07 | 0.07 | 0.34 | -0.07 | 0.07 | 0.29 | -0.09 | 0.07 | 0.23 | -0.04 | 0.07 | 0.61 | -0.12 | 0.06 | 0.04 | -0.05 | 0.07 | 0.47 | -0.02 | 0.07 | 0.76 | -0.05 | 0.06 | 0.40 | -0.04 | 0.07 | 0.55 | -0.04 | 0.07 | 0.54 | -0.04 | 0.07 | 0.58 |
| Lactobacillus_fermentum | -0.19 | 0.07 | 0.01 | -0.12 | 0.05 | 0.01 | -0.16 | 0.07 | 0.02 | -0.23 | 0.06 | 0.00 | -0.19 | 0.08 | 0.02 | -0.12 | 0.06 | 0.05 | -0.16 | 0.05 | 0.00 | -0.21 | 0.06 | 0.00 | -0.19 | 0.06 | 0.00 | -0.26 | 0.08 | 0.00 | -0.24 | 0.07 | 0.00 | -0.25 | 0.07 | 0.00 | -0.24 | 0.07 | 0.00 | -0.16 | 0.08 | 0.03 | -0.25 | 0.07 | 0.00 | -0.21 | 0.07 | 0.00 | -0.21 | 0.07 | 0.00 | -0.23 | 0.07 | 0.00 | -0.23 | 0.07 | 0.00 | -0.18 | 0.07 | 0.01 |
| Lactobacillus_plantarum | -0.22 | 0.06 | 0.00 | -0.17 | 0.05 | 0.00 | -0.20 | 0.06 | 0.00 | -0.17 | 0.06 | 0.00 | -0.09 | 0.07 | 0.20 | -0.18 | 0.05 | 0.00 | -0.21 | 0.05 | 0.00 | -0.18 | 0.05 | 0.00 | -0.14 | 0.06 | 0.01 | -0.15 | 0.07 | 0.03 | -0.17 | 0.07 | 0.01 | -0.18 | 0.07 | 0.01 | -0.15 | 0.07 | 0.04 | -0.20 | 0.08 | 0.01 | -0.15 | 0.07 | 0.03 | -0.14 | 0.06 | 0.03 | -0.16 | 0.07 | 0.02 | -0.14 | 0.07 | 0.05 | -0.14 | 0.07 | 0.05 | -0.14 | 0.07 | 0.03 |
| Lactobacillus_rhamnosus | -0.26 | 0.06 | 0.00 | -0.17 | 0.05 | 0.00 | -0.22 | 0.06 | 0.00 | -0.17 | 0.05 | 0.00 | -0.12 | 0.08 | 0.15 | -0.18 | 0.05 | 0.00 | -0.20 | 0.05 | 0.00 | -0.19 | 0.05 | 0.00 | -0.15 | 0.06 | 0.01 | -0.15 | 0.08 | 0.05 | -0.16 | 0.07 | 0.02 | -0.18 | 0.08 | 0.02 | -0.17 | 0.08 | 0.03 | -0.15 | 0.07 | 0.04 | -0.16 | 0.08 | 0.05 | -0.15 | 0.08 | 0.06 | -0.18 | 0.08 | 0.02 | -0.16 | 0.08 | 0.04 | -0.16 | 0.08 | 0.04 | -0.15 | 0.08 | 0.05 |
| Lactobacillus_salivarius | -0.06 | 0.07 | 0.41 | -0.08 | 0.05 | 0.15 | -0.05 | 0.06 | 0.40 | -0.07 | 0.07 | 0.28 | -0.05 | 0.08 | 0.51 | 0.00 | 0.06 | 0.99 | -0.08 | 0.06 | 0.22 | -0.10 | 0.06 | 0.08 | -0.04 | 0.07 | 0.55 | -0.10 | 0.06 | 0.12 | -0.02 | 0.06 | 0.74 | -0.06 | 0.06 | 0.37 | -0.06 | 0.07 | 0.36 | -0.06 | 0.06 | 0.35 | -0.07 | 0.06 | 0.25 | -0.06 | 0.06 | 0.30 | -0.07 | 0.06 | 0.25 | -0.05 | 0.06 | 0.41 | -0.05 | 0.06 | 0.41 | -0.06 | 0.06 | 0.31 |
| Lactobacillus_gasseri | -0.22 | 0.06 | 0.00 | -0.15 | 0.05 | 0.00 | -0.20 | 0.06 | 0.00 | -0.09 | 0.05 | 0.09 | -0.04 | 0.08 | 0.58 | -0.16 | 0.05 | 0.00 | -0.17 | 0.05 | 0.00 | -0.19 | 0.06 | 0.00 | -0.07 | 0.05 | 0.17 | -0.18 | 0.08 | 0.03 | -0.12 | 0.07 | 0.10 | -0.16 | 0.07 | 0.03 | -0.13 | 0.07 | 0.06 | -0.15 | 0.06 | 0.01 | -0.14 | 0.07 | 0.05 | -0.11 | 0.06 | 0.09 | -0.14 | 0.06 | 0.02 | -0.12 | 0.07 | 0.08 | -0.12 | 0.07 | 0.08 | -0.09 | 0.06 | 0.14 |
