# Supplementary material for: Combined berberine and probiotic treatment as an effective regimen for improving postprandial hyperlipidemia in type 2 diabetes patients: a double blinded placebo controlled randomized study
Source: Gut Microbes. 2021 Dec 20;14(1):2003176. doi: 10.1080/19490976.2021.2003176 (PMC8726654; doi:10.1080/19490976.2021.2003176)
Supplement: Supplemental Material [file KGMI_A_2003176_SM5262.zip › Supplementary information/Data Set 8 revised.docx]

Data Set 8. Comparison of baseline RAs of 24 microbial species shown in Figure 3C and genus Bifidobacterium and between participants with dyslipidemia (n=171) and eulipidemia (n=194) at baseline

|  | **P-value (Wilcoxon Rank-sum test)** | **Occurrence** | | **Median RA** | | **Z-score** |
| --- | --- | --- | --- | --- | --- | --- |
|  |  | **Dyslipdemia** | **Eulipdemia** | **Dyslipdemia** | **Eulipdemia** |  |
| **Species** |  |  |  |  |  |  |
| *Bifidobacterium breve* | 0.007399585 | 99.43% | 100.00% | 1.51E-06 | 2.79E-06 | -2.43726 |
| *Bifidobacterium adolescentis* | 0.092067144 | 97.13% | 95.81% | 4.50E-06 | 7.89E-06 | -1.32813 |
| *Bifidobacterium angulatum* | 0.498279082 | 91.95% | 89.01% | 5.48E-07 | 6.71E-07 | -0.00431 |
| *Bifidobacterium animalis* | 0.061304132 | 97.70% | 98.43% | 8.68E-07 | 1.06E-06 | -1.54392 |
| *Bifidobacterium bifidum* | 0.321777979 | 95.98% | 95.29% | 8.98E-07 | 9.79E-07 | -0.46273 |
| *Bifidobacterium catenulatum.Bifidobacterium pseudocatenulatum complex* | 0.119017762 | 100.00% | 100.00% | 3.12E-05 | 4.64E-05 | -1.17991 |
| *Bifidobacterium dentium* | 0.104584225 | 90.80% | 91.62% | 1.39E-06 | 2.06E-06 | -1.25586 |
| *Bifidobacterium longum* | 0.009711622 | 100.00% | 100.00% | 4.85E-05 | 8.59E-05 | -2.33731 |
| *unclassified Erysipelotrichaceae bacterium 3_ 1_53* | 0.008890721 | 100.00% | 100.00% | 6.53E-05 | 8.87E-05 | -2.37014 |
| *Odoribacter splanchnicus* | 0.027383351 | 100.00% | 100.00% | 0.001266001 | 0.001953765 | -2.33731 |
| *unclassified Ruminococcaceae bacterium D16* | 0.036015389 | 100.00% | 100.00% | 0.000134561 | 0.000171562 | -1.79892 |
| *unclassified Clostridium sp. D5* | 0.054238524 | 100.00% | 100.00% | 2.89E-05 | 3.77E-05 | -1.60508 |
| *Lactobacillus crispatus* | 0.087168203 | 44.25% | 50.79% | 0 | 3.64E-09 | -1.3584 |
| *Bacteroides plebeius* | 0.113819807 | 100.00% | 100.00% | 0.00146337 | 0.00090534 | 1.20646 |
| *Lactobacillus fermentum* | 0.181202623 | 79.89% | 86.39% | 5.06E-08 | 7.65E-08 | -0.91079 |
| *Lactobacillus casei* | 0.240090254 | 78.16% | 78.01% | 2.52E-08 | 3.65E-08 | -0.70601 |
| *Lactobacillus rhamnosus* | 0.254170074 | 8.62% | 12.04% | 0 | 0 | -0.66142 |
| *Ruminococcus bromii* | 0.279187316 | 100.00% | 100.00% | 0.000253647 | 0.000352887 | -0.58526 |
| *Eggerthella lenta* | 0.314097763 | 100.00% | 100.00% | 1.93E-05 | 3.05E-05 | -0.48427 |
| *Lactobacillus plantarum* | 0.337331144 | 23.56% | 18.85% | 0 | 0 | 0.41976 |
| *unclassified Citrobacter sp. 30 2* | 0.387789661 | 100.00% | 98.95% | 6.19E-06 | 4.48E-06 | 0.28508 |
| *unclassified Veillonella sp. oral taxon 158* | 0.410558666 | 100.00% | 100.00% | 3.70E-06 | 4.31E-06 | -0.22611 |
| *Lactobacillus salivarius* | 0.483106941 | 93.10% | 94.76% | 7.62E-08 | 8.56E-08 | -0.04236 |
| *Bacteroides thetaiotaomicron* | 0.510200805 | 100.00% | 100.00% | 0.00637763 | 0.006515136 | 0.02557 |
| *Streptococcus gordonii* | 0.621204147 | 100.00% | 100.00% | 2.33E-06 | 2.03E-06 | -0.30864 |
| *Paraprevotella xylaniphila* | 0.648460319 | 100.00% | 100.00% | 0.000162604 | 0.000162382 | -0.38117 |
| *Prevotella bivia* | 0.734462545 | 100.00% | 100.00% | 3.57E-05 | 3.43E-05 | -0.62637 |
| *Eubacterium dolichum* | 0.764584348 | 100.00% | 100.00% | 3.08E-05 | 3.36E-05 | 0.72113 |
| *Lactobacillus gasseri* | 0.79274639 | 85.63% | 82.72% | 8.27E-08 | 7.00E-08 | -0.81599 |
| *Streptococcus anginosus* | 0.882346583 | 100.00% | 100.00% | 2.09E-06 | 2.04E-06 | 1.1868 |
| **Genus** |  |  |  |  |  |  |
| *Bifidobacterium* | 1.36E-02 | 100.00% | 100.00% | 2.24E-04 | 3.44E-04 | -2.47 |

The p-value and Z-score were calculated with the Wilcoxon signed rank test between dyslipidemia and eulipidemia groups. A Z-score >0 indicated an increase of RA of the species in participants with dyslipidemia, and a Z-score <0 indicated a decrease in participants with eulipidemia. B. catenulatum-Bpc: Bifidobacterium_catenulatum-Bifidobacterium_pseudocatenulatum_complex.
